# Cytotoxicity against A549 Human Lung Cancer Cell Line via the Mitochondrial Membrane Potential and Nuclear Condensation Effects of *Nepeta paulsenii* Briq., a Perennial Herb

**DOI:** 10.3390/molecules28062812

**Published:** 2023-03-20

**Authors:** Aqsa Hanif, Ahmad H. Ibrahim, Sidra Ismail, Sawsan S. Al-Rawi, Jam Nazeer Ahmad, Mansoor Hameed, Ghulam Mustufa, Samina Tanwir

**Affiliations:** 1 Department of Botany, University of Agriculture Faisalabad, Faisalabad 38000, Pakistan; 2 Pharmacy Department, Faculty of Pharmacy, Tishk International University, 100mt. St., Near Baz Interaction, Erbil 44001, KRG, Iraq; 3 Incharge Health Officer, BHU 418 GB, Faisalabad 37150, Pakistan; 4 Biology Education Department, Faculty of Education, Tishk International University, 100mt. St., Near Baz Interaction, Erbil 44001, KRG, Iraq; 5 Department of Entomology, University of Agriculture Faisalabad, Faisalabad 38000, Pakistan; 6 Centre of Agricultural Biochemistry and Biotechnology (CABB), University of Agriculture Faisalabad, Faisalabad 38000, Pakistan

**Keywords:** *Nepeta paulsenii* Briq., A459, adenocarcinomic human alveolar basal epithelial cells, lung cancer, in vitro, cytotoxicity

## Abstract

The genus *Nepeta* belongs to the largest Lamiaceae family, with 300 species, which are distributed throughout the various regions of Africa, Asia, India, and America. Along with other plant families distinguished by their medicinal and therapeutic values, the *Nepeta* genus of Lameaceae remains relatively valuable. Hence, the phytochemicals of *N. paulsenii* Briq. were extracted using different plant parts, i.e., leaves, stem, roots, flowers, and the whole plant by using various solvents (ethanol, water, and ethyl acetate), obtaining 15 fractions. Each extract of dried plant material was analyzed by FT-IR and GC-MS to identify the chemical constituents. The cytotoxicity of each fraction was analyzed by MTT assay and mitochondrial membrane potential and nuclear condensation assays against lung cancer cells. Among the ethyl acetate and ethanolic extracts, the flowers showed the best results, with IC_50_ values of 51.57 μg/mL and 50.58 μg/mL, respectively. In contrast, among the water extracts of the various plant segments, the stem showed the best results, with an IC_50_ value of 123.80 μg/mL. 5-flourouracil was used as the standard drug, providing an IC_50_ value of 83.62 μg/mL. The Hoechst 33342 stain results indicated apoptotic features, i.e., chromatin dissolution and broken down, fragmented, and crescent-shaped nuclei. The ethanolic extracts of the flowers showed more pronounced apoptotic effects on the cells. The mitochondrial membrane potential indicated that rhodamine 123 fluorescence signals suppressed mitochondrial potential due to the treatment with the extracts. Again, the apoptotic index of the ethanolic extract of the flowers remained the highest. Hence it can be concluded that the flower part of *N. paulsenii* Briq. was found to be the most active against the A459 human lung cancer cell line.

## 1. Introduction

The genus *Nepeta* belongs to the Lamiaceae family consisting of 300 species, which are widely distributed throughout different regions around the globe, especially Asia, Africa, America, and India. The attractive flowers of the genus *Nepeta* have a pleasant scent. Due to their diuretic, anti-asthma, antispasmodic, diaphoretic, and antitussive characteristics, various *Nepeta* species are used in traditional medicines [1,2,3,4,5,6,7]. It has been observed that cats become euphoric by the aromatic inflorescence of *Nepeta* genus plants [8]. Certain species of the *Nepeta* genus contain antioxidant characteristics and show antifungal, antiviral, and antibacterial activity [9,10]. *Nepeta paulsenii* Briq. has been scarcely studied for its biological potential [11]. Here, in this study, *Nepeta paulsenii* Briq. was used to study the extraction of phytochemicals from the root, stem, leaf, flower, and their mixture (whole plant) by using different solvents to evaluate their cytotoxic effect against lung tumor cells. Lung cancer may start in the cells present on the bronchi lining and other parts of the lung, i.e., bronchioles or alveoli. Lung cancer is found to be the second most occurring cancer in both women and men and is so far the principal cause of cancer death, killing more humans than colorectal, prostate, and breast cancers combined [12,13]. As this cancer starts to develop, the cells start releasing chemicals that cause the propagation of new blood vessels in the vicinity. These vessels nourish the affected cells, which can further continue to grow and form tumors. Most of the time, lung cancer can be prevented, especially when caused by smoking or by exposure to radon or other environmental aspects [14]. However, in specific other cases, lung cancer may be found in people without any of the known risk factors of the disease. It is not yet clear if these cancerous cells can be prevented [15].

The marketed chemotherapeutic drugs to treat lung cancer are cisplatin, carboplatin, docetaxel, paclitaxel, and gemcitabine, as shown in Supplementary Materials Figure S1 [16]. Like many other chemotherapeutics, these drugs also have severe side effects depending on the cell type and concentration of the dose. There are a number of Pt complexes that are used for the adjuvant therapy of cancers [17]. The challenge is that cancer cells may become resistant to cisplatin, and carboplatin can cause allergic reactions in human biological systems [18]. Myelosuppression is the maximum frequent dose-limiting poisonousness dose associated with docetaxel [19]. Leukopenia and neutropenia represent the major dose-limiting toxicity of paclitaxel [20], and nausea and vomiting were associated with gemcitabine [21].

**Figure 1:**
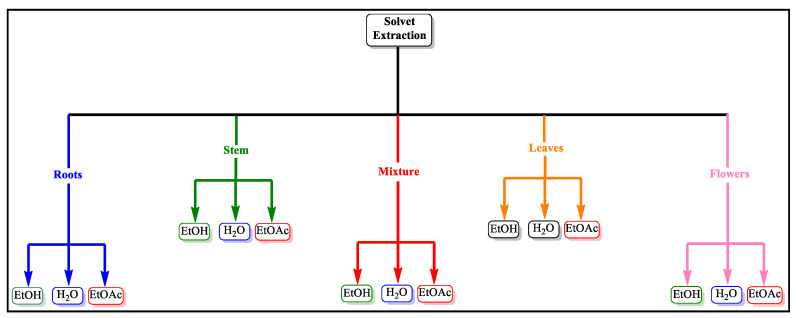
A schematic representation of solvent extraction using ethanol (EtOH), water (H_2_O), and ethyl acetate (EtOAc) for various plant sections and their mixtures (whole plant). A total of 15 various extracts were obtained.

An increase in the number of cancer cases around the globe has seen an increase in the subsequent resistance to chemotherapeutic agents and the awareness of using phytochemicals for the treatment and prevention of disease. Medicinally important plants contain phytochemicals of vast biosynthetic capacity [4,22,23]. Many phytochemicals have been pharmacologically tested for their chemotherapeutic and chemopreventive potential. Bioactive compounds derived from medicinal plants have proven to be a valuable source of drugs [23,24,25,26,27,28]. In Pakistan, *N. paulsenii* Briq. is used as herbal plant by the people in the northern areas, where its tea is served to the patients as ayurvedic medicine for the treatment of various diseases. Hence, the current study was carried out to study the extraction of phytochemicals from the stem, root, leaf, flower, and their mixture by using different solvent systems to then evaluate their cytotoxicity effect against the lung cancer cell line A549, as plotted in Figure 1.

## 2. Results and Discussion

The plant material was air-dried in a room that was protected against direct sunlight and dust. The plant parts, i.e., leaves, flowers, stems, and roots, were separated by hand, wearing gloves. All four samples were ground separately in a hammer or cutting mill Kinematica™ Polymix™ PX-MFC 90 D, with sieves in different mesh sizes from 0.2 mm–6 mm and speed regulation from 50–6000 rpm. This cutting mill is available at the Punjab Bioenergy Institute, University of Agriculture, Faisalabad, Pakistan. The powdered samples of leaf, flower, stem, and root were separately stored in zip-lock polythene bags at room temperature for further processing.

### 2.1. FT-IR Analysis of Extracts

Each of the extracts was evaporated using a rotary evaporator, and the residue was preserved for characterization and biological assays. Each of the fractions was characterized by FT-IR and GC-MS. The FT-IR spectra of ethanolic extract of each plant section majorly indicated the presence of alkyl groups, with sharp and intense vibrational bands, which was also supported by the presence of 1380–1400 cm^−1^ bending vibrations for –CH_2_– groups [29]. Furthermore, the appearance of vibrational bands in the range 1600–1700 cm^−1^ in the ethanolic extract of the leaves, roots, and stem indicated the possible presence of aromatic or unsaturated alkanes, as well as possible carbonyl groups. Such vibrations were not observed in the ethanolic extract of the flowers and whole plant (mixture). It can be expected that the chemical constituents in the ethanolic extracts might possibly be the long alkyl chains (saturated or unsaturated) and ketones. The unsaturated carbon-carbon double bonds were also indicated due to the appearance of vibrational bands between 3000–3100 cm^−1^ (Supplementary Materials Figure S2). Such vibrational bands remained suppressed in the FT-IR spectrum of the mixture (whole plant) due to the presence of several other groups, which may possibly be hydroxyls, carboxylics, and water as well. Similarly, the FT-IR spectra of the ethyl acetate extracts of each plant segment showed alkyl group vibrational bands in the region of 2800–2950 cm^−1^, as well as carbonyl groups in the range 1700–1750 cm^−1^ (in general). However, additional vibrational bands for benzene rings or carbon-carbon unsaturation were observed at 3000 and 3025 cm^−1^ in the roots, flowers, and whole plant spectrum only. The FT-IR spectra of the leaves, stem, and roots also indicated vibrational bands for –CH_2_– bending in the range 1390–1480 cm^−1^. Some broad regions were also found in the region of 3200–3500 cm^−1^, representing the presence of H_2_O traces. No aromatic region bands were found for the ethyl acetate extracts of the stems and leaves. On the basis of the principle “like dissolves like”, it may be concluded that CH_3_COOC_2_H_5_ may have extracted similar group chemicals from the plant. Supplementary Materials Figure S3 shows the FT-IR spectrum of the ethyl acetate extract of the flowers, which clearly indicates the presence of more than one type of carbonyls, showing peaks in the range of 1725–1750 cm^−1^, a small peak of C–H aromatic stretch at 3021 cm^−1^, and two vibrational string bands in the range of 2800–2950 cm^−1^ for C–H aliphatic stretching, indicating the presence of aromatic and aliphatic groups in the extract.

The FT-IR spectra of the water extract for each section were also found to be equally interesting, with some exceptions. For example, the FT-IR spectra of the root, stem, and mixture showed vibrational bands at 1580, 1600, 1640, and 1650 cm^−1^ for C=C aromatic and nonaromatic stretching in the extracted compounds. Only the FT-IR spectra of the root and stem showed a broad vibrational band in the range of 3100–3500 cm^−1^ for the presence of water, whereas, interestingly, all the others appeared free of water. The FT-IR spectra of the flowers remained the most interesting, which showed vibration bands at 2850 and 2920 cm^−1^ for C-H aliphatic stretch, two strong vibrational bands at 1700 and 1740 cm^−1^ for carbonyl groups, and a strong vibrational band at 1480 cm^−1^ for –CH_2_– bending (Supplementary Materials Figure S4).

### 2.2. GC-MS Analysis of Extracts

#### 2.2.1. GC-MS Study of Ethyl Acetate and the Ethanolic and Aqueous Extract of the Root

**Figure 2:**
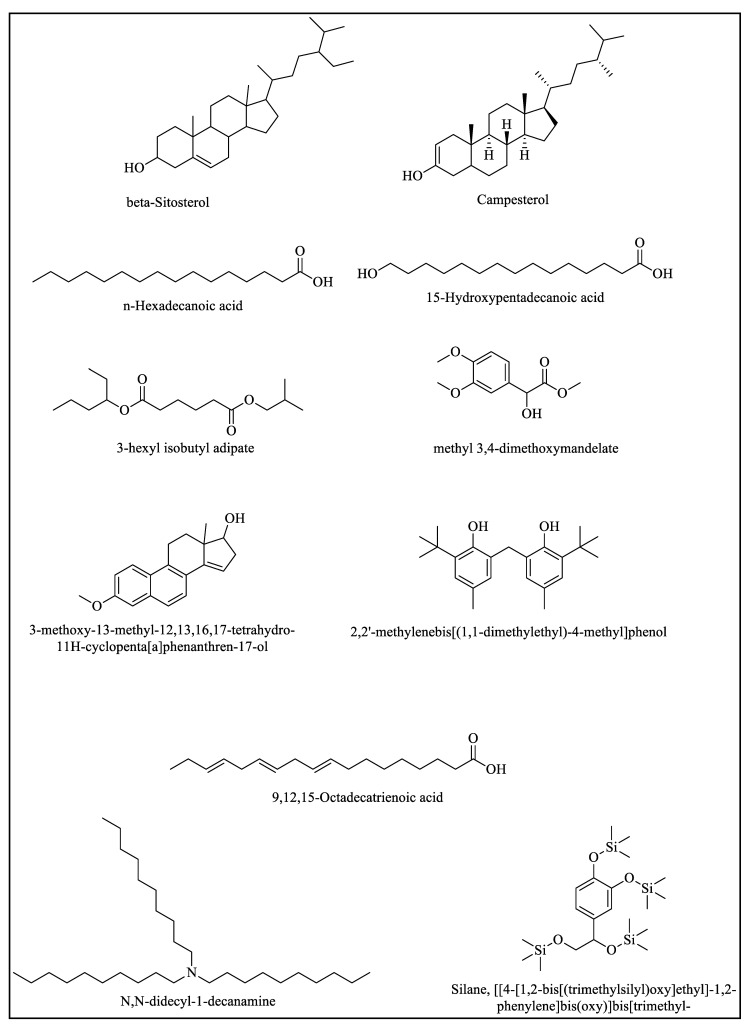
Chemical species identified by GC-MS in the CH_3_COOC_2_H_5_, aqueous, and ethanolic extracts of the stem, root, leaf, and flower at various retention times.

The GC-MS study of the ethyl acetate, ethanolic, and aqueous extracts of the root showed the existence of a number of phytochemical compounds, and the identified chemical compounds are shown in Supplementary Materials Table S1. Based on the peak area and retention period, the identification of the phytochemical compounds was established according to the reported method [30]. The identified compounds, with their retention time (RT) and peak area percentage (percent), are also expressed in Table S1. Using the National Institute of Standard and Technology 11 source library, component identification was carried out [31]. The main chemical components identified for ethyl acetate were campesterol and beta-sitosterol. The mass spectrum of the main compounds is shown in Supplementary Materials Figures S5–S8, and their chemical structures are also shown in Figure 2. The main chemical components identified for the aqueous extract were γ-sitosterol, beta-sitosterol, and n-hexadecanoic acid. The mass spectrum of the main compounds is shown in Supplementary Materials Figures S9–S13, and their chemical structures are also shown in Figure 2. The main chemical components identified in the ethanolic extract were beta-sitosterol, campesterol, and adipic acid. The mass spectrum of the main compounds is shown in Supplementary Materials Figures S14–S17, and their chemical structures are also shown in Figure 2.

There are several studies that have proven the cytotoxic effect of phytosterols, especially beta-sitosterol, against a number of cell lines [32,33,34]. Phytosterols have shown protective effects against colon, prostate, and breast cancer [35]. Hence, the presence of beta-sitosterol in *N. paulsenii* Briq. can make it a potent cytotoxic agent against tested cell lines. Campesterol is another phytosterol that has shown anti-angiogenesis and anticancer activity [36,37,38]. However, there are rare examples where alkanoic acids (e.g., pentanoic acid, hexanoic acid, etc.) and adipic acids have shown anticancer potential [39,40,41].

#### 2.2.2. GC-MS Study of the Ethyl Acetate, Aqueous, and Ethanolic Extracts of the Stem

The GC-MS study of the CH_3_COOC_2_H_5_, aqueous, and ethanol extracts of the stem showed the presence of 14, 11, and 20 phytochemical compounds, respectively, and the identified chemical compounds of the stem are shown in Table S2. In addition to the presence of beta-sitosterol and campesterol as the main chemical components identified in the ethyl acetate extract, 15-Hydroxypentadecanoic acid was found. The mass spectrums of the main compounds are shown in Supplementary Materials Figures S18–S21, and the chemical structure of 15-Hydroxypentadecanoic acid is shown in Figure 2. 15-Hydroxypentadecanoic acid is a fatty acid and has shown cytotoxic activities in some essential oils and plant extracts [42,43,44]. The main chemical components identified in the aqueous extract were also gamma- and beta-sitosterols, as well as methyl 3,4-dimethoxymandelate. Mendalic acid is well known for its organotin complexes and their applications in catalysis and medicine [45,46]. However, mandelic acid, 3,4-dimethoxy-, methyl ester has already been identified in the plant extracts of a few species tested for their antioxidant and cytotoxicity effects [47,48]. The mass spectra of the main compounds are shown in Supplementary Materials Figures S27–S30. The main chemical components identified for the ethanolic extract were, again, gamma and beta-sitosterols, campesterol, methyl 3,4-dimethoxymandelate, 3-methoxy-13-methyl-12,13,16,17-tetrahydro-11H-cyclopenta[a]phenanthren-17-ol, and 3-methoxy-13-methyl. The mass spectrum of the main compounds is shown in Supplementary Materials Figures S22–S26, and their chemical structures are also shown in Figure 2.

#### 2.2.3. GC-MS Study of the Ethyl Acetate, Ethanolic, and Aqueous Extracts of the Leaf

The GC-MS study of the ethyl acetate, ethanolic, and aqueous extracts of the leaf indicated the presence of 19, 14, and 12 phytochemical compounds, respectively. Based on the peak area and retention period, the identification of the phytochemical compounds was established [49]. The identified compounds, with their retention time (RT) and peak area percentage (percent), are also expressed in Supplementary Materials Table S3. Using the National Institute of Standard and Technology 11 source library, component identification was carried out [49]. The main chemical components identified for the ethyl acetate extract were 3-methoxy-13-methyl-12,13,16,17-tetrahydro-11H-cyclopentaphenanthren-17-ol and gamma- and beta-sitosterol. 3-methoxy-13-methyl-12,13,16,17-tetrahydro-11H-cyclopentaphenanthren-17-ol belongs to the class of sterols and has anticancer activity [50]. The mass spectra of the main compounds are shown in Supplementary Materials Figures S31–S33, and their chemical structures are shown in Figure 2. The main chemical components identified in the ethanolic extract of the leaf were gamma-sitosterol, beta-sitosterol, and 3-methoxy-13-methyl-12,13,16,17-tetrahydro-11H-cyclopentaphenanthren-17-ol. The mass spectra of the main compounds are shown in Supplementary Materials Figures S34–S36, and their chemical structures are shown in Figure 2. The main chemical components identified in the aqueous extract of the leaves were gamma- and beta-sitosterol, 3-methoxy-13-methyl-12,13,16,17-tetrahydro-11H-cyclopentaphenanthren-17-ol, 9,12,15-Octadecatrien-oic acid, and phenol 2 2′-methylenebis 6-(1 1-dimethylethyl)-4-methyl. The mass spectra of the main compounds are shown in Supplementary Materials Figures S37–S41, and their chemical structures are also shown in Figure 2. Octadecatrienoic acid is one of the common phytochemicals which is often found in medicinal plants [51,52,53,54] and has a significant role against cancer cell lines.

#### 2.2.4. GC-MS Study of the Ethyl Acetate, Aqueous, and Ethanolic Extracts of the Flower

The GC-MS study of the ethyl acetate, aqueous, and ethanolic extracts of the flower exhibited the presence of 31, 26, and 21 phytochemicals. Based on the peak area and retention period, the identification of the phytochemicals was established [49]. The identified compounds, with their retention time (RT) and peak area percentage (percent), are also expressed in Supplementary Materials Table S4. The spectra of the identified compounds for the ethyl acetate are shown in Supplementary Materials, and their chemical structures are shown in Figure 2**.** The major chemical components found in the ethyl acetate extract of the flowers are beta-sitosterol, Urs-12-en-28-oic acid, 3-hydroxy-, methyl ester, Bis(2-ethylhexyl) phthalate, and isopropyl myristate. Urs-12-en-28-oic acid, 3-hydroxy-, methyl ester is one of the important bioactive compounds that has been recently identified in *Calotropis gigantea* leaves [55,56]. Bis(2-ethylhexyl) phthalate is another important bioactive chemical that can be isolated from various plant species [57]. Among them, isopropyl myristate is a potential anticancer and anti-inflammatory agent, which has been found in the roots of *Saussurea hypoleuca* spreng [58]. The main chemical compounds identified in the ethanolic extract of the flowers were beta-sitosterol, 9,12,15-Octadecatrienoic acid, and 9,12,15-Octadecatrien-1-ol (Table 1). 9,12,15-Octadecatrien-1-ol has been recently found in the methanolic extract of *Papaver decaisnei*, the extract of which is potentially anticancerous. The mass spectrum of the main compound is shown in Supplementary Materials Figures S48–S49 and their chemical structures are shown in Figure 2. The main chemical components identified in the aqueous extract of the flowers are 1-Decanamine, N,N-dodecyl-, Cyclodecasiloxane, eicosamethyl, Silane, [[4-[1,2-bis[(trimethylsilyl)oxy]ethyl]-1,2-phenylene] bis(oxy)]bis[trimethyl-, 3-methoxy-13-methyl-12,13,16,17-tetrahydro-11H-cyclopentaphen-anthren-17-ol, and phenol 2 2′-methylenebis 6-(1 1-dimethylethyl)-4-methyl. The mass spectrum of the main compounds is shown in Supplementary Materials Figures S50–S54 and their chemical structures are shown in Figure 2.

#### 2.2.5. GC-MS Study of the Ethyl Acetate, Aqueous, and Ethanolic Extracts of the Mix of the Flowers, Stem, Leaves, and Root

The chemical composition of the ethyl acetate, aqueous, and ethanolic extracts of the mix of the flowers, stem, leaves, and root fractions and subfractions was studied using fragment arrangement in the GC-MS mass spectrum. The ethyl acetate, aqueous, and ethanolic extracts of the mix of flowers, stem, leaves, and root in GC-MS showed 19, 20, and 26 peaks, respectively, indicating the presence of various phytochemicals, as shown in Table S5. Upon the evaluation of the mass spectra of the compounds using the main library, all these constituents were probably identified and characterized [59]. The spectra of the identified compounds for the ethyl acetate extract are shown in Supplementary Materials Figures S55–S57 and their chemical structures are shown in Figure 2. The spectra of the identified compounds in the ethanolic extract are shown in Supplementary Materials Figures S58–S61 and their chemical structures are also shown in Figure 2. The spectra of the identified compounds in the aqueous extract are shown in Supplementary Materials Figures S62–S70 and their chemical structures are also shown in Figure 2.

## 3. Cytotoxicity Potential of *Nepeta paulsenii* Briq.

### 3.1. Cytotoxicity Effect of the Ethyl Acetate Extract of the Flowers, Leaf, and Stem

**Figure 3:**
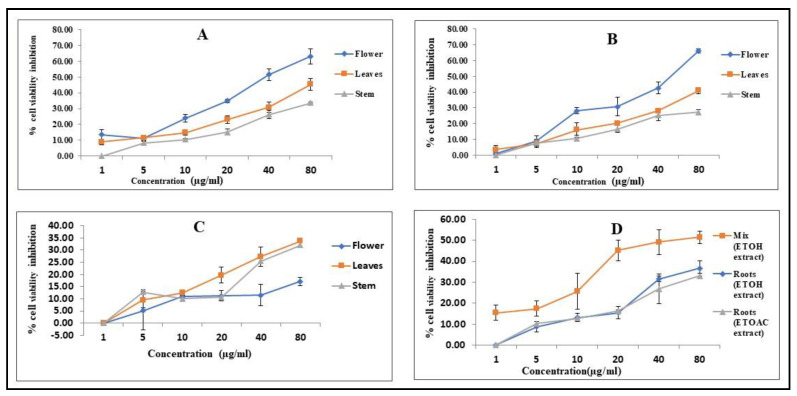
Dosedependent cytotoxicity effects of stem, leaf, and flower on A549 cell line. (**A**) Ethyl acetate extract. (**B**) Ethanolic extract. (**C**) Aqueous extract. (**D**) Ethanolic and ethylacetate extracts of roots and mix (leaves, stem, and flower).

**Figure 4:**
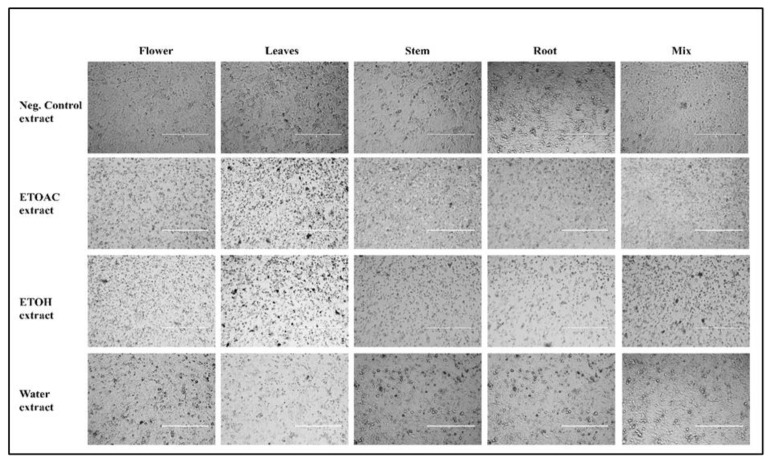
A549 cell photos were captured using a digital camera under an inverted contrast phase microscope, following treatment with the extracts.

Therefore, the current study was planned to investigate the cytotoxic potential of the CH_3_COOC_2_H_5_ extract of the flower, leaf, stem, and root against the cancer cell line A549. It can be seen from Table 1 that all the ethyl acetate extracts have IC_50_ values in the range of 51.57–113.80 μg/mL. Among all the CH_3_COOC_2_H_5_ extracts, the CH_3_COOC_2_H_5_ extract of the flower showed more cytotoxicity, with an IC_50_ of 51.57 µg/mL. The ethyl acetate extract of the stem was found to be less cytotoxic, as the IC_50_ value was higher than the rest of the ethyl acetate extracts (Table 1), whereas the ethyl acetate extract of the flower showed a considerably low IC_50_. A comparison of the IC_50_ values between the ethyl acetate extract of the flower and the b standard drug, 5-fluorouracil (5-Fu), showed that the ethyl acetate extract of the flower showed even better cytotoxic results than 5-Fu against the A549 cell line. This activity could be attributed to the presence of Bis(2-ethylhexyl) phthalate (DEHP). There are a number of epidemiological and rodent studies that prove DEHP has cytotoxic effects, but the clinical studies are not sufficient enough to support this argument [60]. Moreover, it is still unclear which major pathways are involved in DEHP-induced tumorigenesis [60]. The inhibition percentage of cell propagation was assessed at different concentration levels of the ethyl acetate extract of leaf, stem, and flower against cell line A549. It is clear from these graphs that the cytotoxicity of the ethyl acetate extract of the flowers, leaves, and stem increased with an increase in concentration, which proved that the extracts have a dose-dependent cytotoxicity effect; the dose-dependent graphs are shown in Figure 3A. The photo-micrographic images revealed that the ethyl acetate extract of the flower affected the morphology of the cancer cells, with a better assessment of cell viability when compared to the other extracts, as shown in Figure 4. Ethyl acetate extracts of various plant species have already been studied against different cell lines, e.g., human cervical cancer [61,62], breast cancer [63,64], and skin cancer [65]. The results have been significant against these cancer cell lines; however, we could not find a test for the ethyl acetate extract against any other cell line in the past five years.

### 3.2. Cytotoxicity Effect of the Ethanolic Extract of the Flower, Leaf, and Stem

The ethanolic extract of the flowers, leaves, and stem was studied to check its cytotoxicity against the A549 cancer cell line. The cytotoxicity potential of the ethanolic extract of the flower, leaves, and stem was evaluated using an MTT assay on the A549 cancer cell line. The percentage inhibition of cell propagation was assessed at different concentration levels of the ethanolic extract of the leaves, stem, and flowers against the A549 cell line. It is clear from these graphs that the cytotoxicity of the ethanolic extract of the flowers, leaves, and stem increased with an increase in concentration, which proved that the extracts have a dose-dependent cytotoxicity effect, and the dose-dependent graphs are shown in Figure 3B. The ethanolic extract of the flowers, leaves, and stem showed cytotoxicity potential in a concentration-dependent manner and showed an IC_50_ in the range of 50.58–2691.76 μg/mL. Table 1 shows cytotoxicity activity against the IC_50_ of the ethanolic extract of the flowers, leaves, and stem, and a comparison between the IC_50_ values of the ethanolic extract of the flowers and the standard drug, 5-fluorouracil (5-Fu), showed that the ethanolic extract of the flower had cytotoxic results that were even better than 5-Fu against the A549 cell line. The ethanolic extract of the stem was found to be less cytotoxic, as the IC_50_ value was higher than the rest of the ethanolic extract (Table 1), whereas the ethanolic extract of the flowers showed a considerably lower IC_50_, which could be attributed to the presence of beta-sitosterol, which is one of the richest dietary phytosterols, which are the counterparts of cholesterol [66], and beta-sitosterol is one of the phytochemicals that have cancer-treatment properties (as reported in the previous studies) and also exhibits selective toxicity towards cancer cells without inducing significant toxicity to the normal cells [67,68,69,70]. As the existing anticancer drugs present few severe drawbacks, including toxic side effects, beta-sitosterol can be a competent alternative, as beta-sitosterol has several functional biochemical similarities with existing drugs, such as taxol and taccalonolides [71]. The photo-micrographs of the treated cells are presented in Figure 4. The ethanolic extracts of the plants tested most abundantly for biological potentials [72,73,74]; specifically, the ethanolic extracts of various *Nepeta* species have been frequently studied for their cytotoxicity [75,76,77,78]. The ethanolic extract of *Nepeta paulsenii* Briq. showed considerable cytotoxic effects against cancerous cell line A549; however, none of the extracts were found to be cytotoxic in normal cell lines.

### 3.3. Cytotoxicity Effect of Aqueous Extract of Leaves, Flowers and Stem

In the present work, the in vitro cytotoxicity of the aqueous extract of the flowers, stem, and leaves were determined against the A549 cell line based on an MTT assay, and all the aqueous extracts of the flowers, stem, and leaves showed activity against lung cancer (A549), and all the aqueous extracts of the flowers, leaves, and stem demonstrated cytotoxicity activity, with their different IC_50_ values tabulated in Table 1. The IC_50_ values of the aqueous extract of the flowers, stem, and leaves ranged from 1000–123.8 µg/mL. A comparison between the IC_50_ values of the aqueous extract of the flowers, stem, and leaves and the standard drug, 5-fluorouracil (5-Fu), showed that the aqueous extract of the flowers, stem, and leaves showed less cytotoxic results than 5-Fu against the A549 cell line.

The highest cytotoxicity activities were observed in the aqueous extract of the stem, with an IC_50_ value of 123.8 µg/mL; the lowest activity was observed in the aqueous extract of the flowers, with an IC_50_ value of >1000 µg/mL. The cytotoxicity action against the A549 cell line corresponds to the order: stem > leaves > flowers. As the aqueous extract of the stem showed better activity, this could be due to the presence of a derivative of mandalic acid. Mandelic acid is used in the treatment of skin problems and is an antibacterial agent, particularly against urinary tract infections [79,80,81]. Recently, derivatives of mandelic acids have also shown potent in vitro cytotoxicity activity against the HEK-293, MCF-7, PC-3, and HepG-2 cell lines [45]. A preliminary investigation of the cytotoxicity potential (as a function of inhibition of cell propagation at different concentrations of ethanolic extracts of flowers, leaves and stem ranging from 1 µg/mL to 80 µg/mL) was undertaken to test the A549 cell line. We found that there is a general increase in the percentage of cell death with an increase in the concentration of the aqueous extracts of the stem, leaves, and flowers. It is clear from these graphs that the cytotoxicity of the aqueous extract of the flowers, leaves, and stem increased with increases in concentration, proving that the extracts have a dose-dependent cytotoxicity effect, and the dose-dependent graphs are shown in Figure 3C. Moreover, cell images were taken under a microscope using inverted-phase contrast. The cells from the control group exhibited totally confluent growth, as shown in Figure 4. Treatment with the aqueous extract of the stem showed the marked inhibition of cell viability, whereas the aqueous extract of the flowers showed a mild cytotoxicity effect, as shown in Figure 4, and the cells exhibited clear marks of cytotoxicity caused by affected cellular morphology. The testing of water extracts from medicinal plants is uncommon in the scientific community; however, there are reports on the water extracts of various *Nepeta* species that have been tested for their biological potential [4,75,82], and the results remain comparable with the other types of extracts.

### 3.4. Cytotoxicity Activity of the Extracts of the Root, Stem, Leaf and Flower Mix against A549 Lung Cancer Cell Line

**Figure 5:**
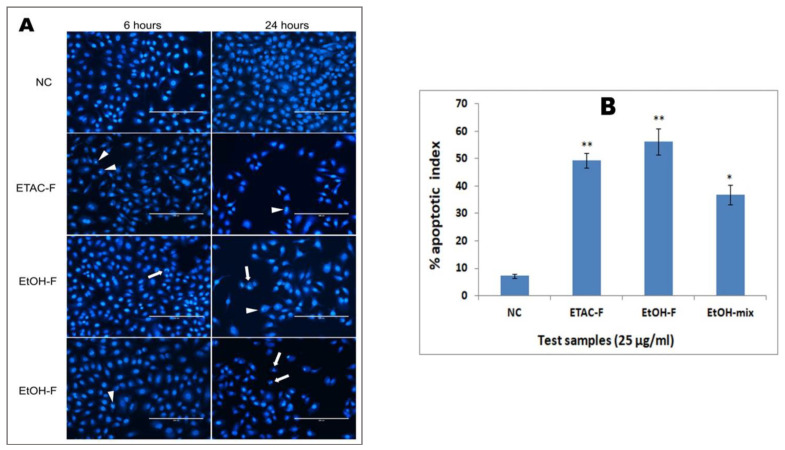
(**A**) These photo-micrographs illustrate the pictures of the A549 cells with Hoechst 33342 staining. The untreated cells (negative control) exhibited the cells growing in a lively manner, with clear nuclear and further cellular structures. The cells treated with the extracts showed clear characteristic apoptosis changes. The arrowheads indicate chromatin dissolution, breakdown, and fragmentation. These arrows specify the condensed, fragmented, and crescent-shaped nuclei. Among the extracts tested, EtOH-F (ethanol extract of flower) showed a more pronounced apoptotic effect on the cells. (**B**) Percentage representation of apoptotic indices for different tested groups. Index apoptotic for untreated (negative control) A549 cells was 7.2 ± 0.8%. In contrast, the apoptotic indices obtained for the treated cells with the ETAC-F, EtOH-F, and EtOH-mix extracts were 49.4 ± 2.7%, 56.2 ± 4.8%, and 36.9 ± 3.6%, respectively. NC = negative control; ETAC-F = ethylacetate extract of flower; EtOH-F = ethanol extract of flower; EtOH-mix = ethanol extract of the mixture. Values are presented as % mean ± SD (n = 6), * represents *p* < 0.05 and ** represents *p* < 0.01.

The phytochemicals in the mix extracts of the root, stem, leaves, and flowers were evaluated for their cytotoxic potential against lung cancer A549 cells using an MTT assay. Only the ethanolic extract showed cytotoxicity, with IC_50_ values in the range of 62.82 µg/mL; these IC_50_ values are tabulated in Table 1. A comparison between the IC_50_ values of the ethanolic extract of the mix (flowers, leaves, stem, and root) with the standard drug, 5-fluorouracil (5-Fu), showed that mix (flower, leaves, stem, and root) had cytotoxic potential that was even better than 5-Fu against the A549 cell line. The strong cytotoxic potential of the ethanolic extract of the mix (flower, leaves, stem, and root) was due to the presence of n-hexadecanoic acid [83,84,85]. The presence of this compound might be responsible for anticancer activity [86], and its better activity might be due to its lipophilic character that enables them to cross phospholipid bilayer membrane barriers, which ultimately alters the physiological function of the cell. On the other hand, the cytotoxicity potential against lung cancer A549 was not affected by the aqueous extract of the mix (flower, stem, and root) and the ethyl acetate extract of the mix (flower, stem, and root). The percentage inhibition of cell viability was assessed at several different concentration levels of the ethanolic extract of the mix (leaves, stem, root, and flowers) against cell line A549. It is clear from these graphs that the cytotoxicity of the ethanolic extract of the mix (leaves, stem, root, and flowers) increased with an increase in the concentration, which proved that the extracts have a dose-dependent cytotoxicity effect, and the dose-dependent graphs are shown in Figure 3D. Moreover, the cell images were taken under a microscope using inverted-phase contrast. The control group cells showed fully confluent growth, as shown in Figure 4. Treatment with the ethanolic extract of the mix (leaves, stem, root, and flowers) indicated a marked inhibition in cell viability, whereas the ethyl acetate and aqueous extracts of the mix (leaves, stem, and flowers) showed no cell viability, as shown in Figure 5, and the cells show specific marks of cytotoxicity.

### 3.5. Effect of Extracts on Nuclear Morphology and Condensation in A549 Cancer Cells

During apoptosis, the apoptotic cell undergoes a sequence of distinguishing morphological and cellular changes, such as nuclear condensation and fragmentation, suspension of chromatin material, and modifications in the cell membrane [87]. In order to investigate the specific typical features of apoptosis and to confirm the mechanism of cytotoxic action of the extracts on the cells, we conducted bioassays to identify the modifications in the mitochondria and nucleus of the cancer cells treated with the active extracts. There are only a few reports on the nuclear morphology of *Nepeta* species [88,89]. Hence, in this study, the lung cancer (A549) cells, which are the most sensitive cells, were chosen to investigate the cause of the cytotoxicity. The findings of the test revealed the typical morphological and apoptotic variations in the treated cells in a time-dependent manner (Figure 5A). However, the untreated cells demonstrated prominent intact cell membranes and nuclei without any major changes in cell morphology. On the other hand, the cells treated for 6 h displayed clear apoptotic induction. The nuclei of most of the cells started to condense and distributed the chromatin material irregularly in the cytoplasm (indicated by the arrowheads in Figure 5A). The cells also showed shrunken, crescent-shaped nuclei with compact chromatin, which are typical signs of the early stages of apoptosis (indicated by the arrows in Figure 5A). After 24 h of treatment, a reasonable number of cells had distinct chromatin structures that are a hallmark of karyorrhexis, a later stage of apoptosis. The apoptotic index was estimated after 24 h of treatment. The apoptotic index (Figure 5B) for the untreated A549 cells was 7.2 ± 0.8%. Whereas the apoptotic index obtained for the treated cells with the ETAC-F, EtOH-F, and EtOH-mix extracts were 49.4 ± 2.7%, 56.2 ± 4.8%, and 36.9 ± 3.6%, respectively (Figure 5B).

### 3.6. Effect of the Extracts on Mitochondrial Membrane Potential in Human Lung Cancer (A549) Cells

**Figure 6:**
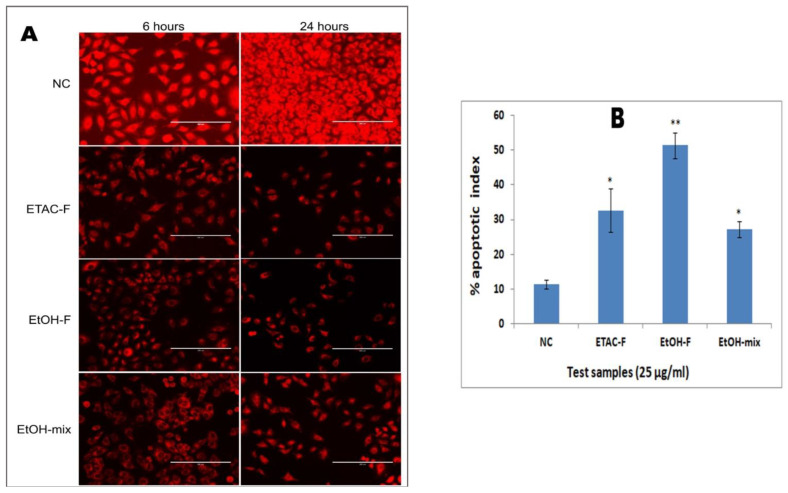
(**A**) The photo-micrographs call attention to the capability of the extracts to interrupt the potential of the mitochondrial membrane. (**B**) Graphical illustration of percentage apoptotic indices. The apoptotic index for every test group was expressed as the percentage of the ratio of the unstained cell no./overall cell no. in 10 different fields. NC (7.2 ± 0.8%) = negative control; ETAC-F (49.4 ± 2.7%) = ethyl-acetate extract of flower; EtOH-F (56.2 ± 4.8%) = ethanol extract of flower; EtOH-mix (36.9 ± 3.6%) = ethanol extract of mixture. Values presented as percentage mean ± standard deviation (n = 6), ** represents *p* < 0.01 and * represents *p* < 0.05.

Rhodamine 123 is a cationic probe that can be absorbed readily by live cells and gets accumulated into the mitochondria [90]. When the mitochondrial membrane potential is lost, it is considered an indication of early apoptotic events, which avoids any entry of the cationic probe, rhodamine 123, into the mitochondria [91]. In order to further investigate the induction of apoptosis by the extracts in the A549 cells, the loss of mitochondrial integrity is observed, and for that purpose, the rhodamine assay was performed. According to this assay, the cells were exposed to rhodamine 123 dye, and its intensity in the cells was measured (Figure 6A). The decrease in the potential of the mitochondrial membrane was associated with a decrease in rhodamine 123 uptake by the cells, and subsequently, the florescent signals were reduced accordingly. The findings of the current study demonstrated high fluorescence intensity in the untreated cells, indicating the presence of a number of living cells in the group. Whereas the significantly fluorescent signal was reduced in the cells treated with the extracts, this suggests a loss in mitochondrial membrane potential in the treated cells. Furthermore, as the treatment duration increased, the luminous intensity dropped (Figure 6A). The apoptotic index determined after 24 h for the negative control group was 11.4 ± 1.3%. The apoptotic indices after 24 h of treatment with the ETAC-F, EtOH-F, and EtOH mix extracts were 32.66 ± 6.3%, 51.4 ± 3.7%, and 27.2 ± 2.2%, respectively (Figure 6B). A notable reduction in the potential of the mitochondrial membrane of the A549 cell lines may possibly be due to the induction of apoptosis affected by the treatment with the extracts.

The photo-micrographs of the cells treated with Hoechst 33342 and rhodamine 123 staining indicated that the cytotoxicity induced by the extracts could possibly be due to apoptosis. The affected cells revealed the unique characteristics of apoptosis, i.e., nuclear condensation, apoptotic bodies, and membrane blebbing present in the cell cytoplasm. A significant number of cells also revealed crescent-shaped nuclei, which indicated the advanced apoptosis stage.

## 4. Experimental Section

### 4.1. Material and Methods

#### 4.1.1. Plant Material

*Nepeta paulsenii* Briq. plant material was gathered from Skardu, Pakistan, in August 2018 during the flowering season. The taxonomic identification was made in July 2018. The voucher specimen was recognized by Dr. Mansoor Hameed (Chairman) at the Department of Botany, University of Agriculture, Faisalabad, Pakistan, and it was deposited at the Herbarium present in the Botany department, University of Agriculture, Faisalabad, Pakistan.

#### 4.1.2. Plants Extracts

Solvents used for extraction were ethanol, ethyl-acetate, and water. A 15 g powdered material of leaves, roots, flowers, and stems was separately subjected to extraction using Soxhlet apparatus. After that, all the extracts were stored in separate bottles with proper labeling. Later, these extracts were subjected to an EV311H rotary evaporator to completely dry the solvent and obtain the dried form of the plant extracts.

#### 4.1.3. Characterization of Plant Extracts

Each of the extracts of the dried plant material was subjected to the FT-IR (FTIR spectrophotometer operating OPUS programming with a range of 4000–400 cm^−1^, Bruker Alpha optic) to assess the possible functional groups in the extracted material. ATR (attenuated total reflection) method was applied for sample testing, whereas no pallet formation was required with KBR in this method, and the sample was placed over the diamond surface and the reflection was recorded. Furthermore, each of the samples was subjected to Agilent Technologies, GC-MS mass spectrum, 7890b for GC system and 5977A for MS system to assess the possible identification of the chemical constituents so that the biological significance could be related to the chemical constituents present in the plant extracts. Each of the extracts was dissolved in the respective solvents and added into an Agilent 7890A GC system coupled with an MS (technologies of Agilent). The conditions set for the GC–MS analysis were as reported earlier [92], and the NIST14.L library (2018) was then investigated to relate the structures of the complexes with the NIST database (C:\Database\NIST11.L) [92].

#### 4.1.4. Cell Culture

A549 cell lines for human lung cancer were obtained from the American Type Culture Collection (ATCC). Cells were grown in Dulbecco’s Modified Eagle Medium (DMEM), GIBCO^TM^, including 10% FBS, 100 units per milliliter penicillin, and 100 μg per milliliter streptomycin at 37 °C in an incubator containing 5 % carbon dioxide.

#### 4.1.5. MTT Assay (Colorimetric Assay)

The MTT assay was executed, as described in [93], with the treatment of serial concentrations of each *Nepeta paulsenii* Briq. extracts (1, 5, 10, 20, 40, and 80 μg/mL) were added and then incubated for 3 days under humified 5% CO_2_ environment. Finally, the MTT dye (2 μL) was added to a 4.14 mg/mL concentration solution (to each well) and incubated for 4 h at 37 °C. After the removal of the medium, formazan was dissolved in DMSO, and the absorbance was measured at 590 nm using microplate reader. The growth inhibition was detected using Growth inhibition = (control O.D − sample O.D.)/control O.D.

##### Cell Viability Assay

Cells were planted on 24-well plates (used in tissue culturing), and the *N. paulsenii* extract was added to a series of concentrations, as indicated. The cultures were preserved at 37 °C in incubators containing 5% CO_2_ for three days. The cells were gathered using trypsinization and then stained using trypan blue. The total cells, including dead cells and the overall cells, were counted. The percentage of viable cells (%) was calculated as [(total cells-dead cells)/total cells] × 100% [93]. The negative control was free of any extract concentration.

##### Cytotoxicity Assay

The cytotoxic activity of *Nepeta paulsenii* was examined using a cytotoxicity detection kit (Roche Molecular Biochemicals), which was related to the determination of lactate dehydrogenase (LDH), which was released from the dead cells in response to cytotoxicity. For the cultures without cells, the supernatants were taken from *N. paulsenii* treated with the A549 cells and were then collected and shifted in the microtiter plates. The mixture of substrate-possessing tetrazolium salts was added and then inoculated for 3 h. Formazan dye was then used to quantify via the measurement of absorbance at 490 nanometer utilizing the ELISA microplate reader (Ascent Multiskan) [94].

#### 4.1.6. Hoechst 33342 Stain Assay

The effect of the plant extract on the condensation of nuclear chromatin in lung cancer cells (A549) was calculated using fluorescence microscopy by Hoechst (33342) staining [95]. Cells were used, along with the extracts (25 µg/mL), and determined in isolation at two different time periods (6 and 24 h). Cells left untreated were used as negative (-) control. Cells were brought to motionless in 4% paraformaldehyde for about 20 min, just before staining using Hoechst 33342 stain [1 mgmL^−1^ in phosphate buffer solution (PBS)] for 20 min. The shrinkage of the cytoplasm and the condensation of the nucleus were observed with the fluorescent microscope. Cells with brightly colored (either fragmented or condensed) nuclei were advised to be apoptotic. The no. of cells that were morphologically apoptotic was counted randomly using microscopic fields in each well. The photography of the cells was carried out at 20 × magnification by EVOS fluorescent digital microscope (Advanced Microscopy Group, Waltham, MAUSA). After 24 h of treatment, the index of apoptosis was measured, i.e., % of apoptosis nuclei in comparison with the total no. of cells, and then calculated as mean 6 ± SD (n = 8).

#### 4.1.7. Stain of Rhodamine 123 Assay

Detection of the variations in the potential of mitochondrial membrane in the A549 cells (after the treatment with the extract) was evaluated by the retention of rhodamine 123 by following the method reported by Abe and coworkers [96]. A549 cells were then plated in 6-well plates overnight. The cells were subjected to treatment among the various extracts at 25 µg/mL for about 6 and 24 h and were then made static using 4 % of paraformaldehyde for about 20 min. Untreated cells were thought to be negative (-) control. The rhodamine 123 stain was applied to the cells at its final conc. of 5 mgmL^−1^ and inoculated for about 30 min to stain mitochondria. The wells were then photographed by the use of EVOS fluorescent digital microscope using 20 × magnification to check the fluorescent signals. The index of apoptosis for every treatment set was detected by counting the cells having apoptosis (the unstained cells: the total cell no.) in the microscopic fields, which were selected randomly.

### 4.2. Statistical Analysis

All the assays were carried out in final, and the triplicate data were reported as mean standard deviation. Various concentrations of the plant extracts were evaluated, and then half-maximal (IC_50_) inhibitory concentration values for all of the experiments were calculated by linear regression analysis (LRA) [97]. Statistical difference of mechanistic studies (Hoechst 33342 Stain and Rhodamin 123 Stain) was analyzed by ANOVA (one-way analysis of variance), and was followed by Tukey’s multiple tests. Differences were deliberated significant at *p* < 0.05 and *p* < 0.01.

## 5. Conclusions

The ethanolic and ethyl acetate extract of the flower exhibited the highest cytotoxic effect among all the other extracts of *N. paulsenii* Briq., and this was even better than 5-FU (the standard drug) against the A549 cell line. Furthermore, the flower extracts also induced apoptotic death, which was observed through Hoechst 33342 Stain and Rhodamin 123 Stain assays. Hence, the flower section of *N. paulsenii* Briq. remains the most active component for the treatment of lung cancer.

## 6. Recommendations for Future Work

The current study suggests that, in the future, the bioassay-guided isolation of the flower section of the studied plant needs to be performed to isolate the chemical moiety or moieties responsible for the inhibition of lung cancer cells (A549).

## Figures and Tables

**Table 1:** IC_50_ values of the extracts of different segments and the mixture of *N. paulsenii* Briq. on lung cancer cell line A549.

	IC_50_ Values (µg/mL)				
	Flower	Leaves	Stem	Root	Mix
ETOAc extract	51.57	86.25	113.80	>1000	ND
ETOH extract	50.58	95.01	>1000	559.88	62.82
H_2_O extract	>1000	847.8	123.8	ND	ND
5-FU	83.62				

## Data Availability

The data supporting the findings of current research work are available in the article and Supplementary Materials.

## Supplementary Materials

The following supporting information can be downloaded at: https://www.mdpi.com/article/10.3390/molecules28062812/s1, Supplementary Materials Figures S1–S70, Supplementary Materials Tables S1–S5.

## Sample Availability

Samples of the compound are not available from the authors.
