# Supplementary material for: Cytotoxicity against A549 Human Lung Cancer Cell Line via the Mitochondrial Membrane Potential and Nuclear Condensation Effects of Nepeta paulsenii Briq., a Perennial Herb
Source: Molecules. 2023 Mar 20;28(6):2812. doi: 10.3390/molecules28062812 (PMC10054104; doi:10.3390/molecules28062812)
Supplement: Supplementary file 1 [file molecules-28-02812-s001.zip › molecules-2109658-supplementary.pdf]

SUPPLEMENTARY FILE

Human Lung Cancer Targeted Cytotoxicity, Mitochondrial Membrane Potential and Nuclear Condensation effects by *Nepeta paulsenii* Briq., A Perennial Herb

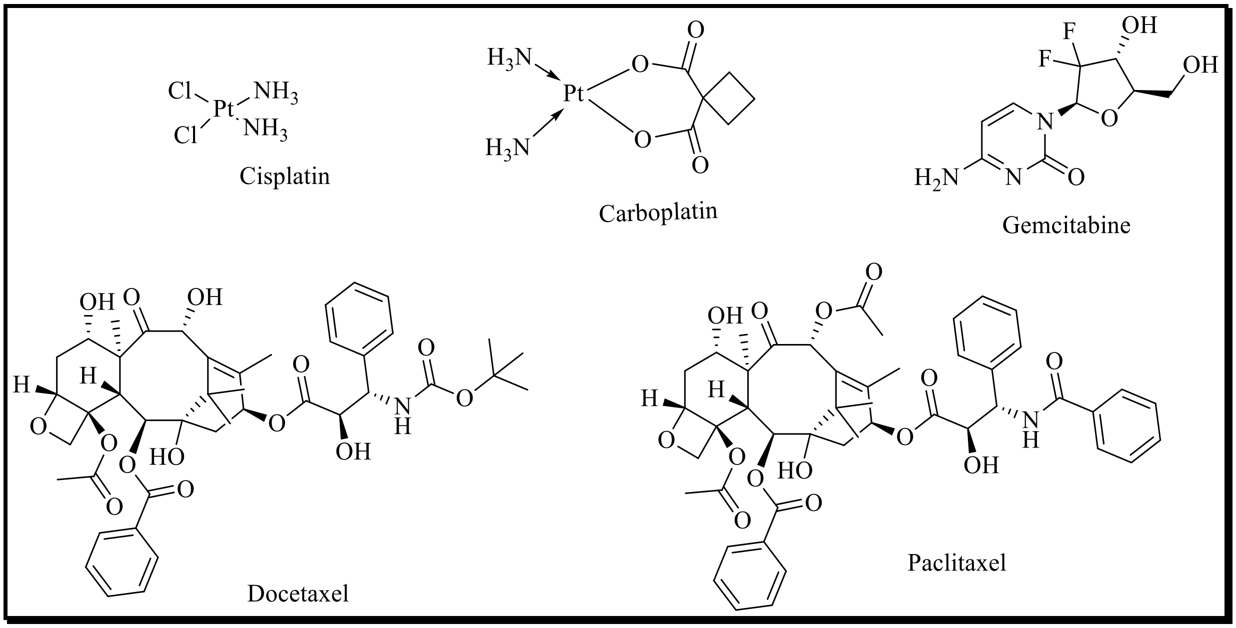

Figure S1: Marketing drugs for the treatment of cancer related to lung cancer

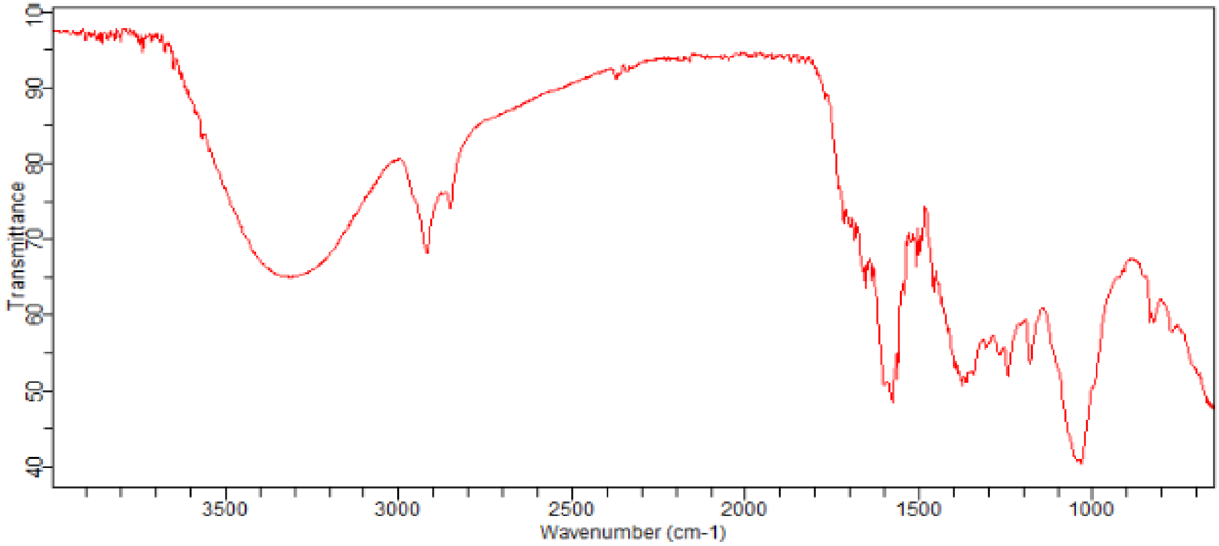

Figure S2: Shows the FT-IR spectrum of ethanolic extract of *Nepeta Paulsenii* (stem section).

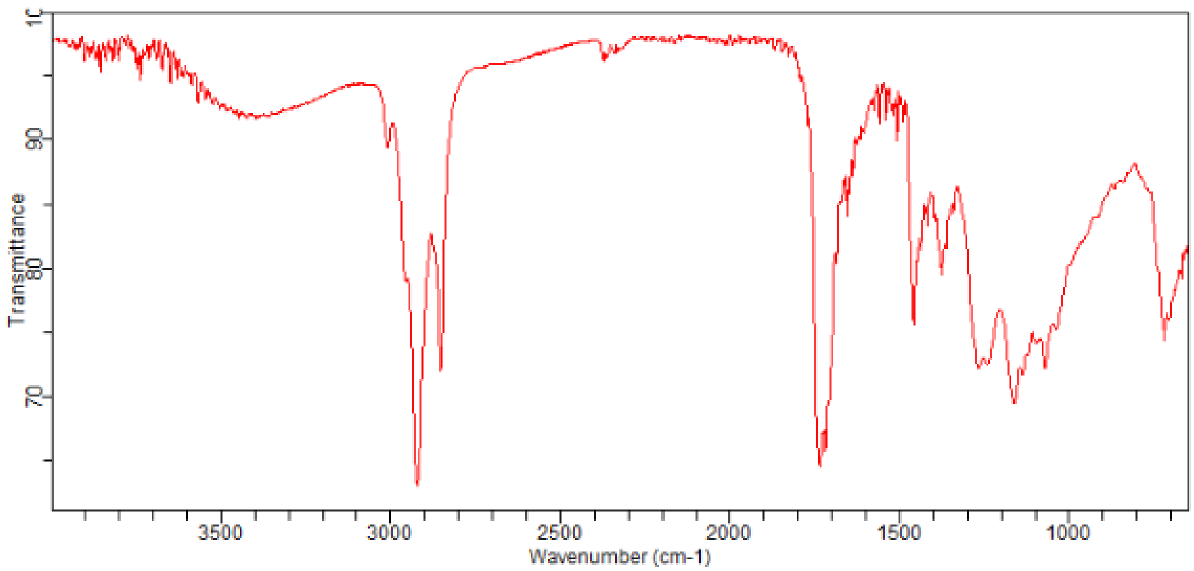

Figure S3: Shows a representative FT-IR spectrum of ethyl acetate extract of flowers.

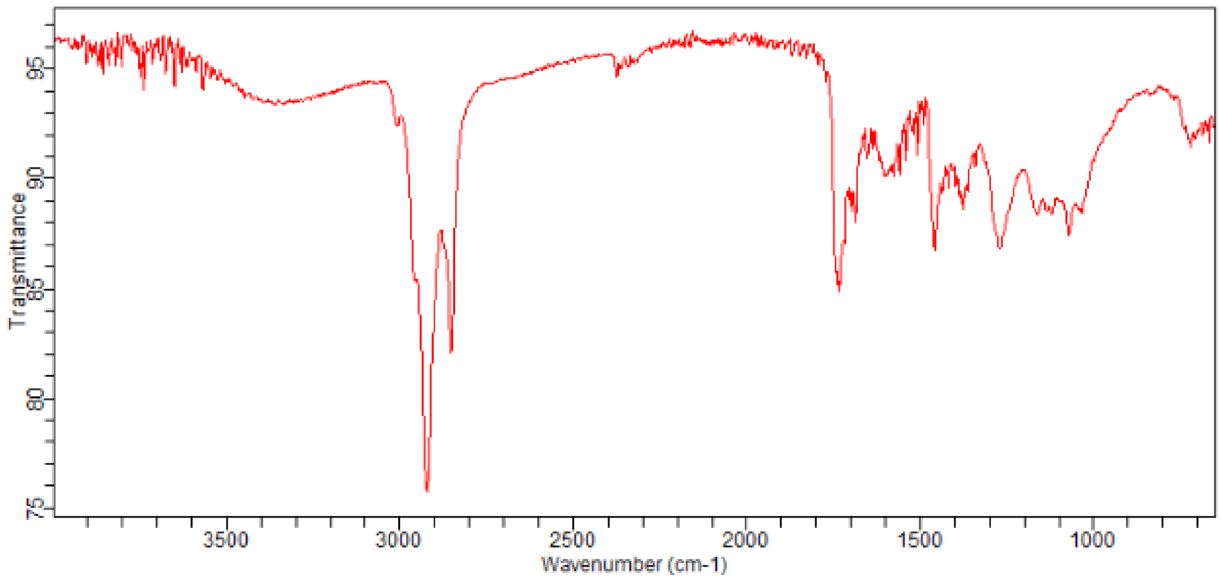

Figure S4: Shows FT-IR spectrum of water extract of flowers.

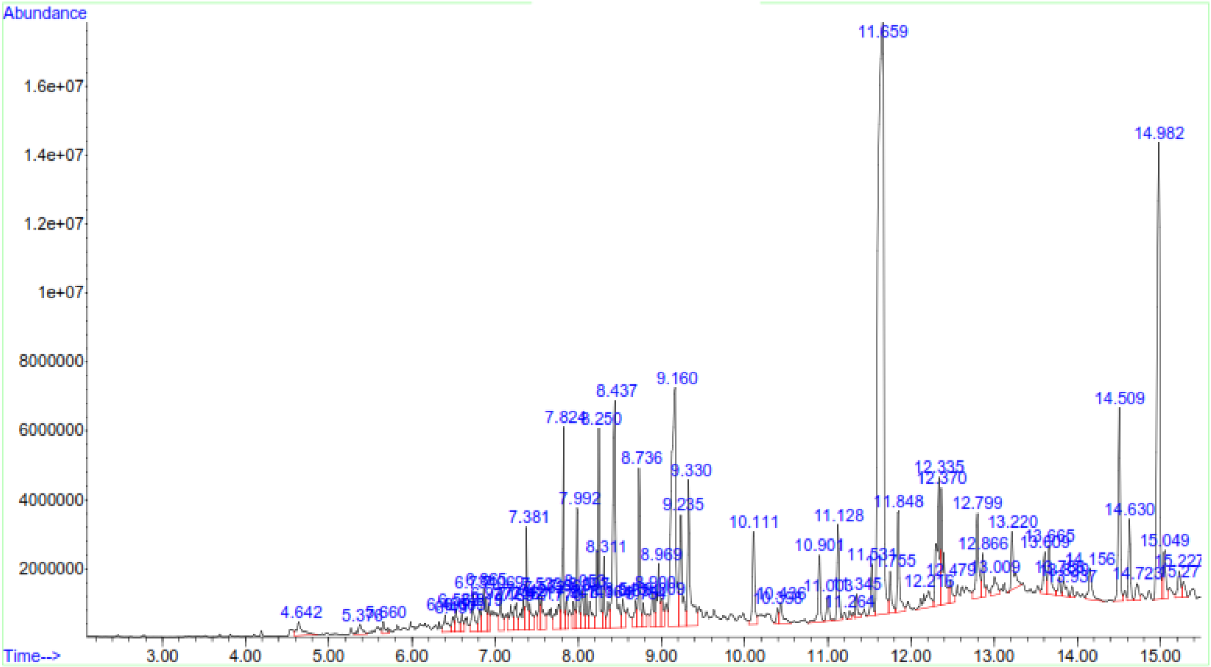

Figure S5: GC spectrum of ethyl acetate extract of root

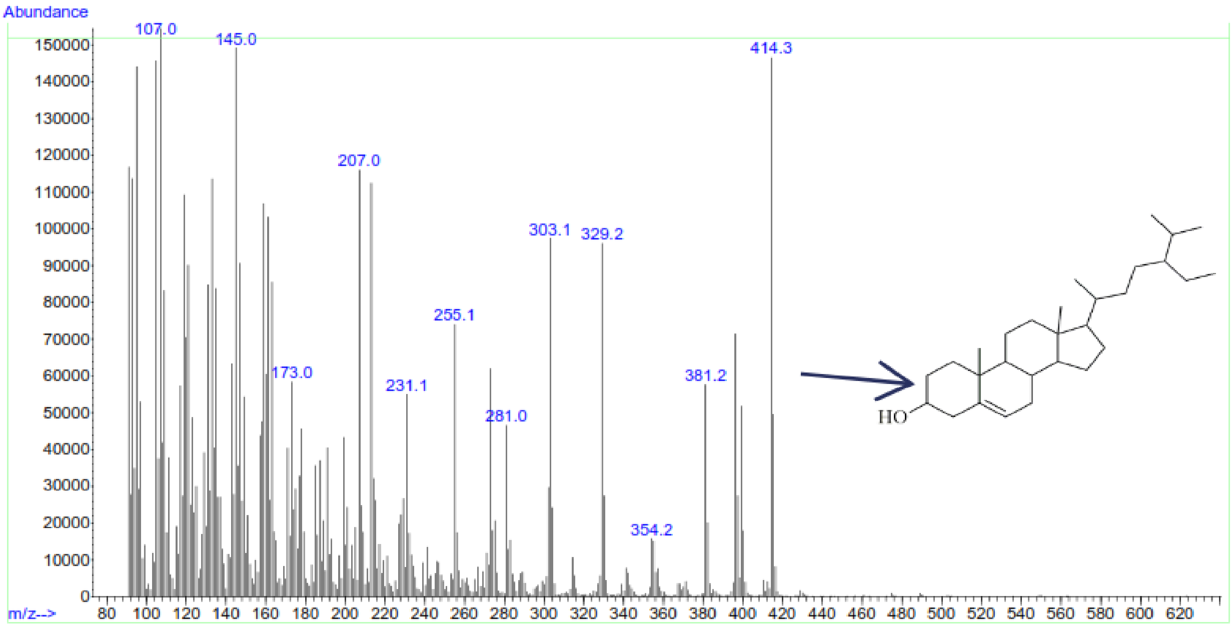

Figure S6: MS of ethyl acetate extract of root at 14.9 retention time.

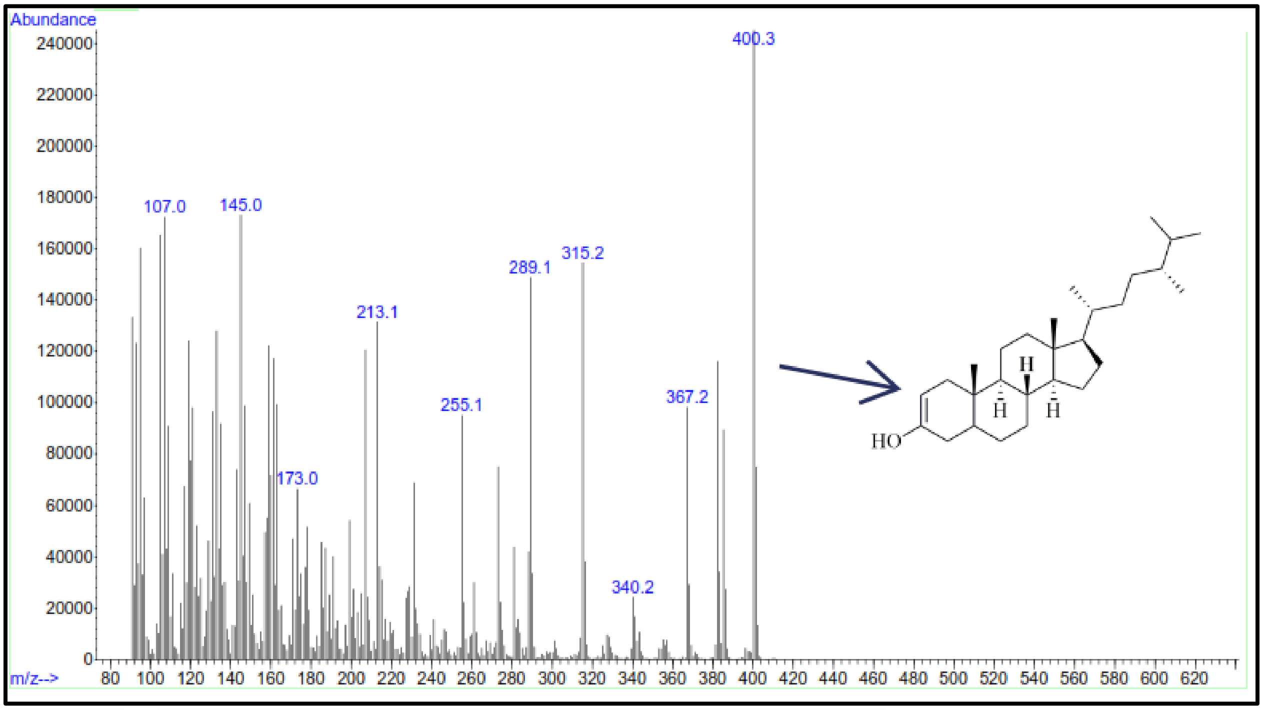

Figure S7: MS of ethyl acetate extract of root at 14.5 retention time.

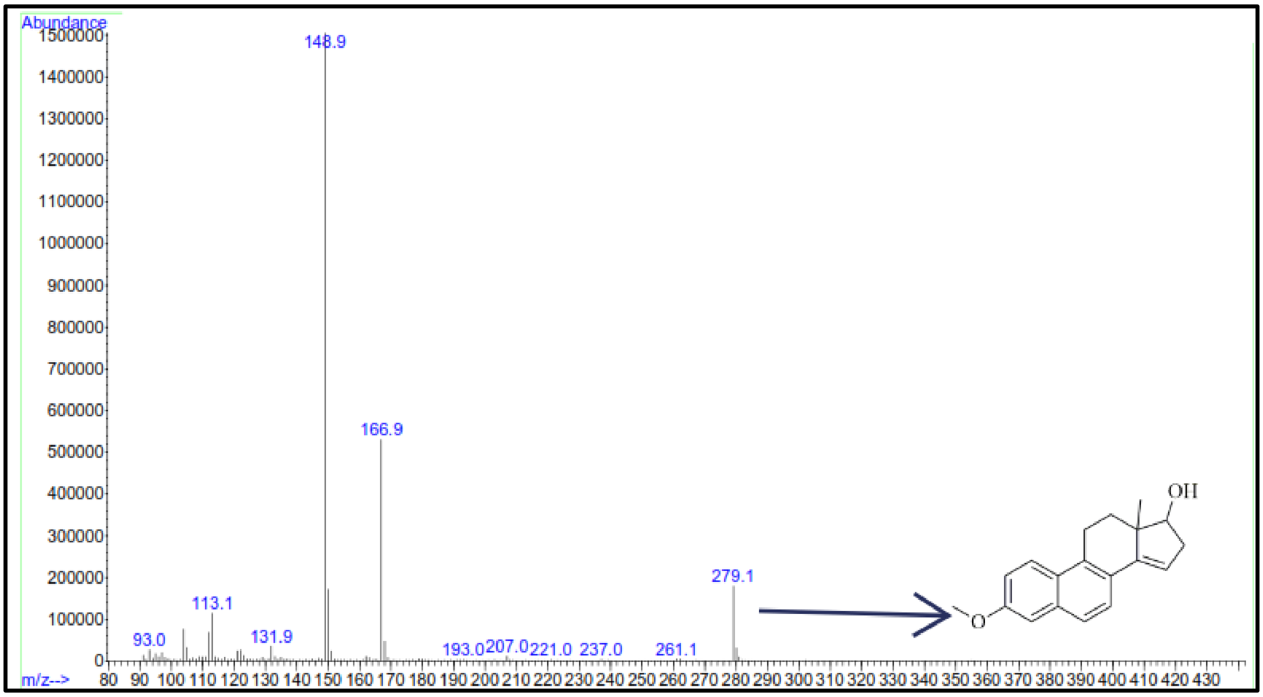

Figure S8: MS of ethyl acetate extract of root at 11.6 retention time.

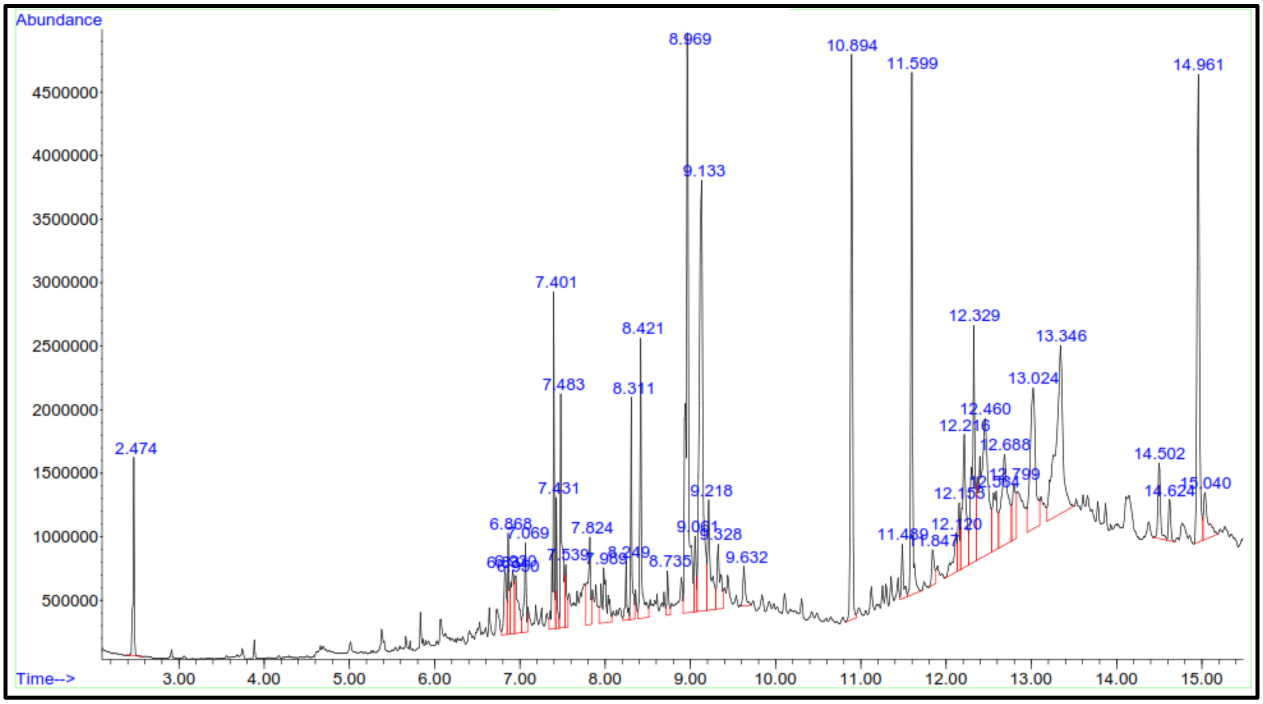

Figure S9: GC spectrum of aqueous extract of root

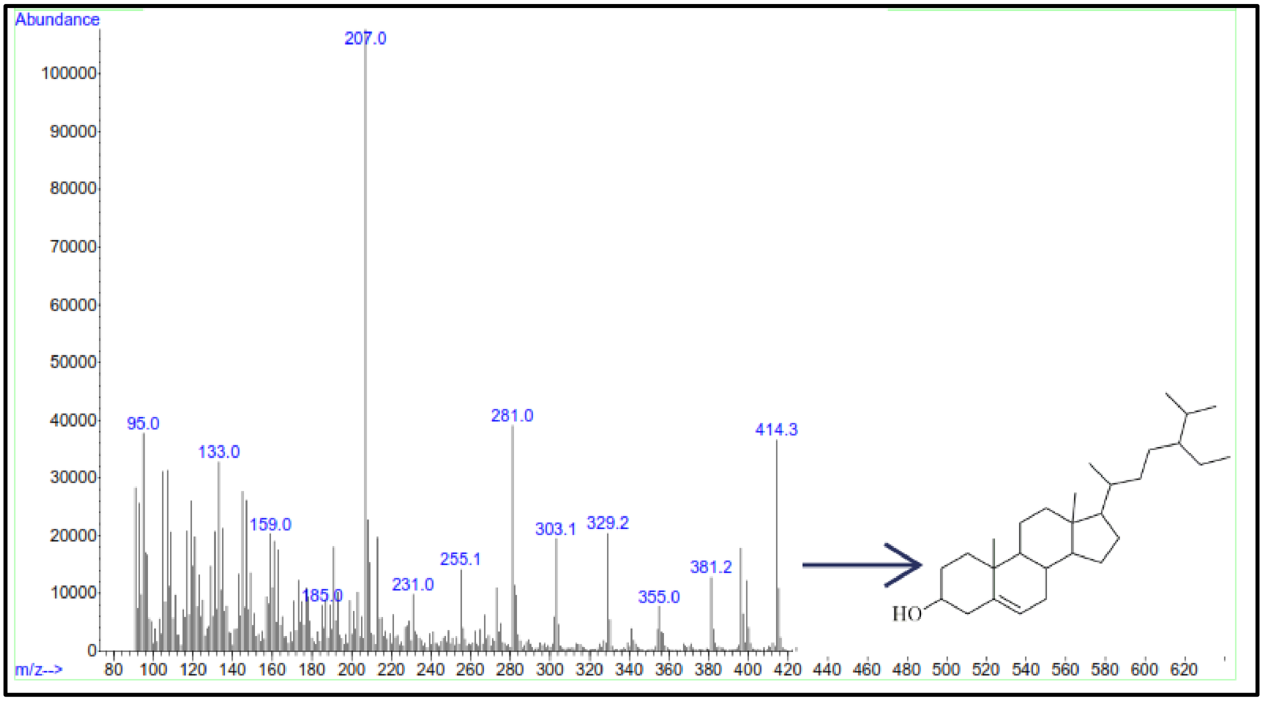

Figure S10: Mass spectrum of aqueous extract of root at 14.9 retention time.

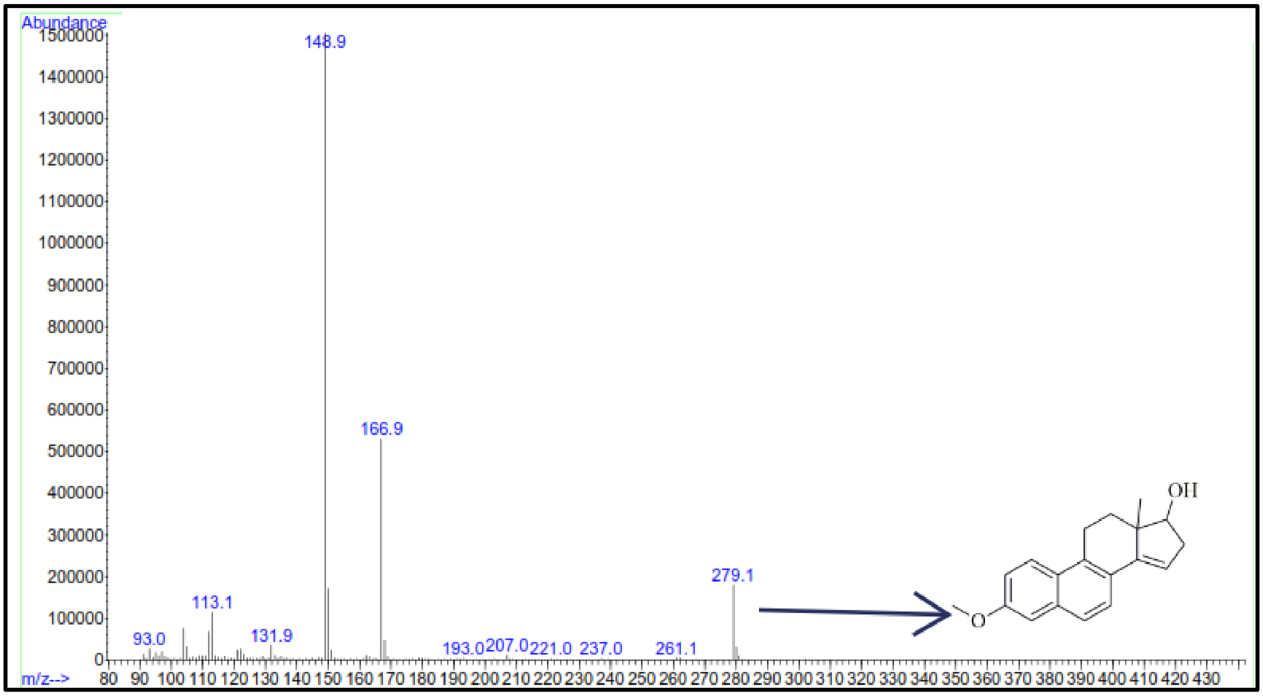

Figure S11: MS of aqueous extract of root at 11.6 retention time.

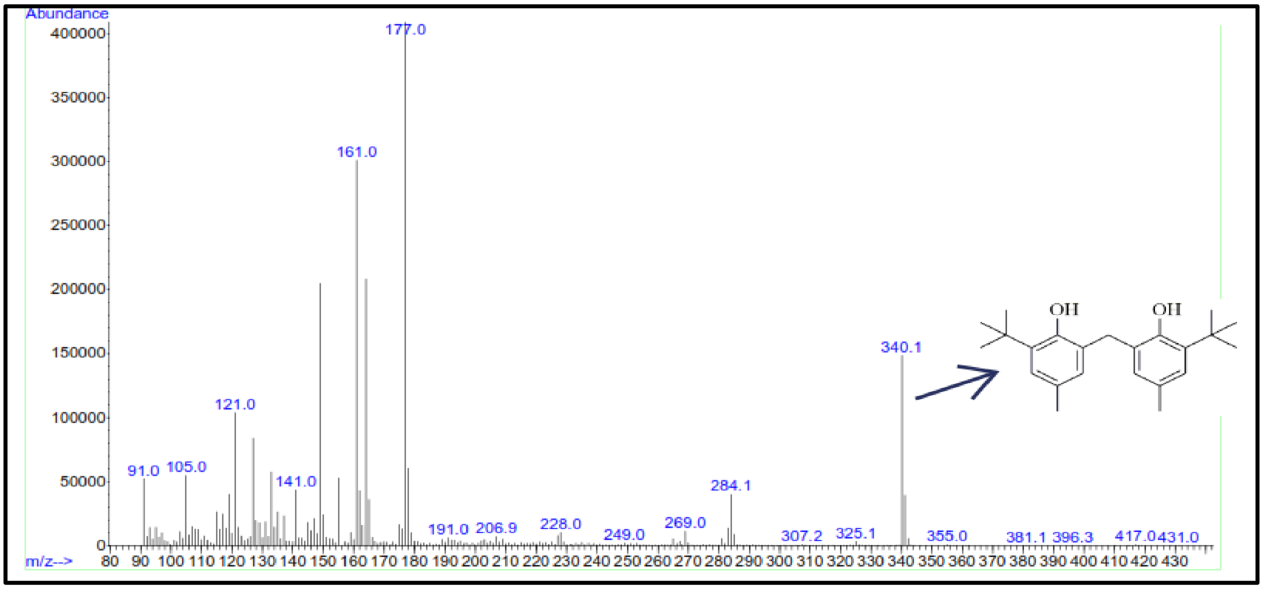

Figure S12: MS of aqueous extract of root at 10.8 retention time.

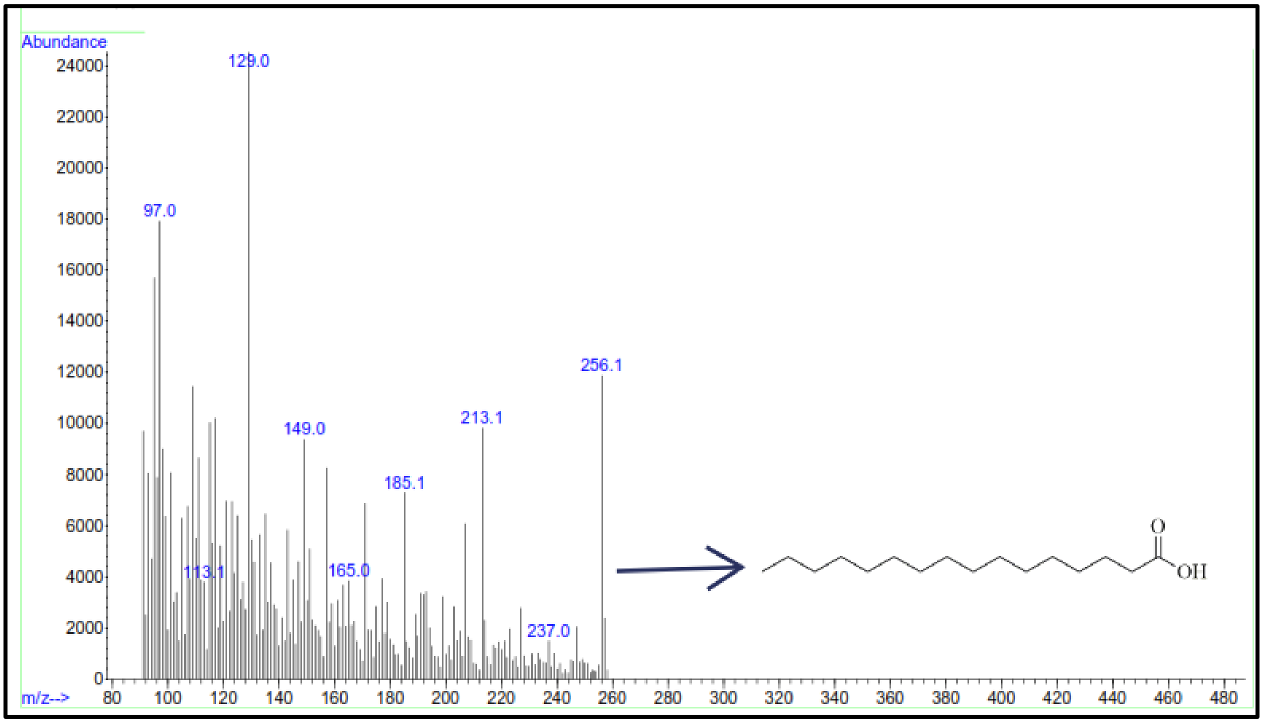

Figure S13: MS of aqueous extract of root at 8.4 retention time.

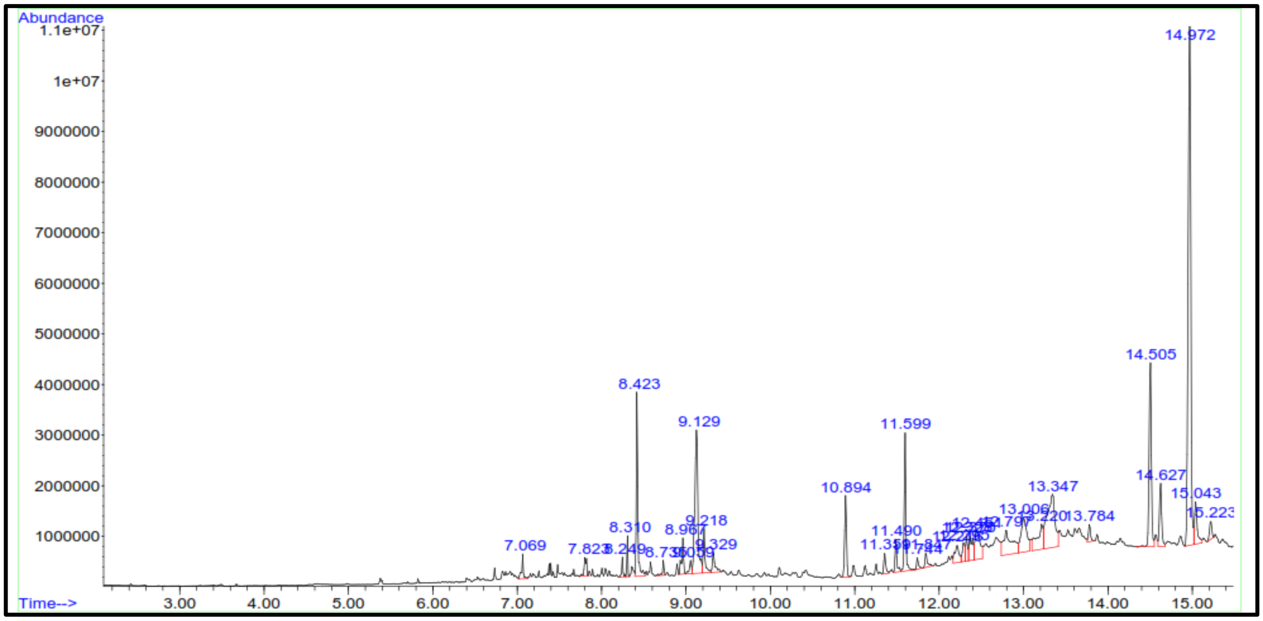

Figure S14: GC spectrum ethanolic extract of root

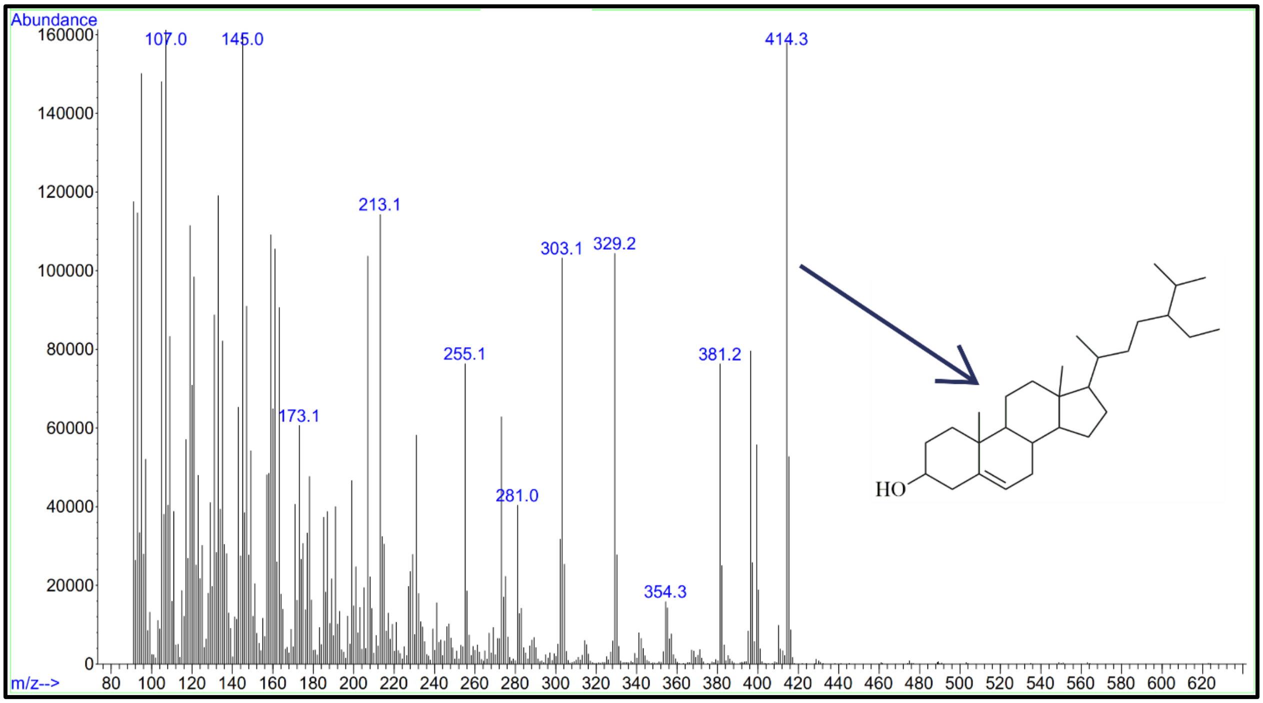

Figure S15: MS of ethanolic extract of root at 14.9 retention time.

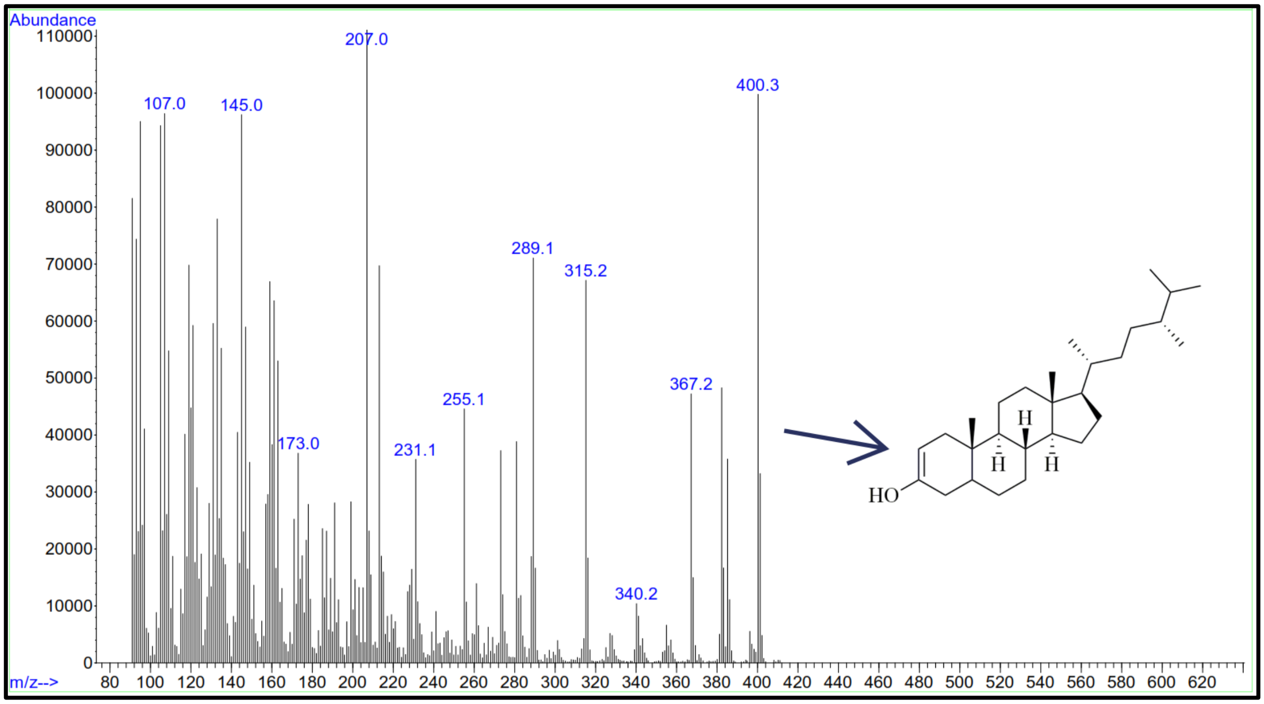

Figure S16: MS of ethanolic extract of root at 14.4 retention time.

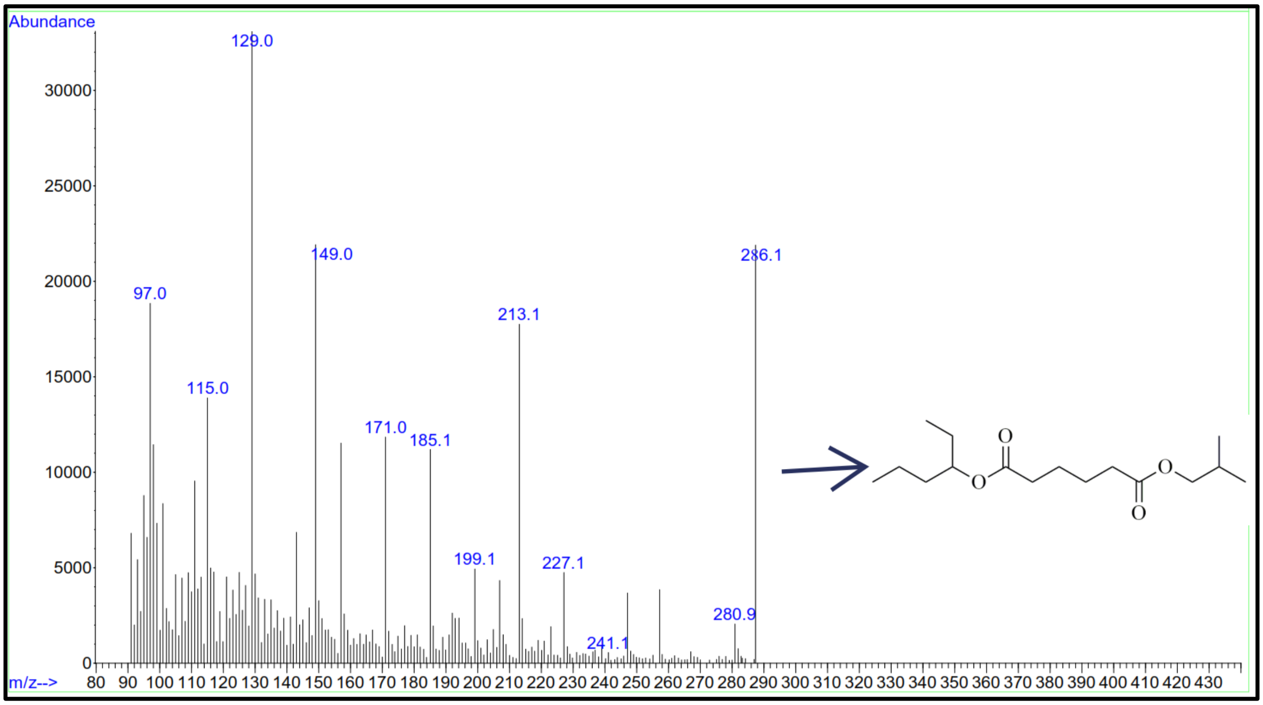

Figure S17: MS of ethanolic extract of root at 8.4 retention time.

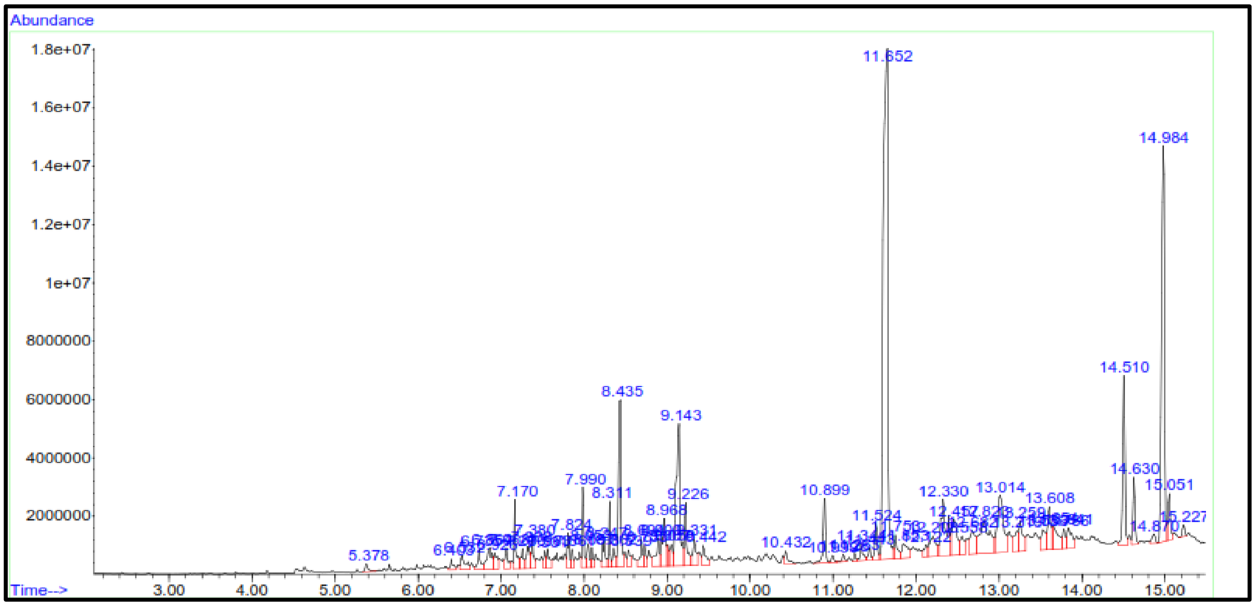

Figure S18: GC spectrum of ethyl acetate extract of stem.

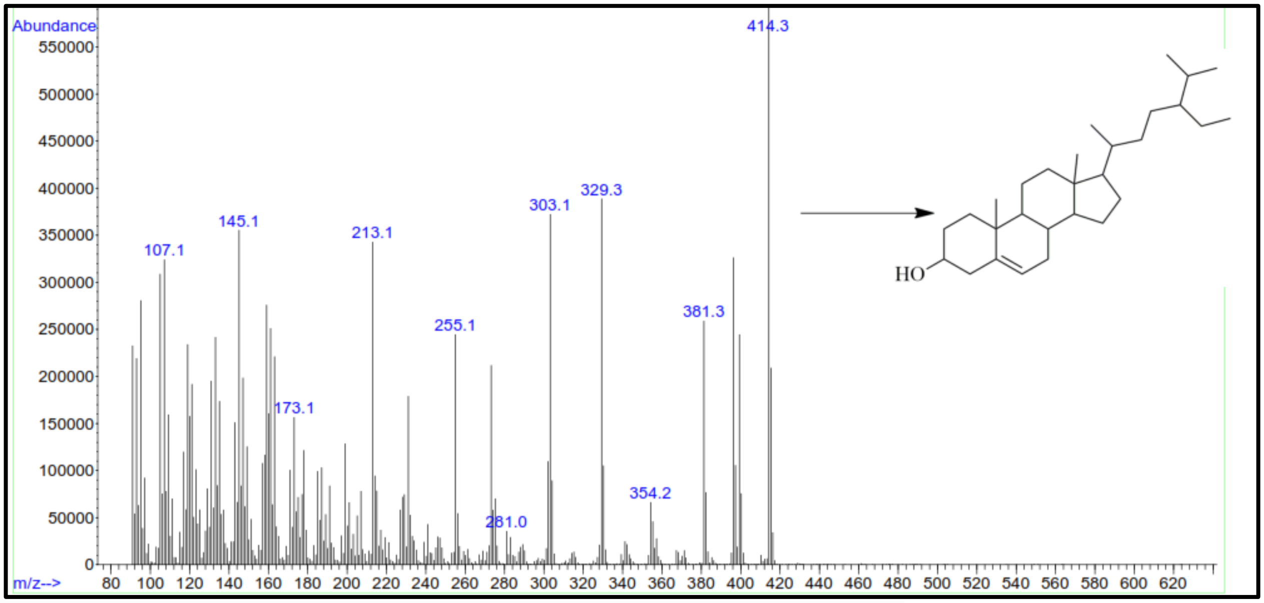

Figure S19: MS of ethyl acetate extract of stem at 14.9 retention time.

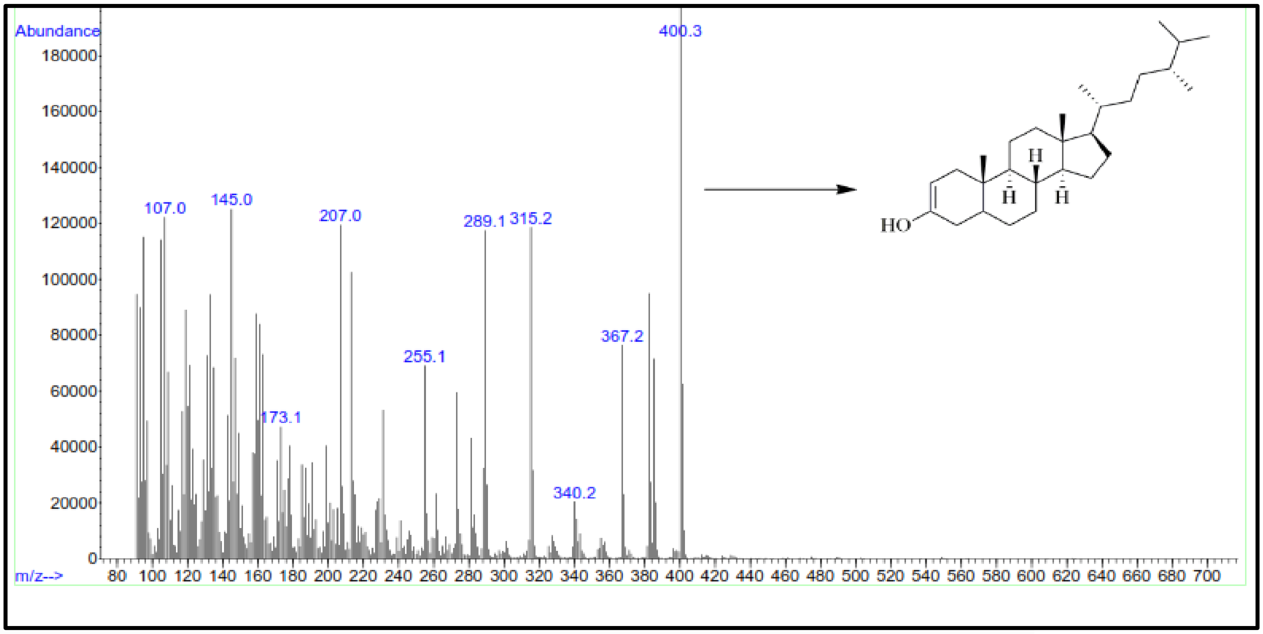

Figure S20: Mass spectrum of ethyl acetate extract of stem at 14.5 retention time.

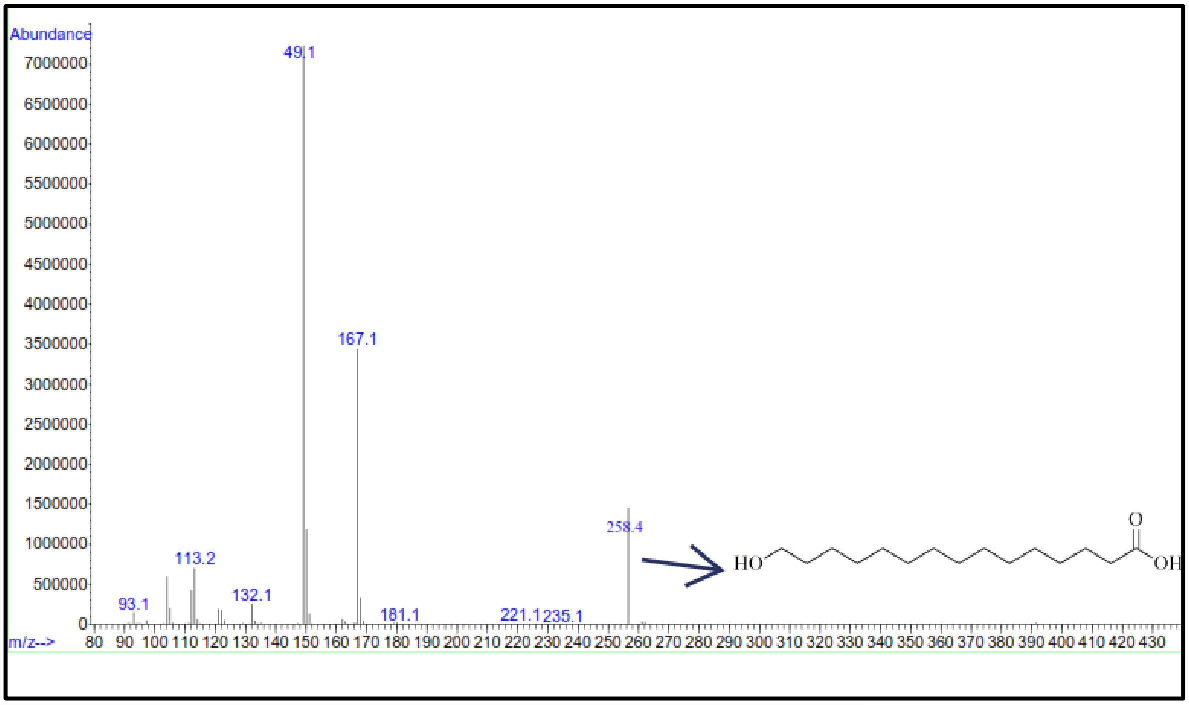

Figure S21: MS of ethyl acetate extract of stem at 11.6 retention time

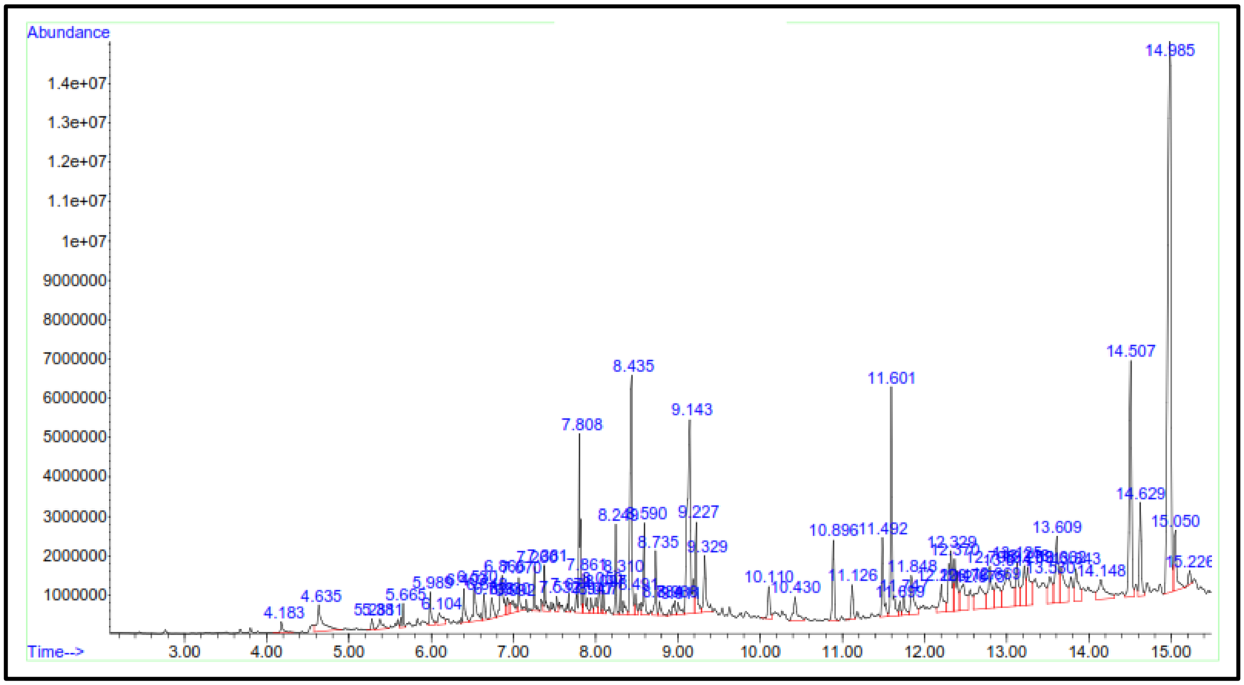

Figure S22: GC spectrum of ethanolic extract of stem

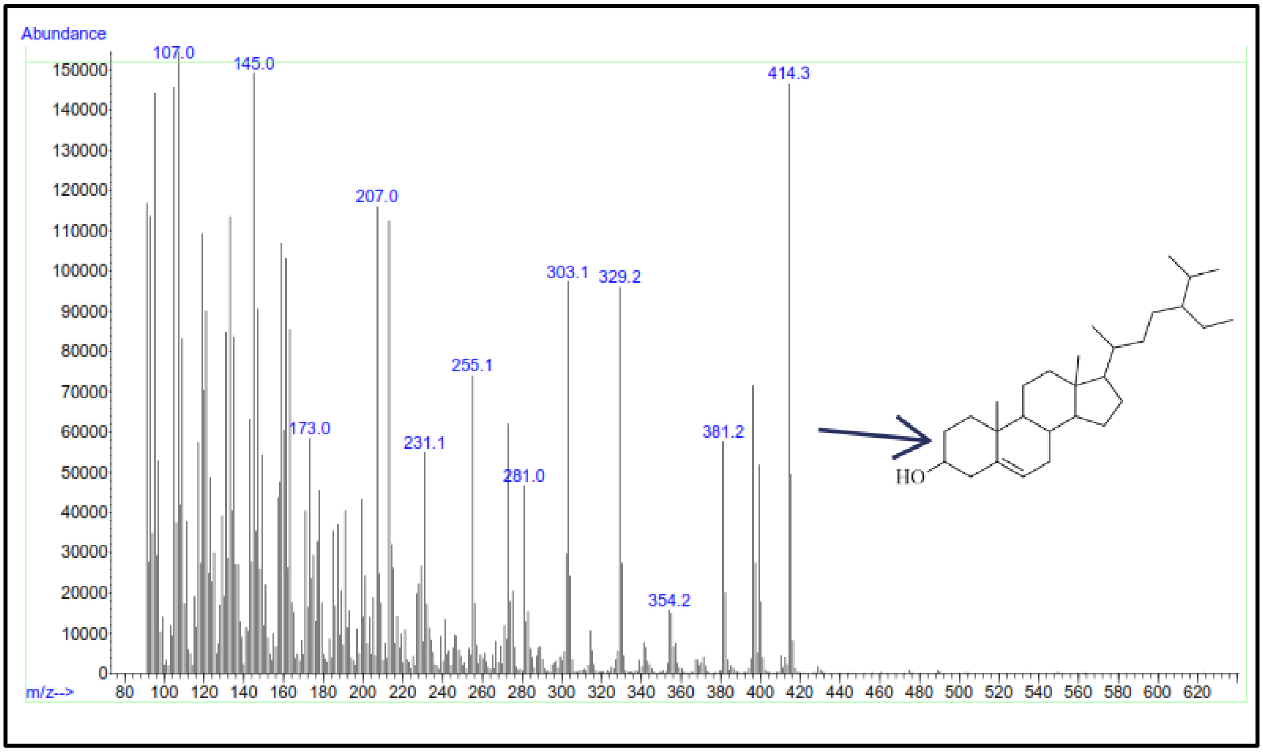

Figure S23: MS of ethanolic extract of stem at 14.9 retention time.

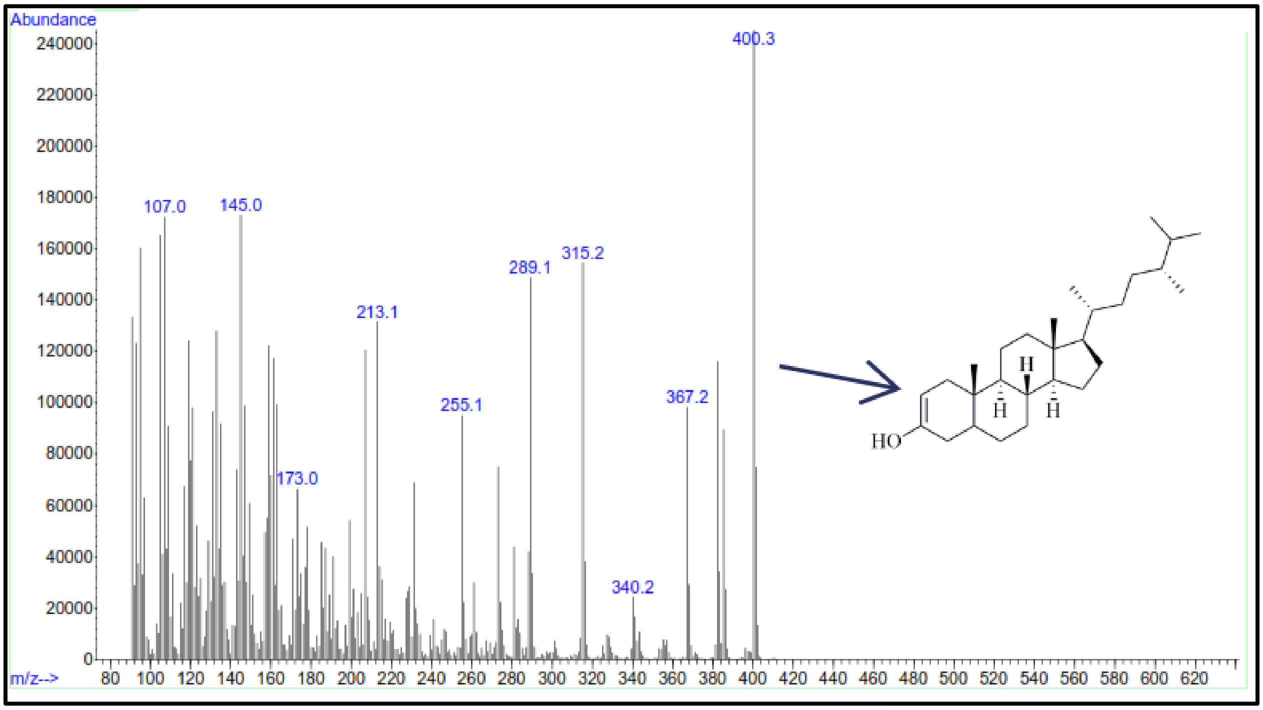

Figure S24: MS of ethanolic extract of stem at 14.5 retention time.

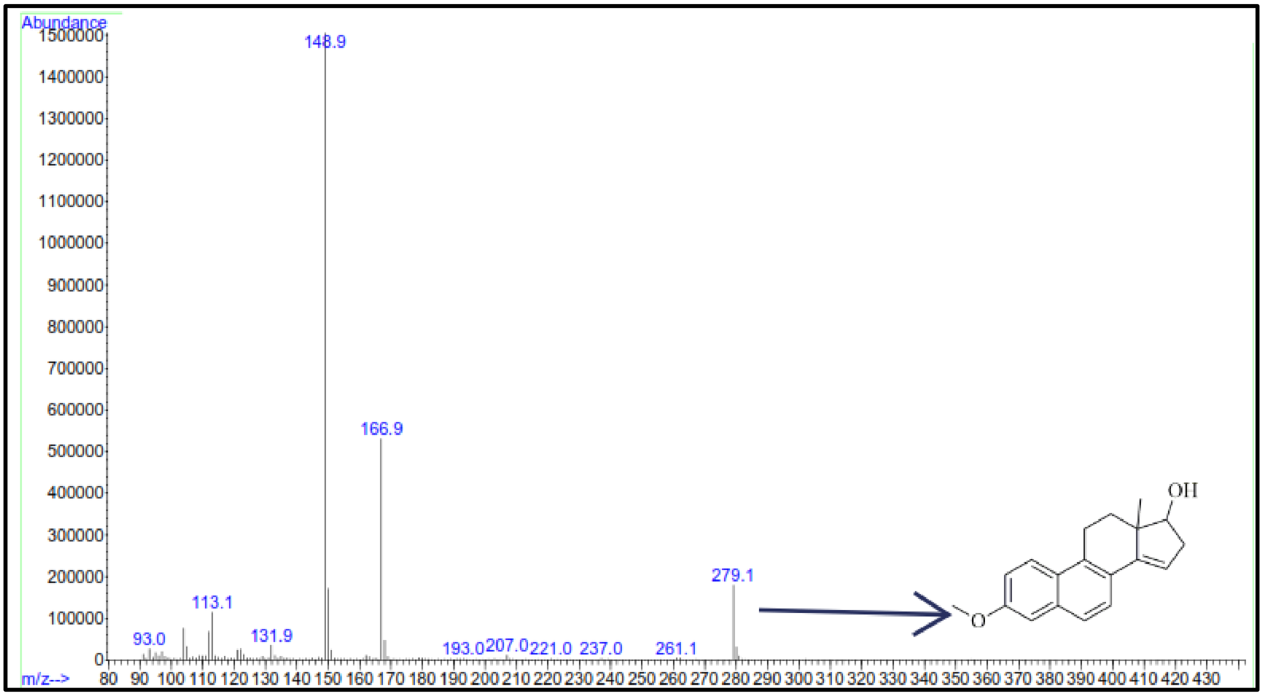

Figure S25: MS of ethanolic extract of stem at 11.6 retention time.

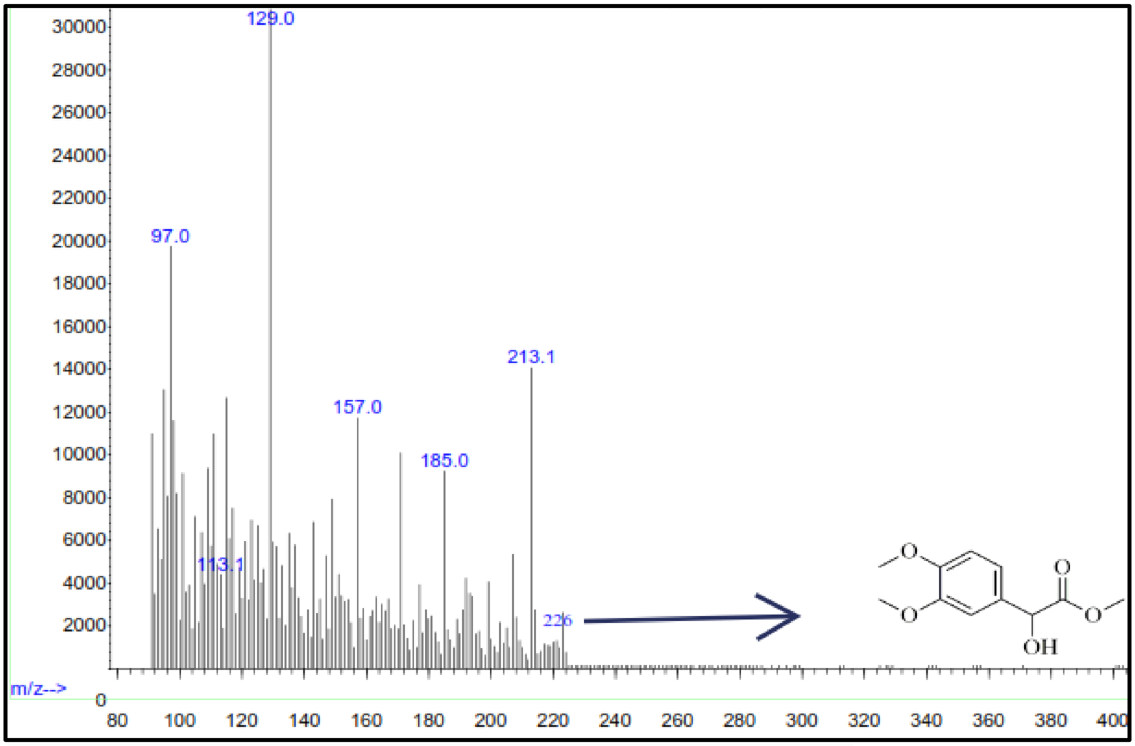

Figure S26: MS of ethanolic extract of stem at 8.4 retention time.

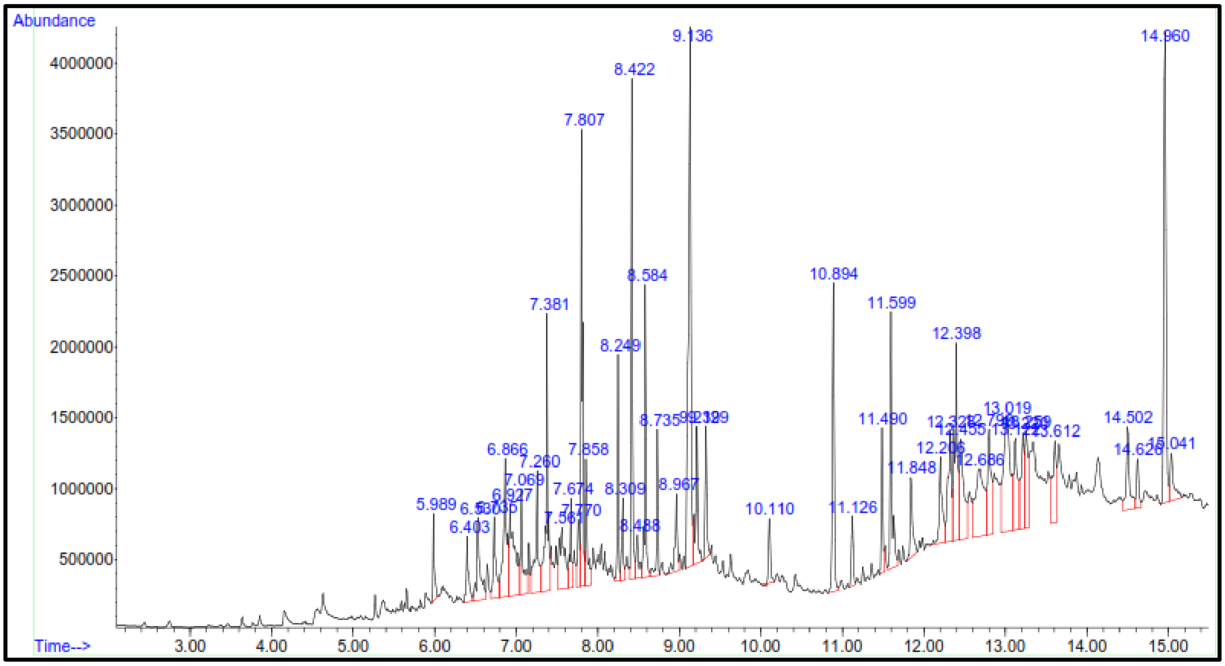

Figure S27: GC spectrum of aqueous extract of stem

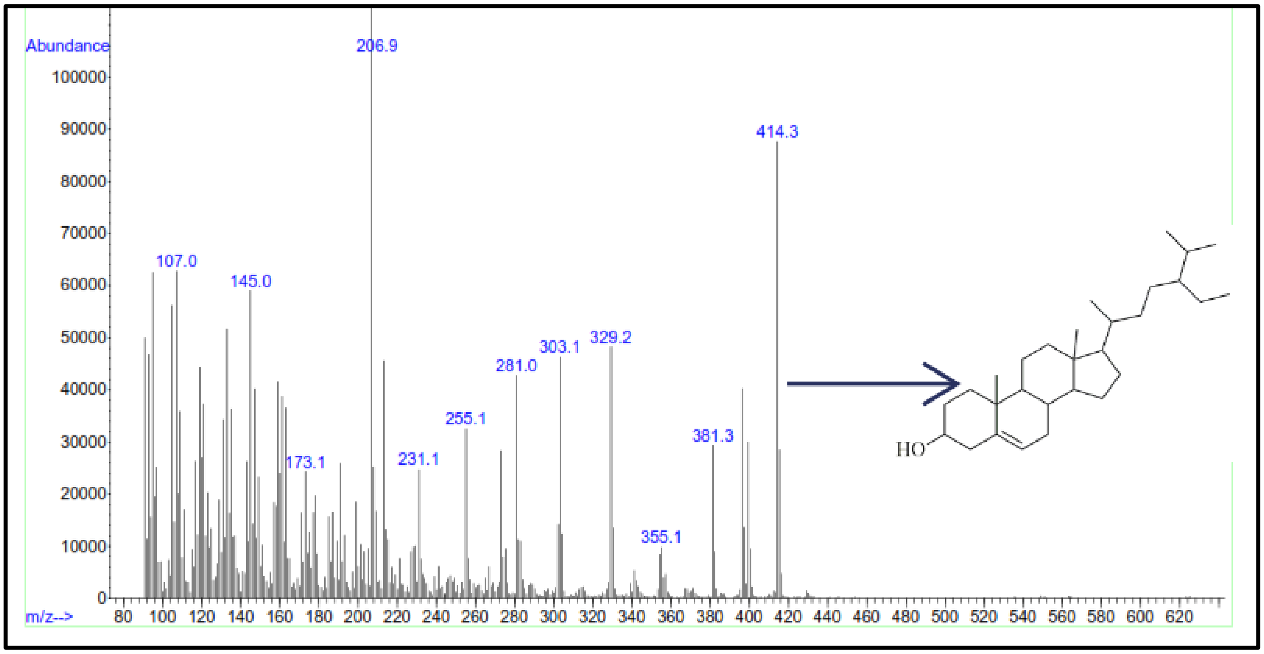

Figure S28: MS of aqueous extract of stem at 14.9 retention time.

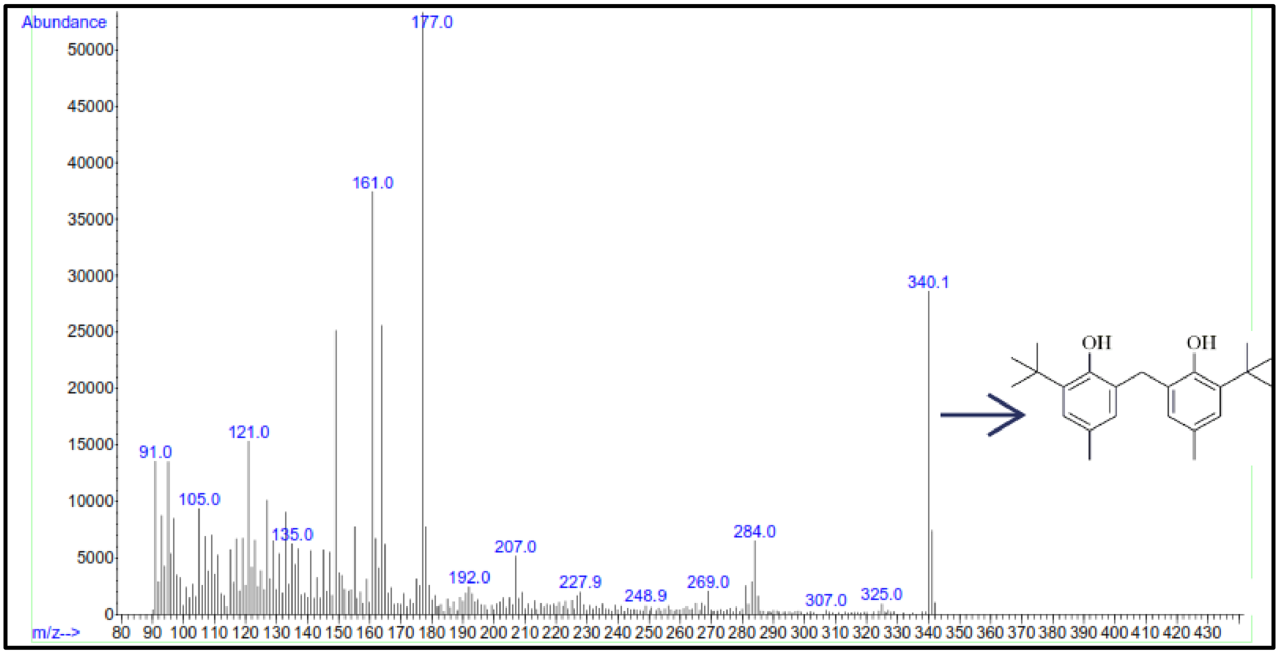

Figure S29: MS of aqueous extract of stem at 10.8 retention time.

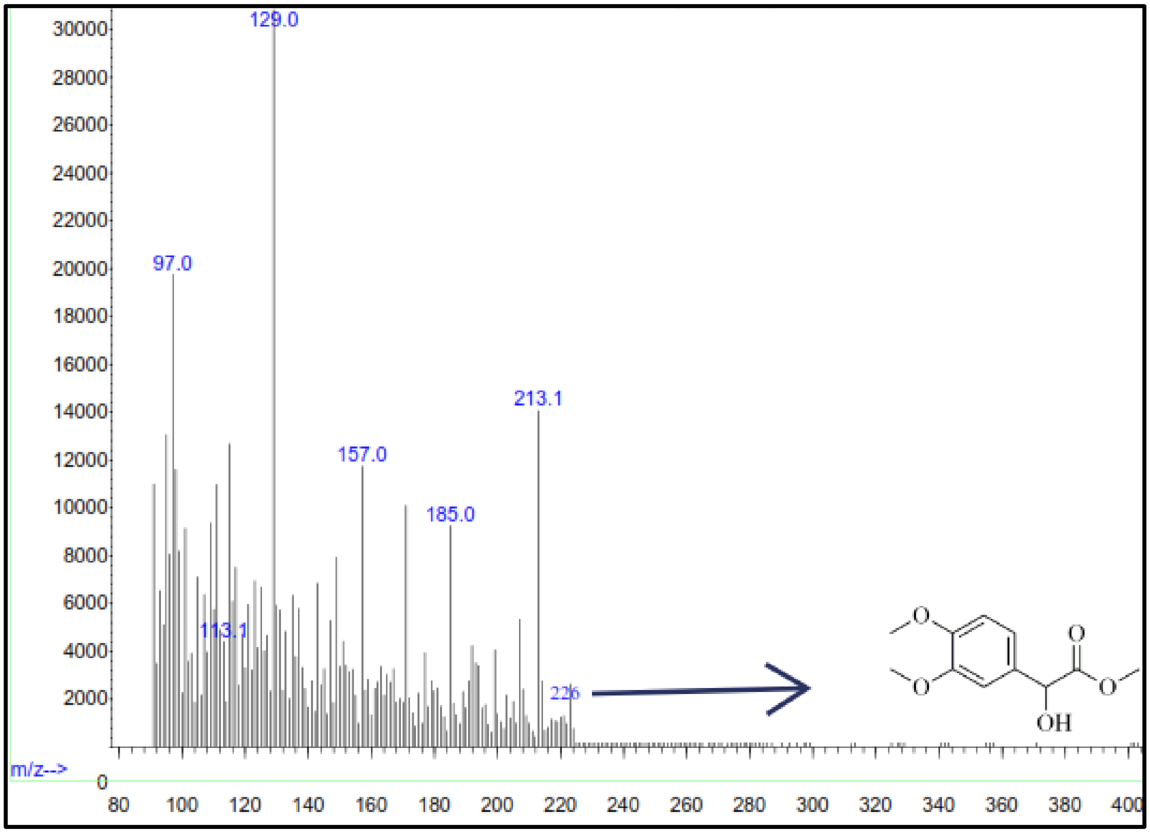

Figure S30: MS of aqueous extract of stem at 8.4 retention time.

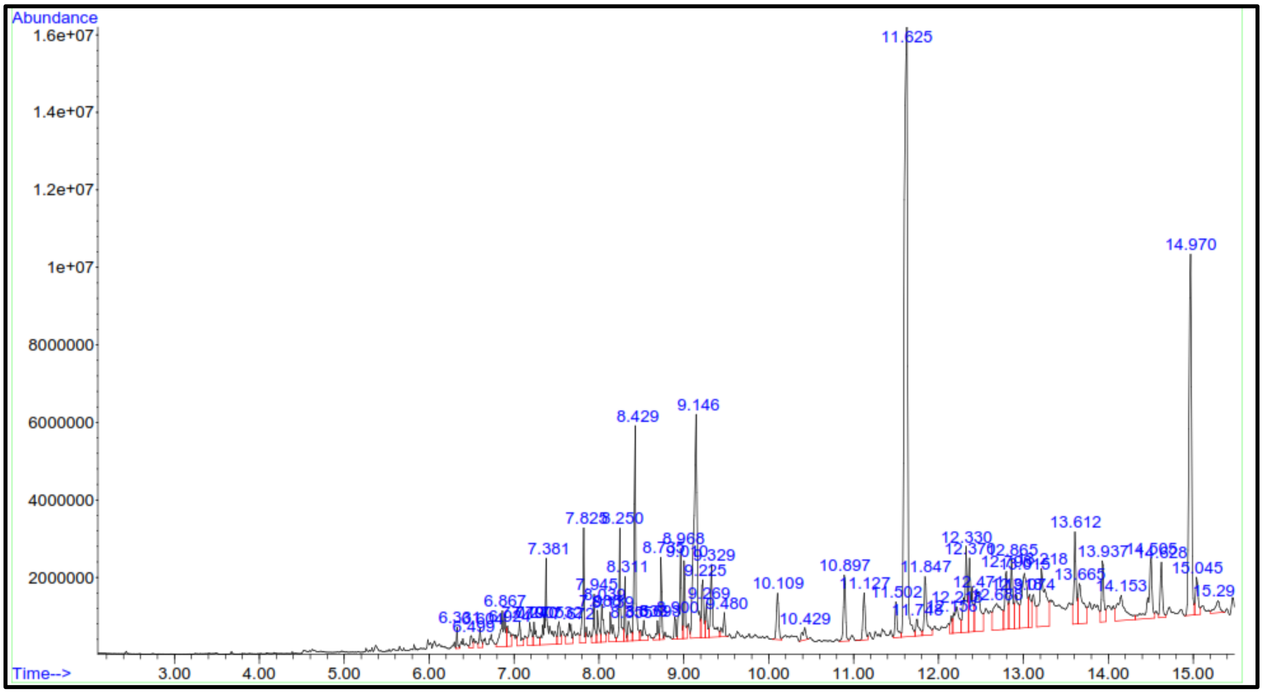

Figure S31: GC spectrum of ethyl acetate extract of leaf

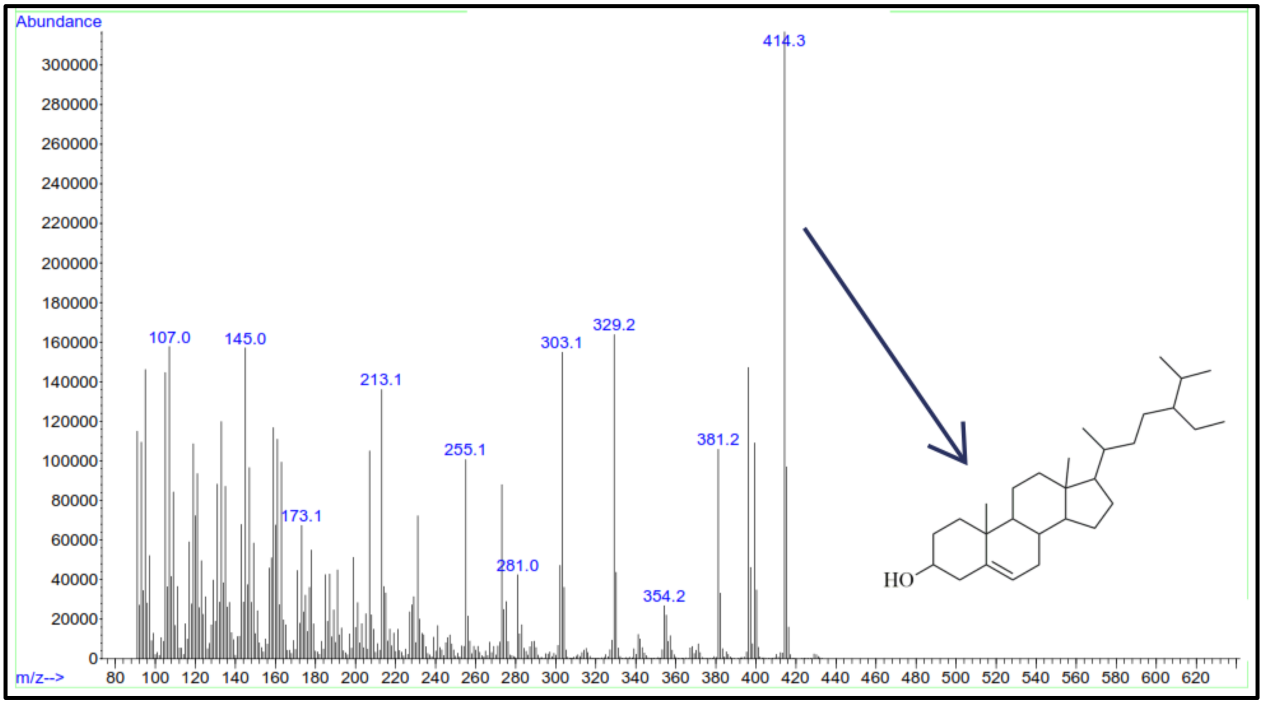

Figure S32: MS of ethyl acetate extract of leaf at 14.9 retention time.

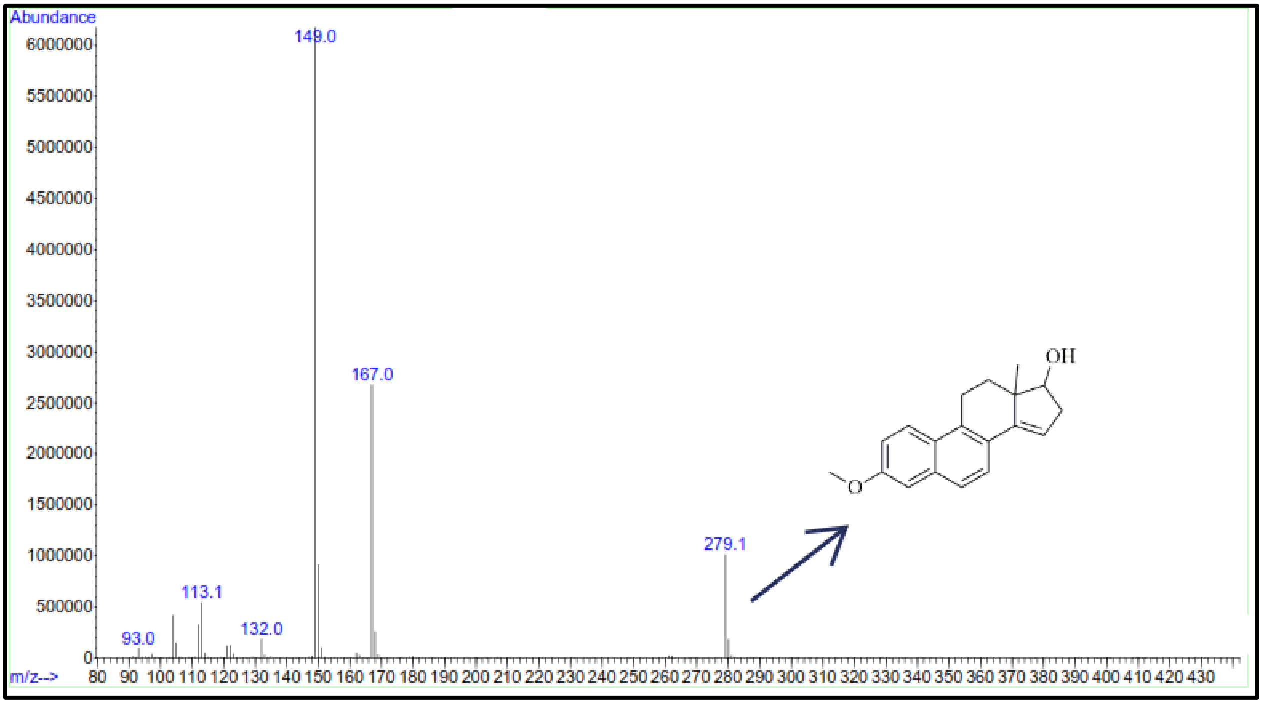

Figure S33: MS of ethyl acetate extract of leaf at 11.6 retention time

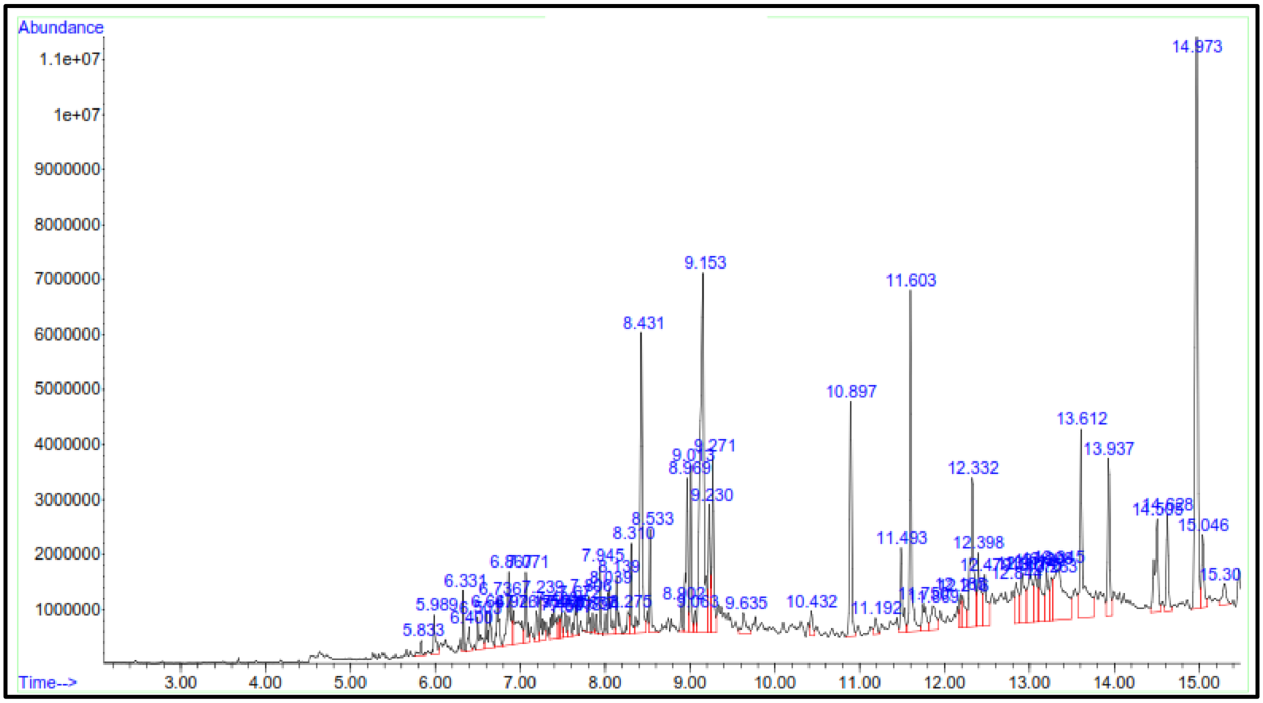

Figure S34: GC spectrum of ethanolic extract of leaf

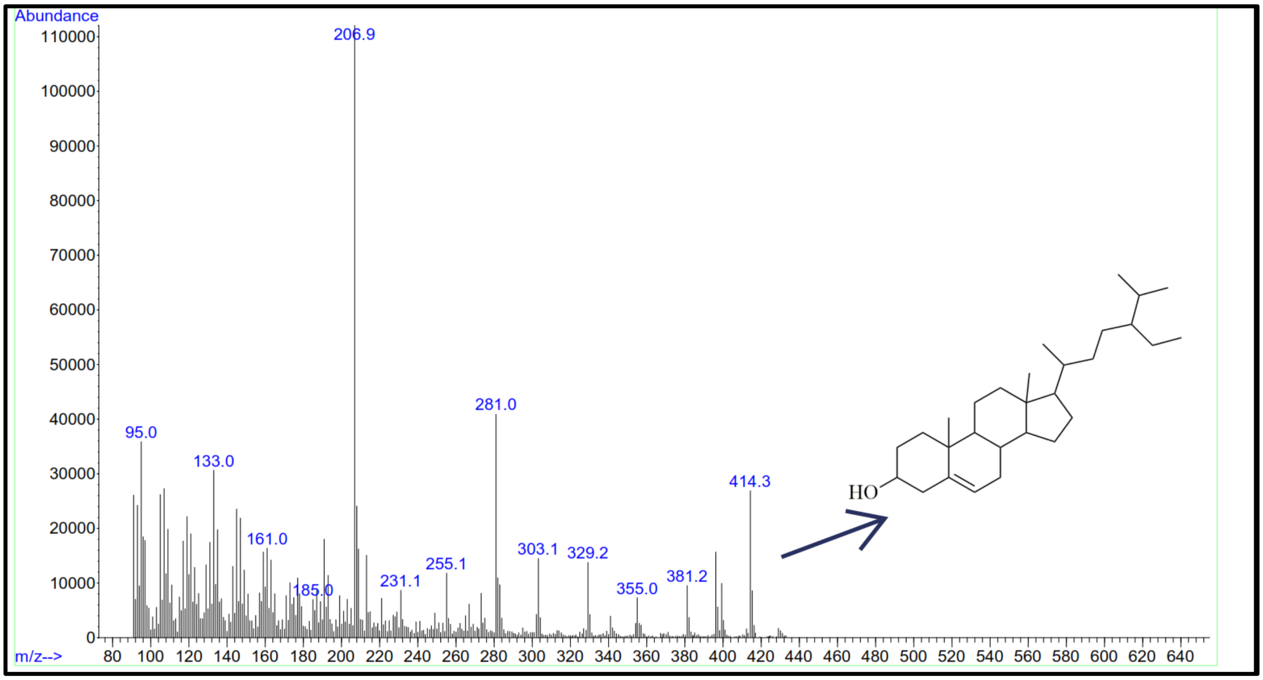

Figure S35: MS of ethanolic extract of leaf at 14.9 retention time.

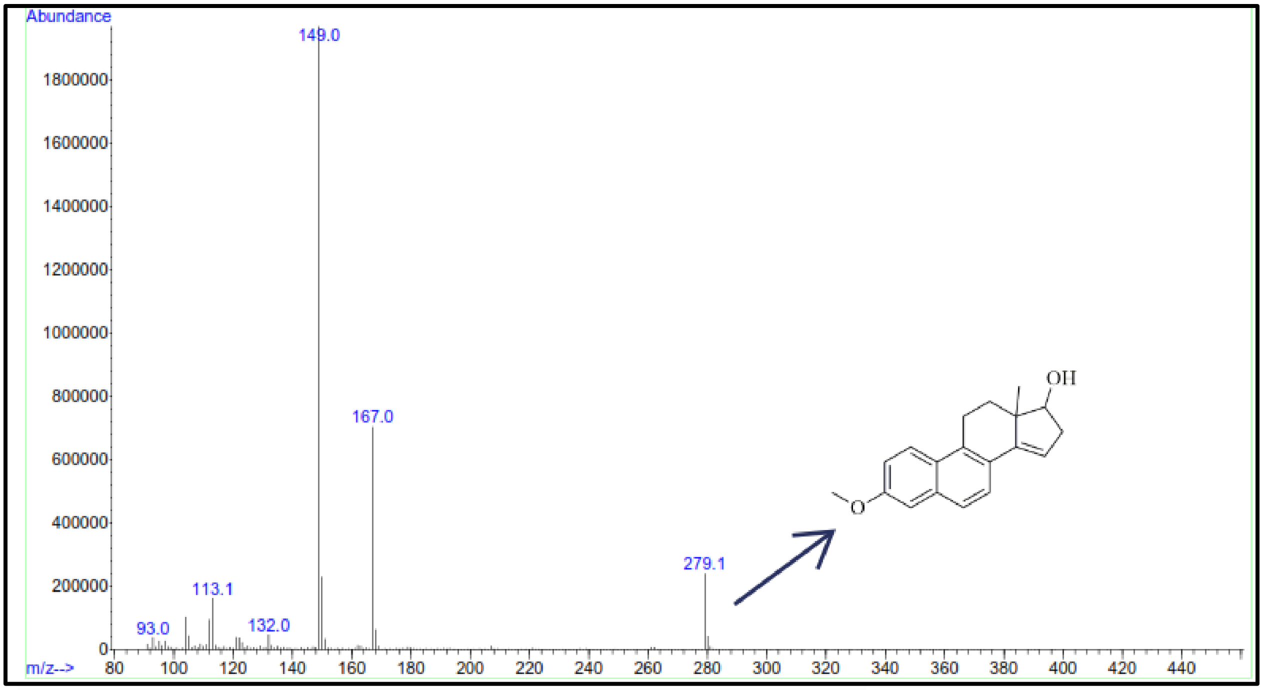

Figure S36: MS of ethanolic extract of leaf at 11.6 retention time.

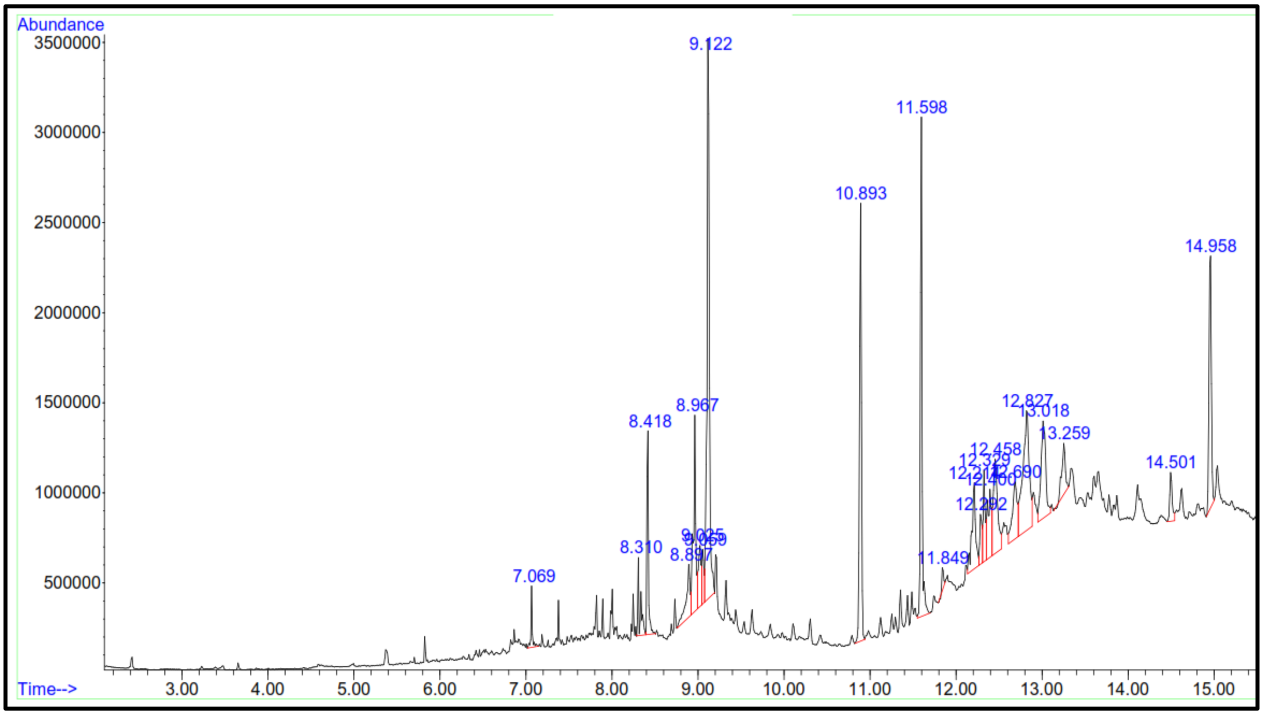

Figure S37: GC spectrum of aqueous extract of leaf.

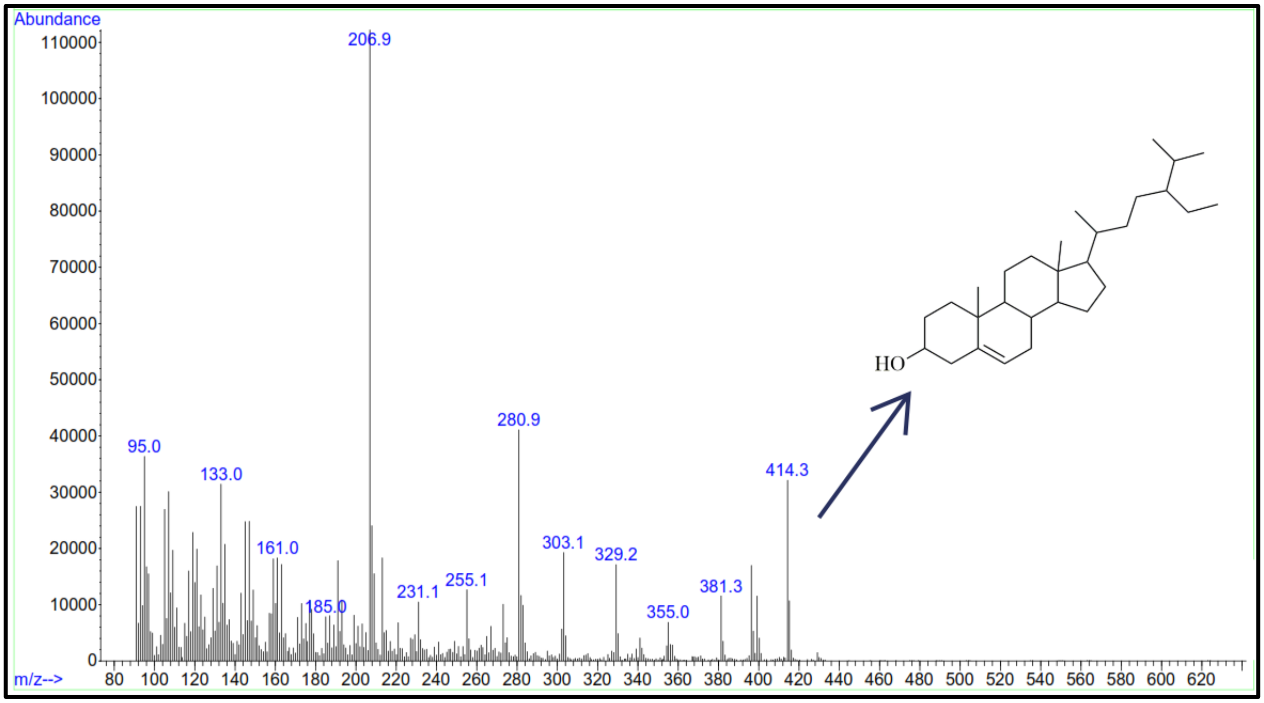

Figure S38: MS of aqueous extract of leaf at 14.9 retention time.

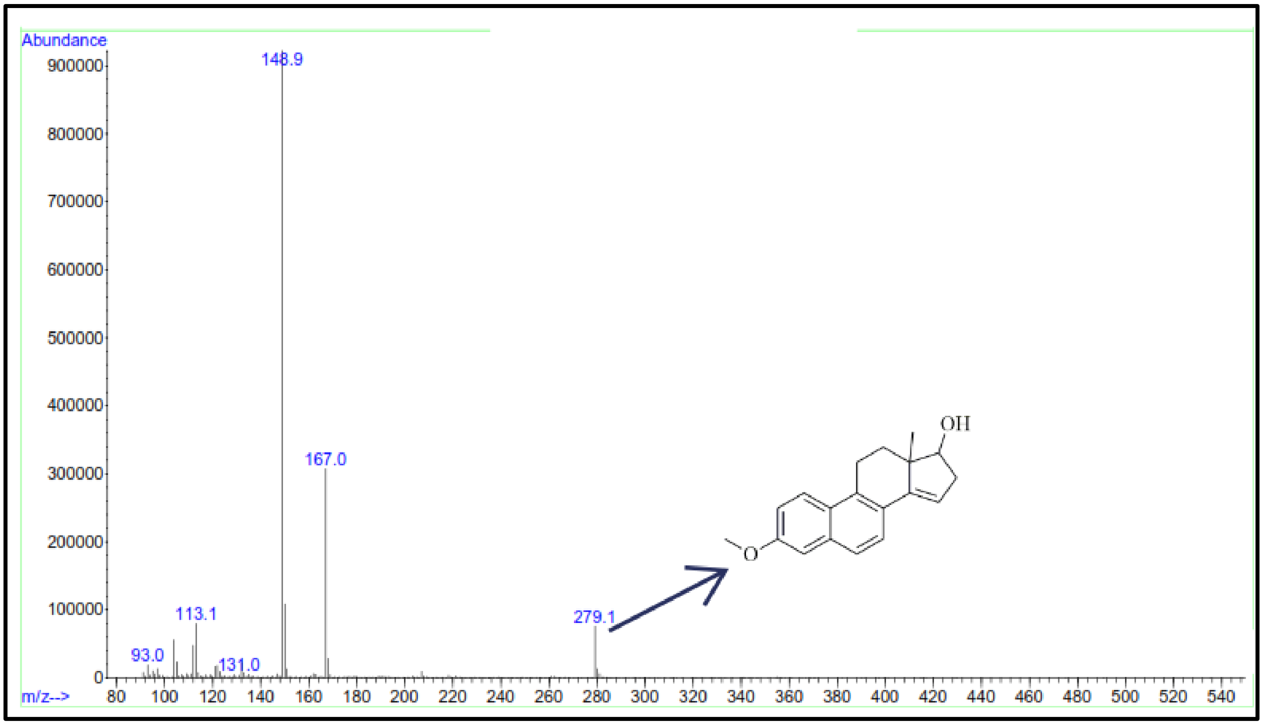

Figure S39: MS of aqueous extract of leaf at 11.5 retention time.

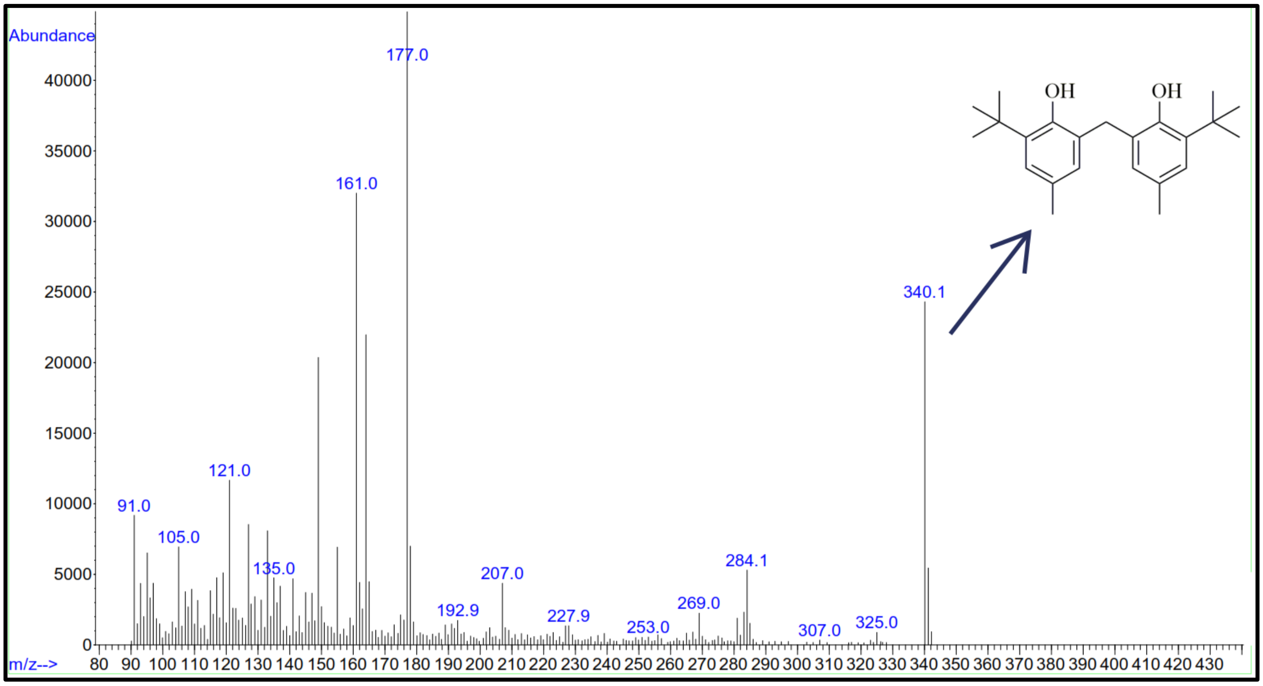

Figure S40: MS of aqueous extract of leaf at 10.9 retention time.

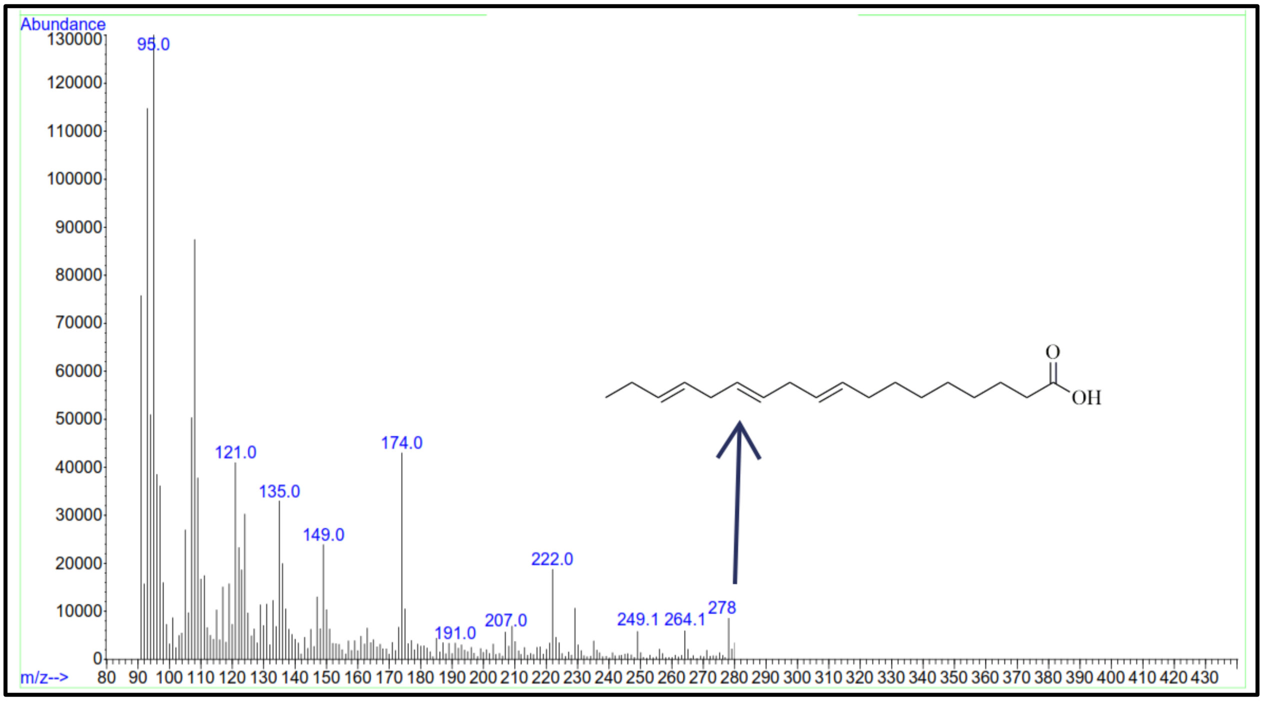

Figure S41: MS of aqueous extract of leaf at 9.1 retention time.

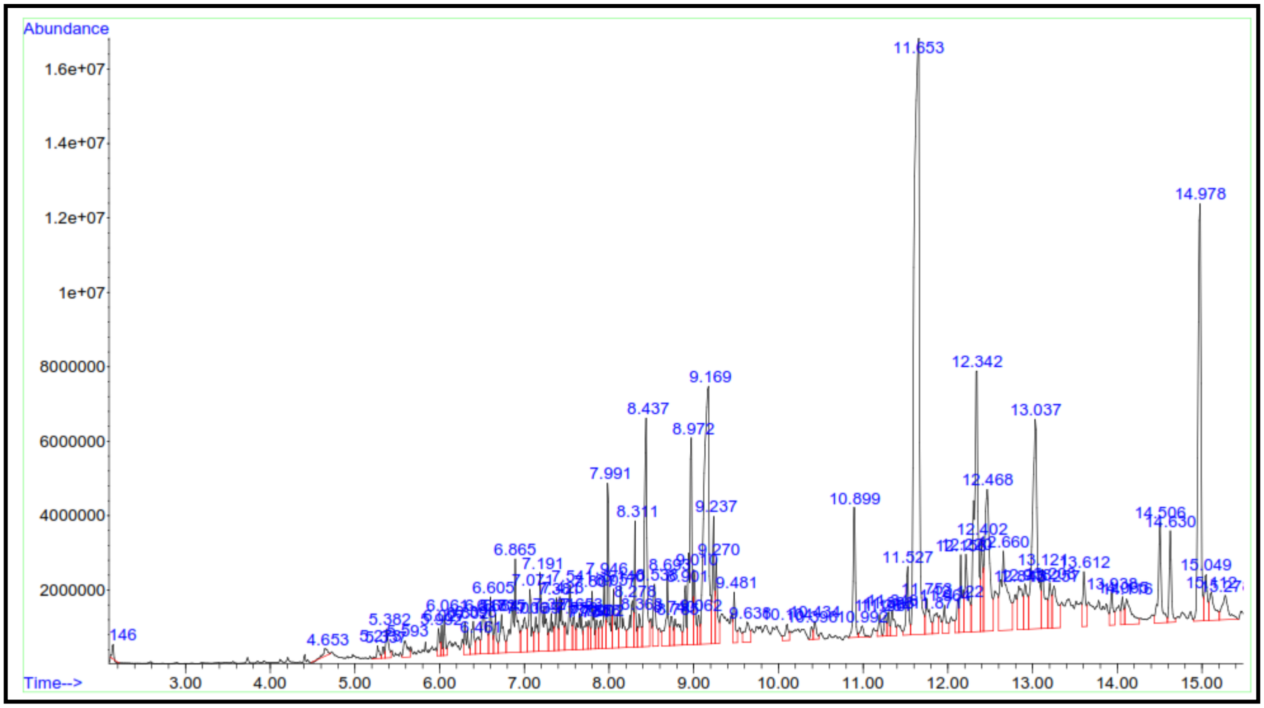

Figure S42: GC spectrum of ethyl acetate extract of flower

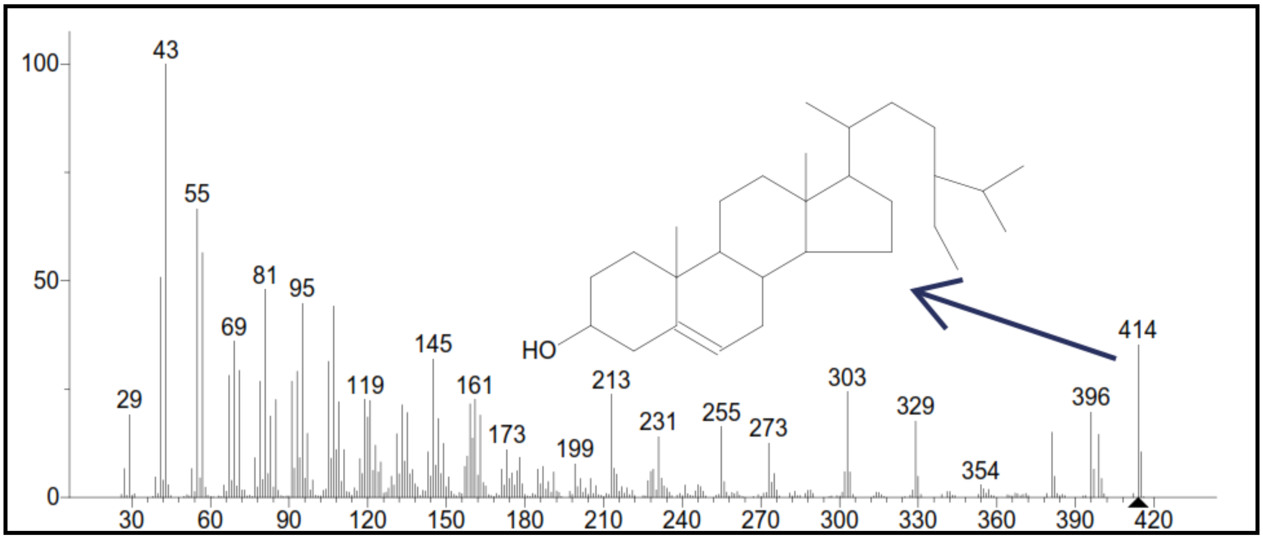

Figure S43: MS of ethyl acetate extract of flower at 14.9 retention time.

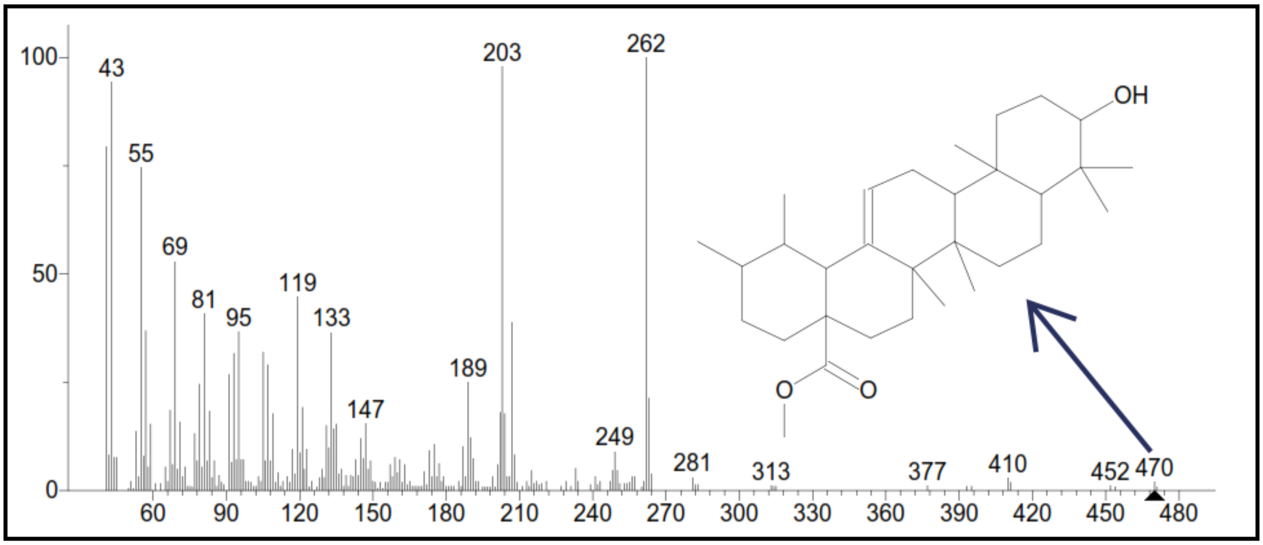

Figure S44: MS of ethyl acetate extract of flower at 13.0 retention time.

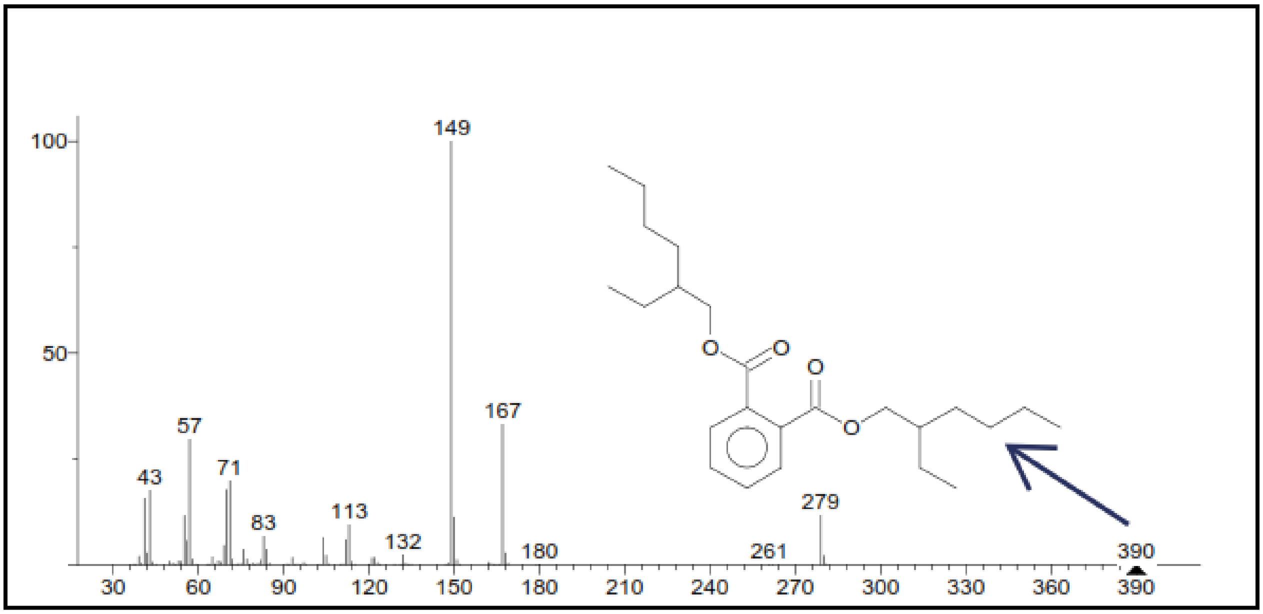

Figure S45: MS of ethyl acetate extract of flower at 11.62 retention time.

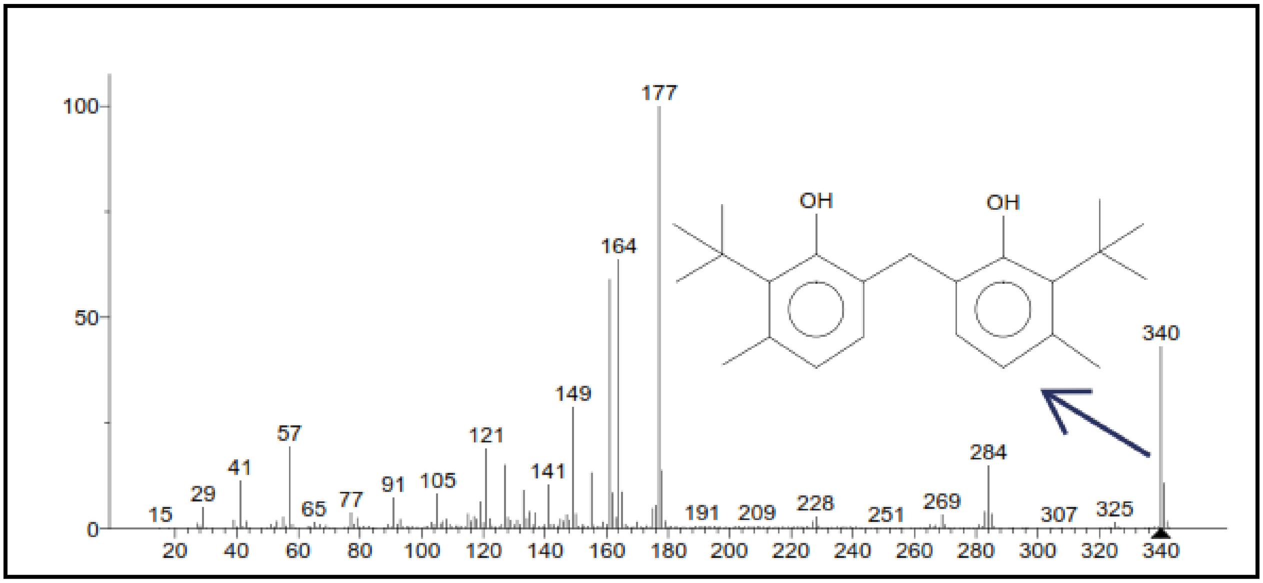

Figure S46: MS of ethyl acetate extract of flower at 10.8 retention time.

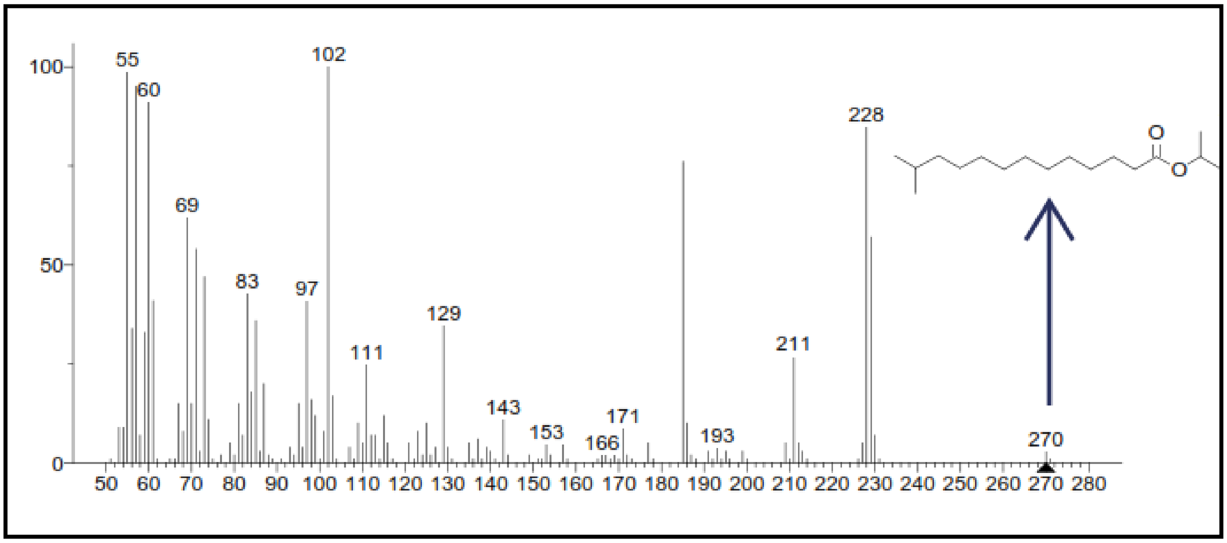

Figure S47: MS of ethyl acetate extract of flower at 7.9 retention time.

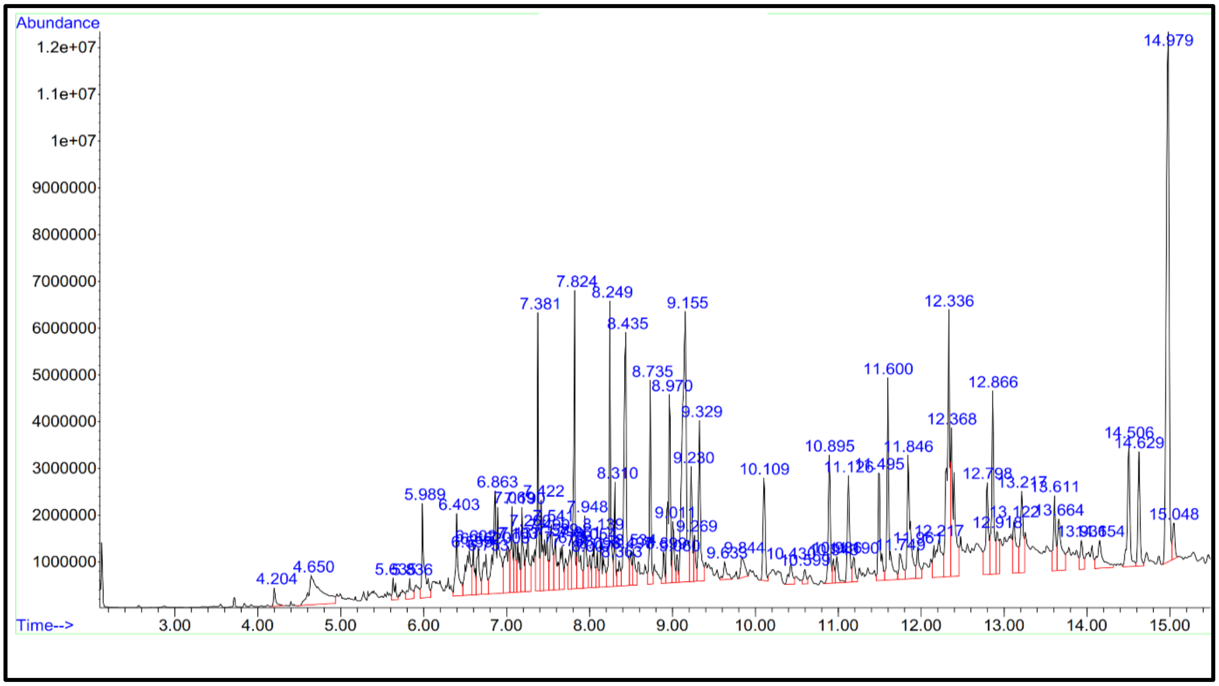

Figure S48: GC spectrum of ethanolic extract of flower

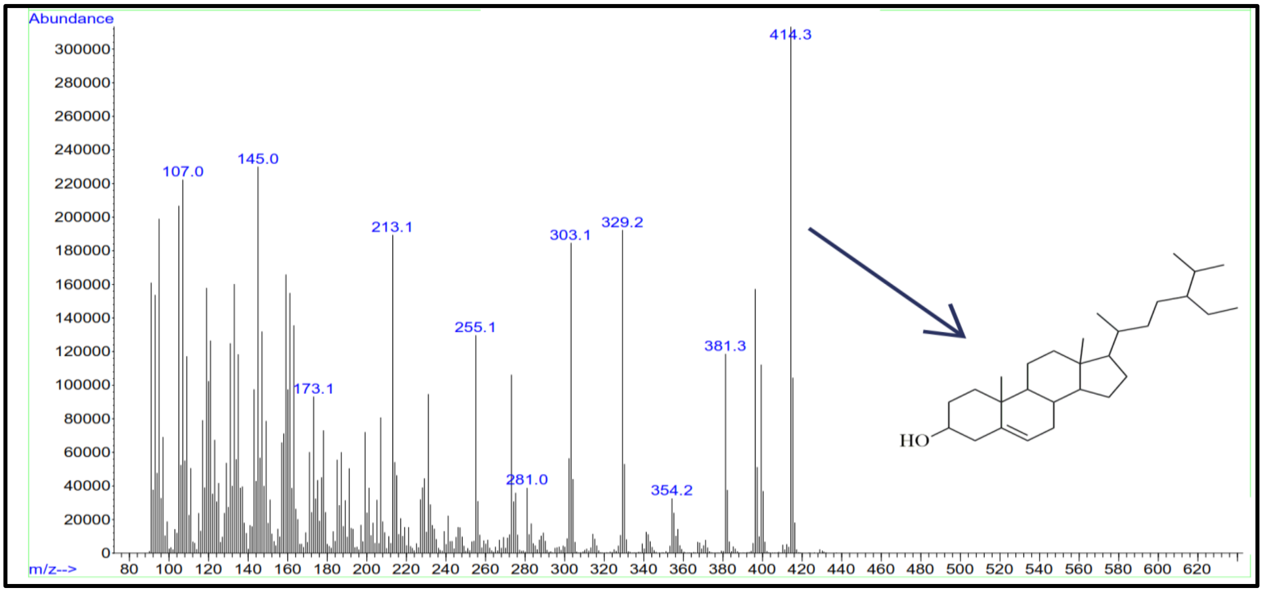

Figure S49: MS of ethanolic extract of flower at 14.9 retention time

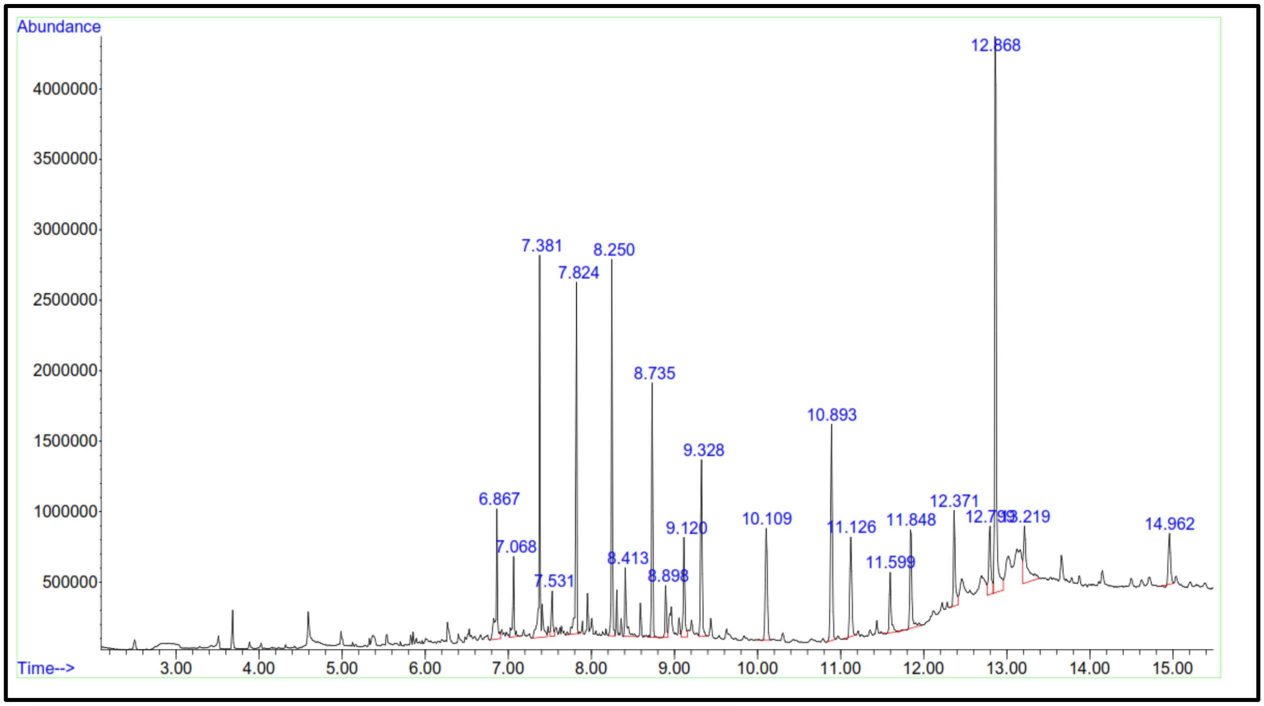

Figure S50: GC spectrum of aqueous extract of flower

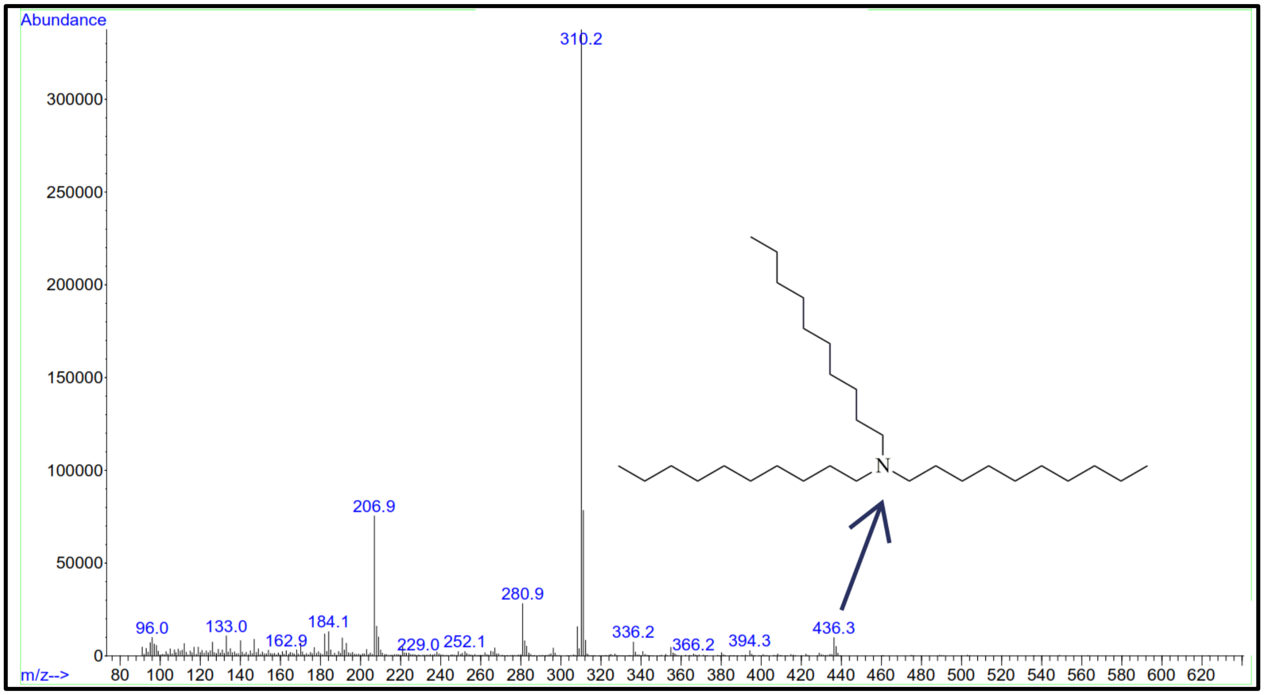

Figure S51: MS of aqueous extract of flower at 12.8 retention time.

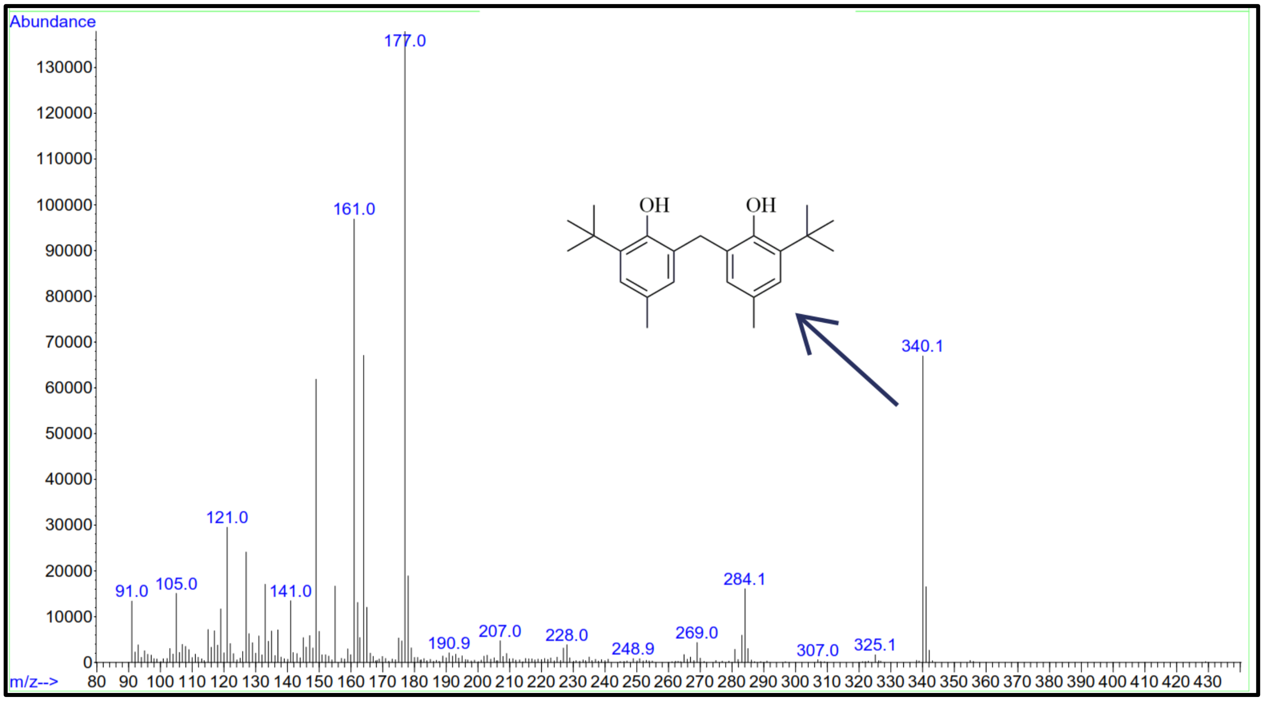

Figure S52: MS of aqueous extract of flower at 10.9 retention time.

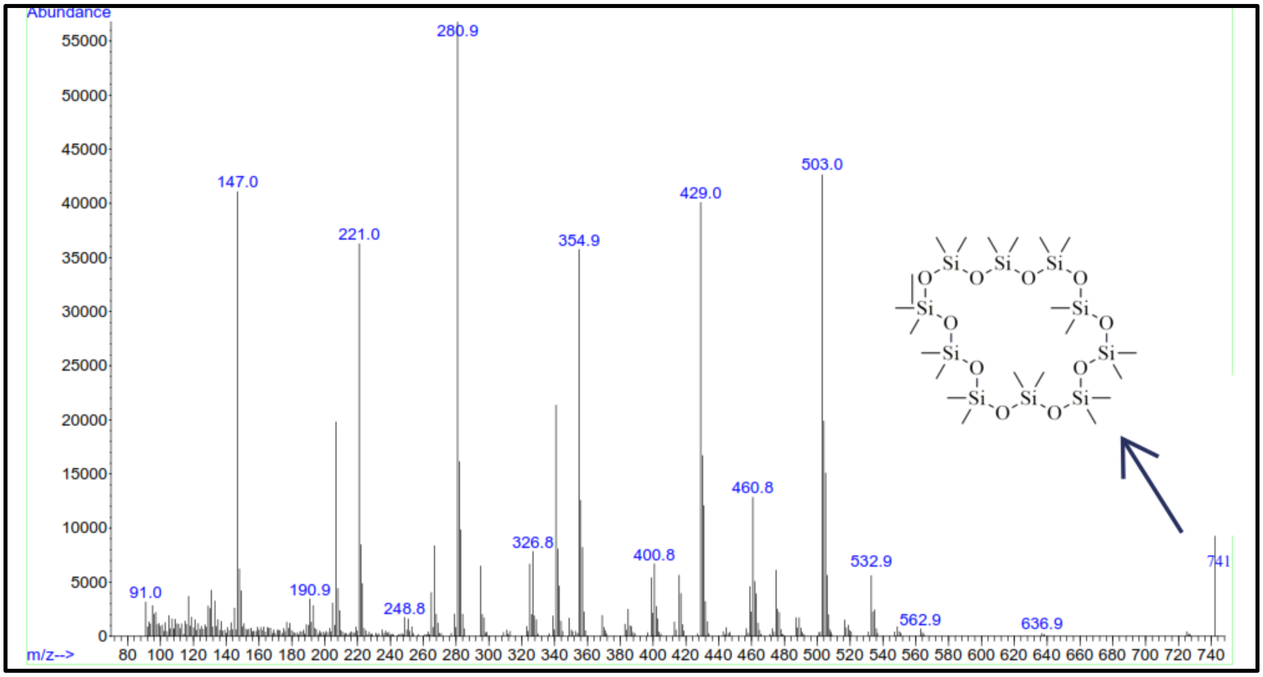

Figure S53: MS of aqueous extract of flower at 8.25 retention time.

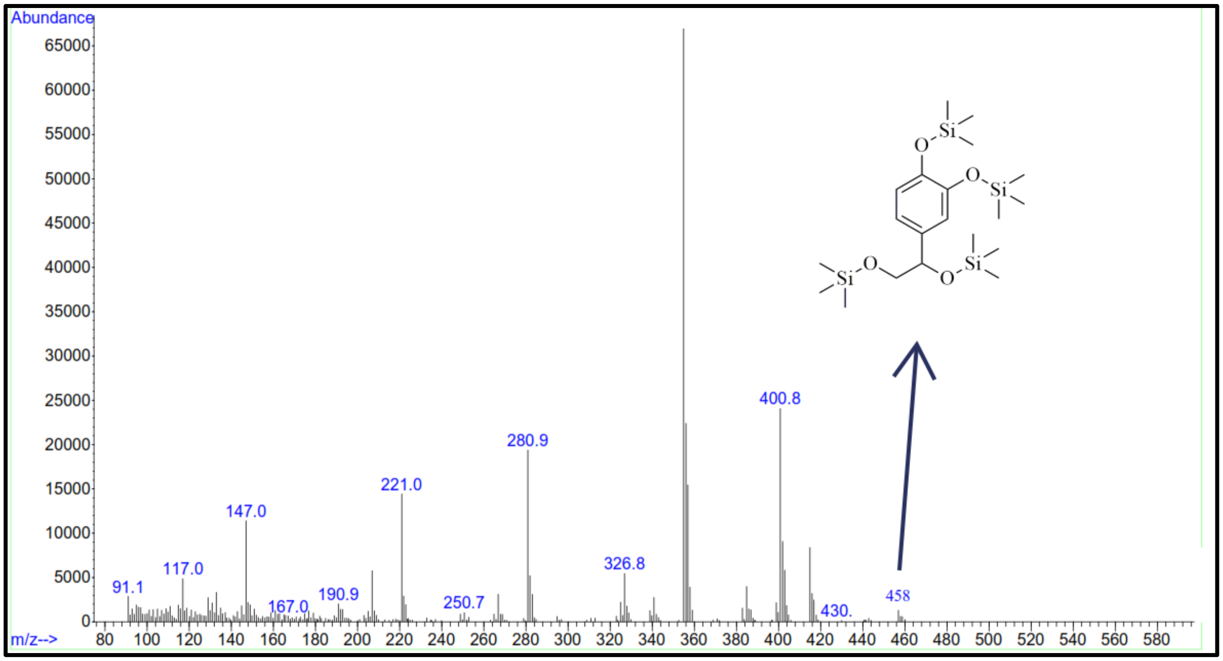

Figure S54: MS of aqueous extract of flower at 7.3 retention time

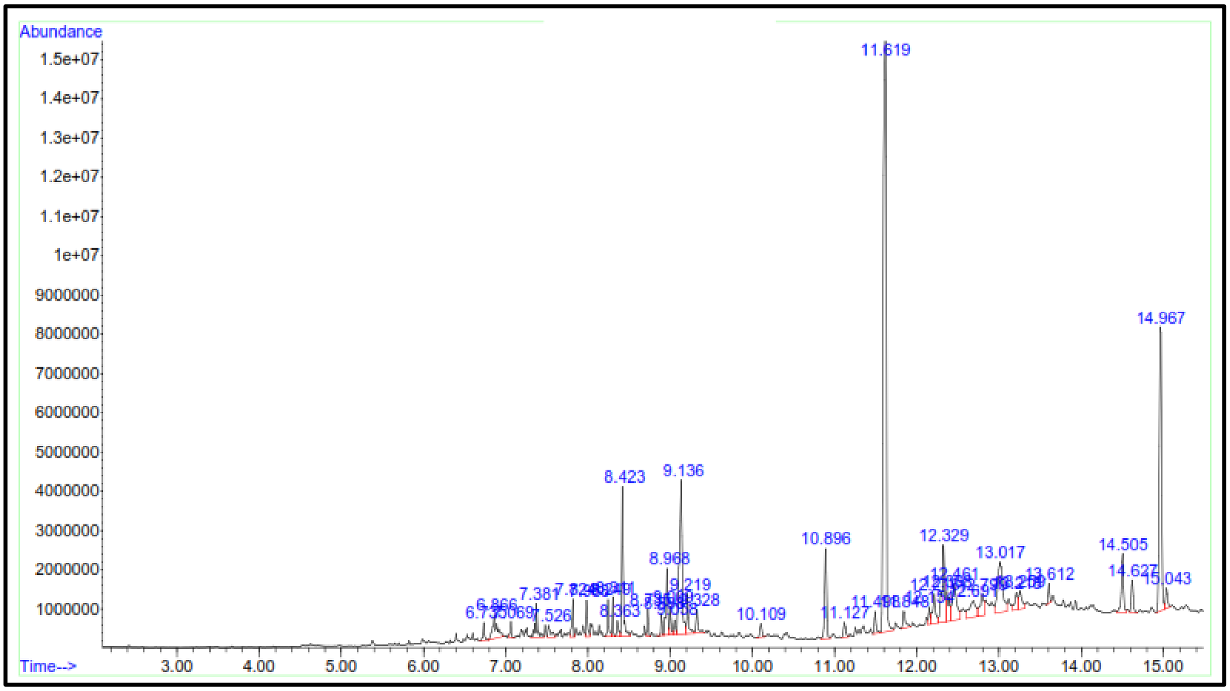

Figure S55: GC spectrum of ethyl acetate extract of mix flower, stem, leaf, and root.

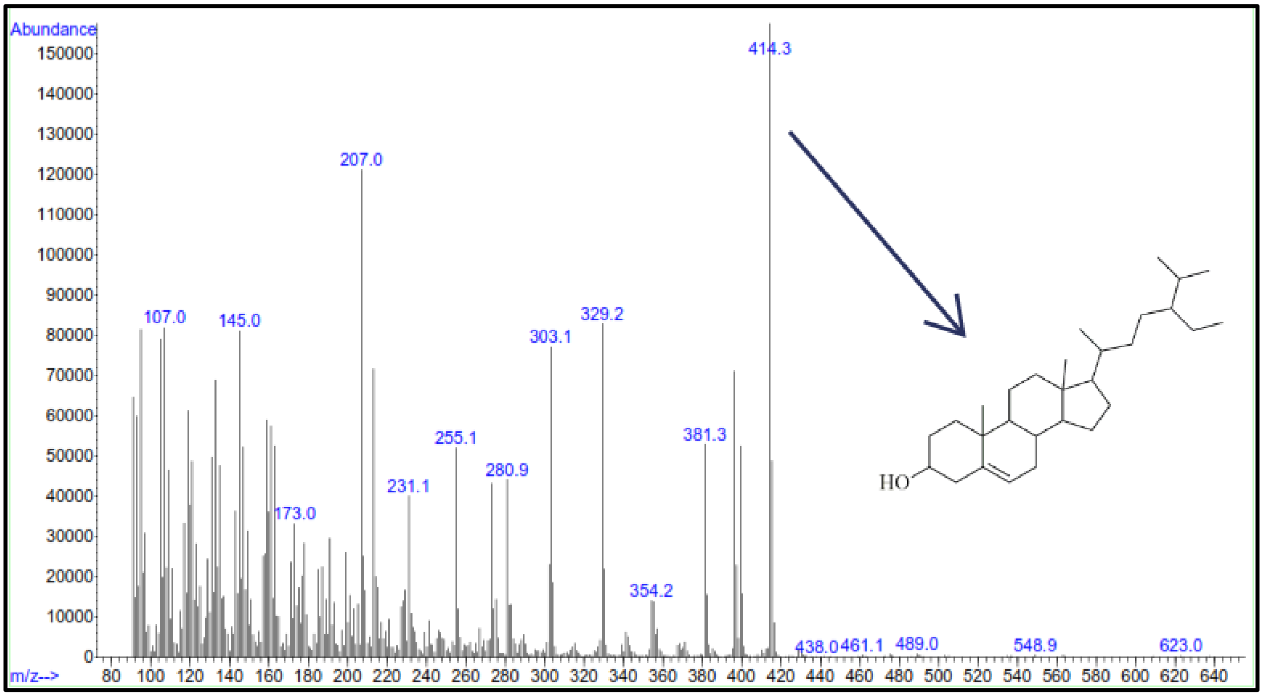

Figure S56: MS of ethyl acetate extract of mix flower, stem, leaf, and root at 14.9 retention time.

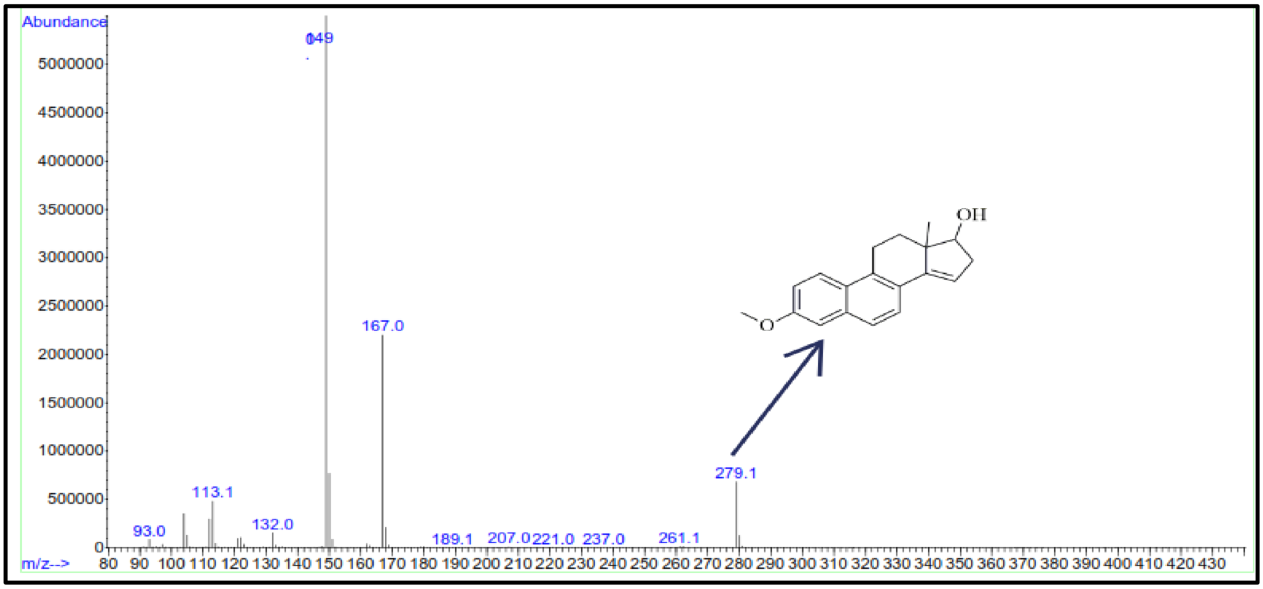

Figure S57: MS of ethyl acetate extract of mix flower, stem, leaf, and root at 11.6 retention time.

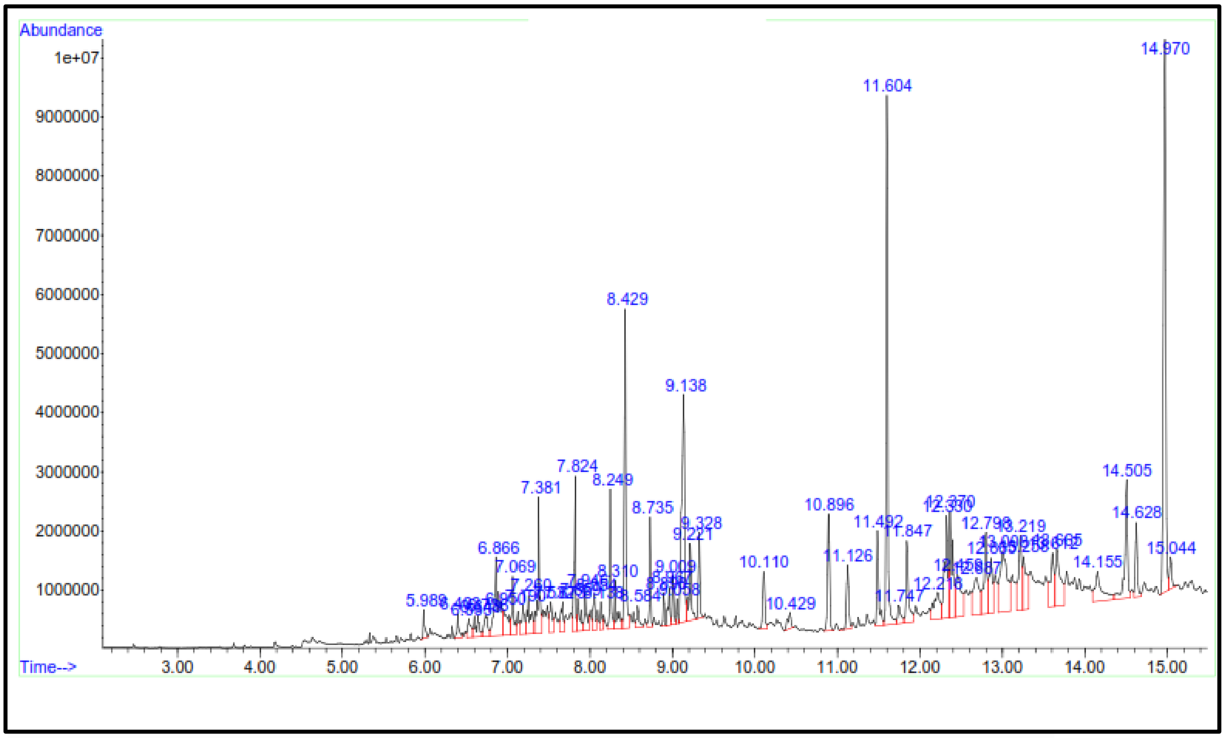

Figure S58: GC spectrum of ethanolic extract of mix flower, stem, leaf, and root.

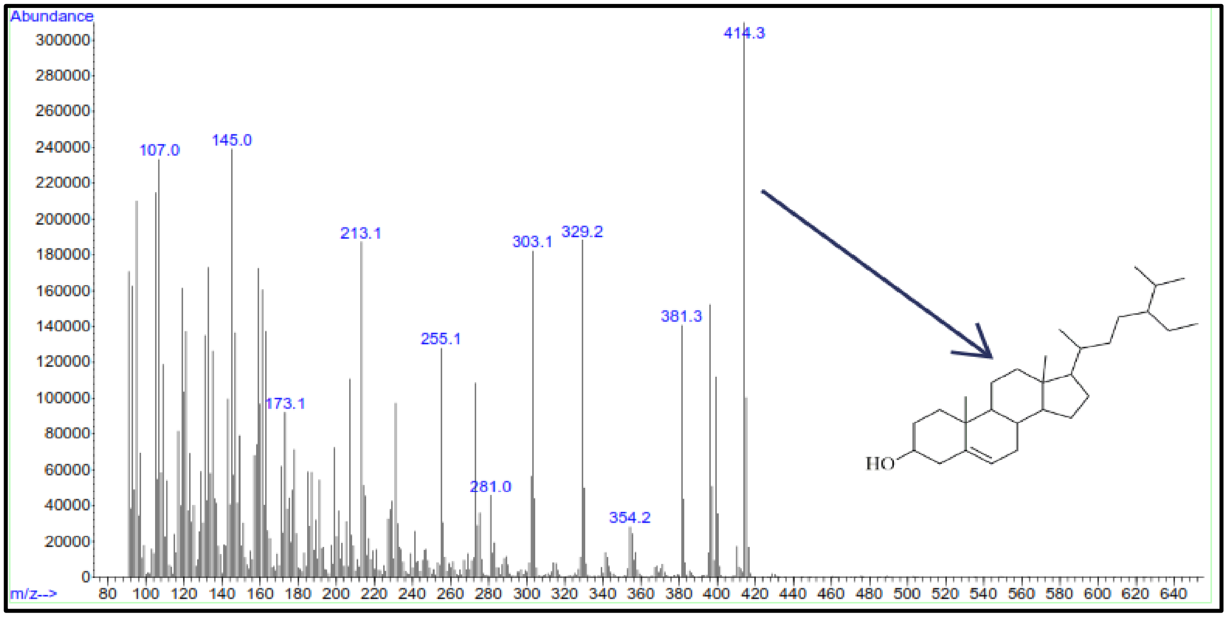

Figure S59: MS of ethanolic extract of mix flower, stem, leaf, and root at 14.9 retention time.

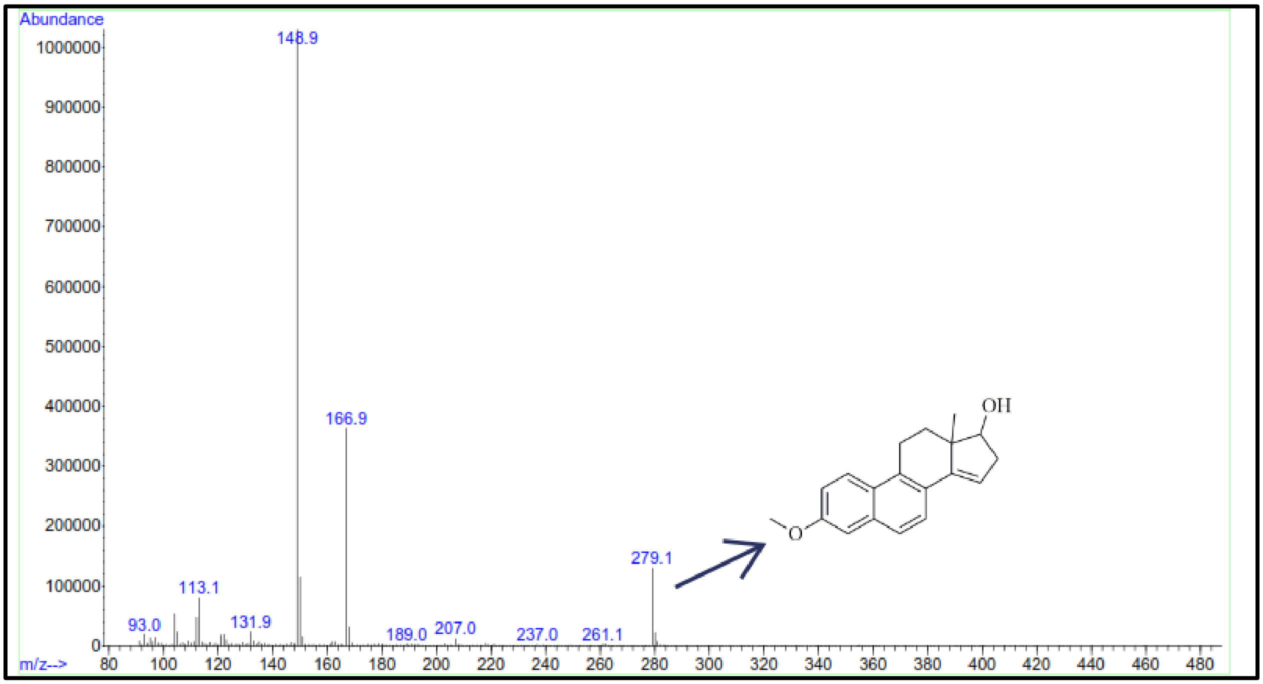

Figure S60: MS of ethanolic extract of mix flower, stem, leaf, and root at 11.6 retention time.

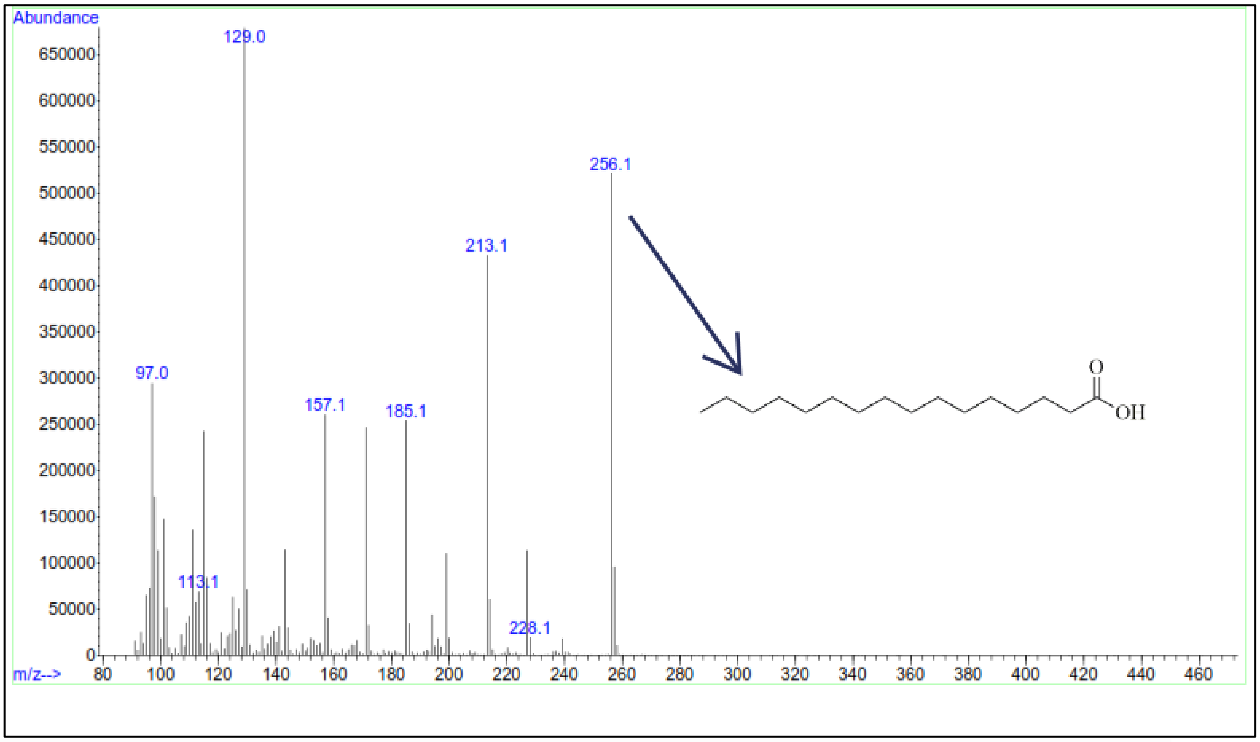

Figure S61: MS of aqueous extract of mix flower, stem, leaf, and root at 8.4 retention time.

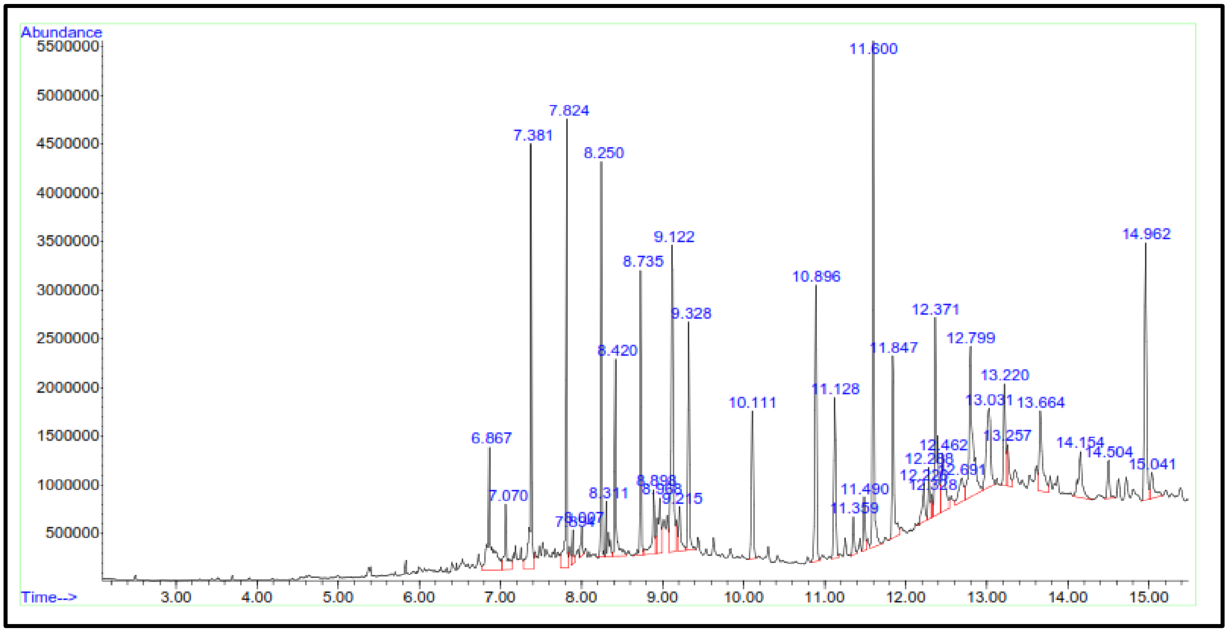

Figure S62: GC spectrum of aqueous extract of mix flower, stem, leaf, and root.

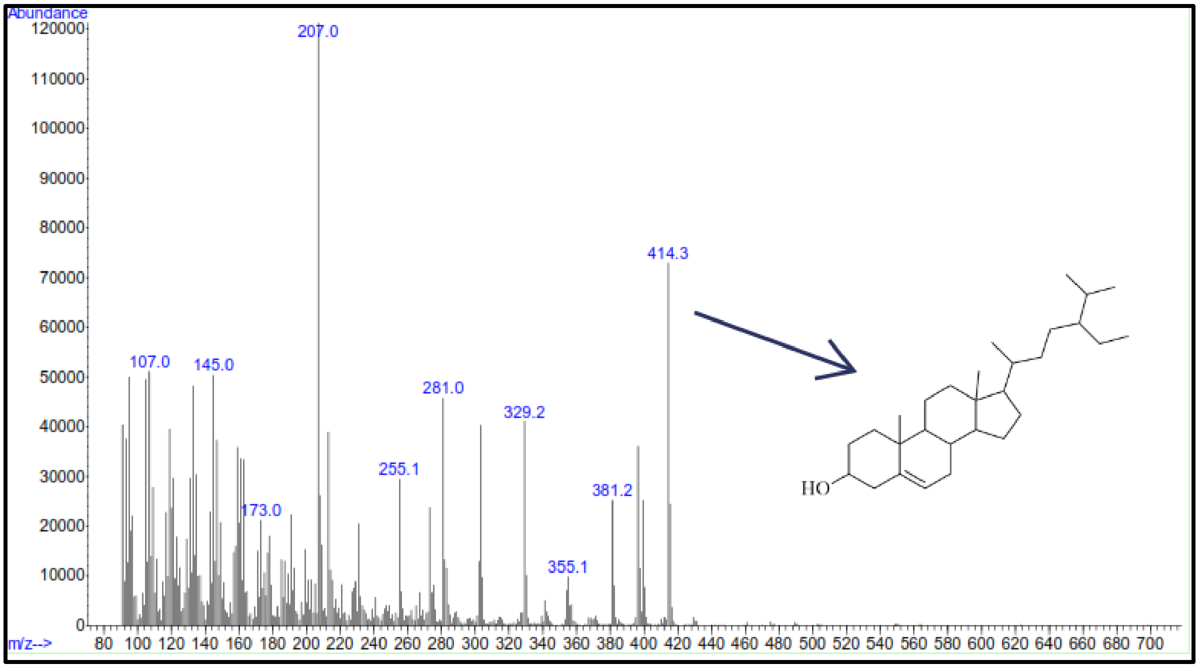

Figure S63: MS of aqueous extract of mix flower, stem, leaf, and root at 14.9 retention time.

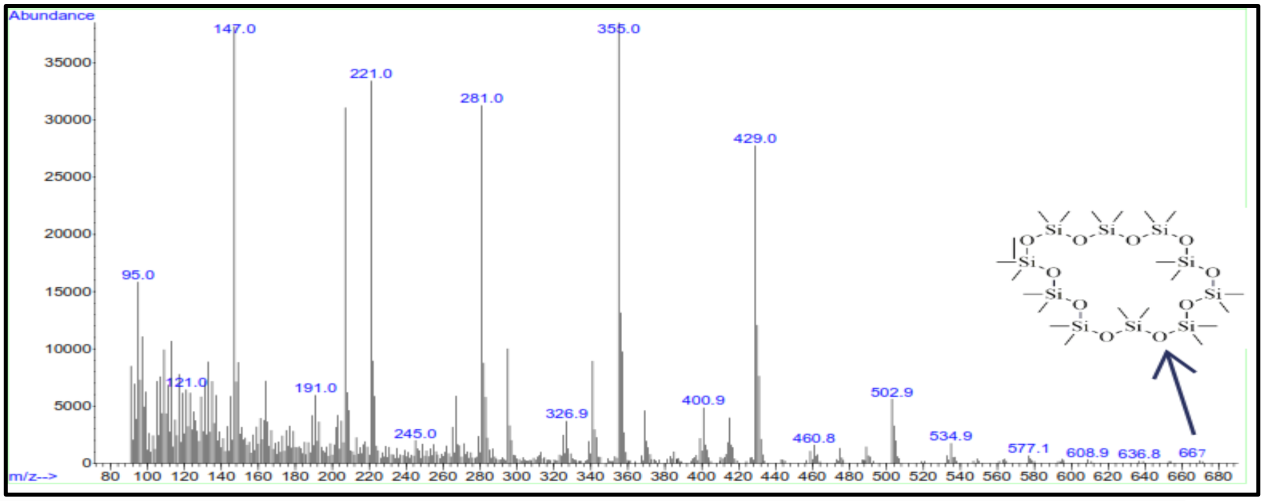

Figure S64: MS of aqueous extract of mix flower, stem, leaf, and root at 12.3 retention time.

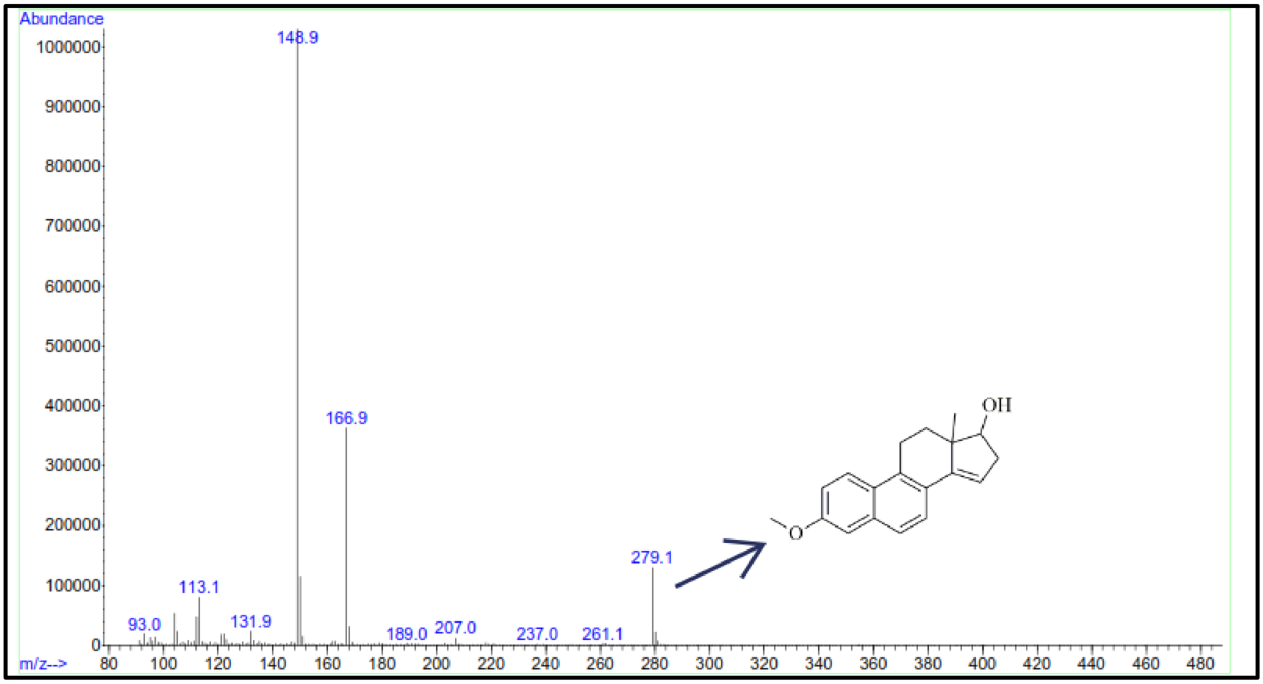

Figure S65: MS of ethyl aqueous extract of mix flower, stem, leaf, and root at 11.6 retention time.

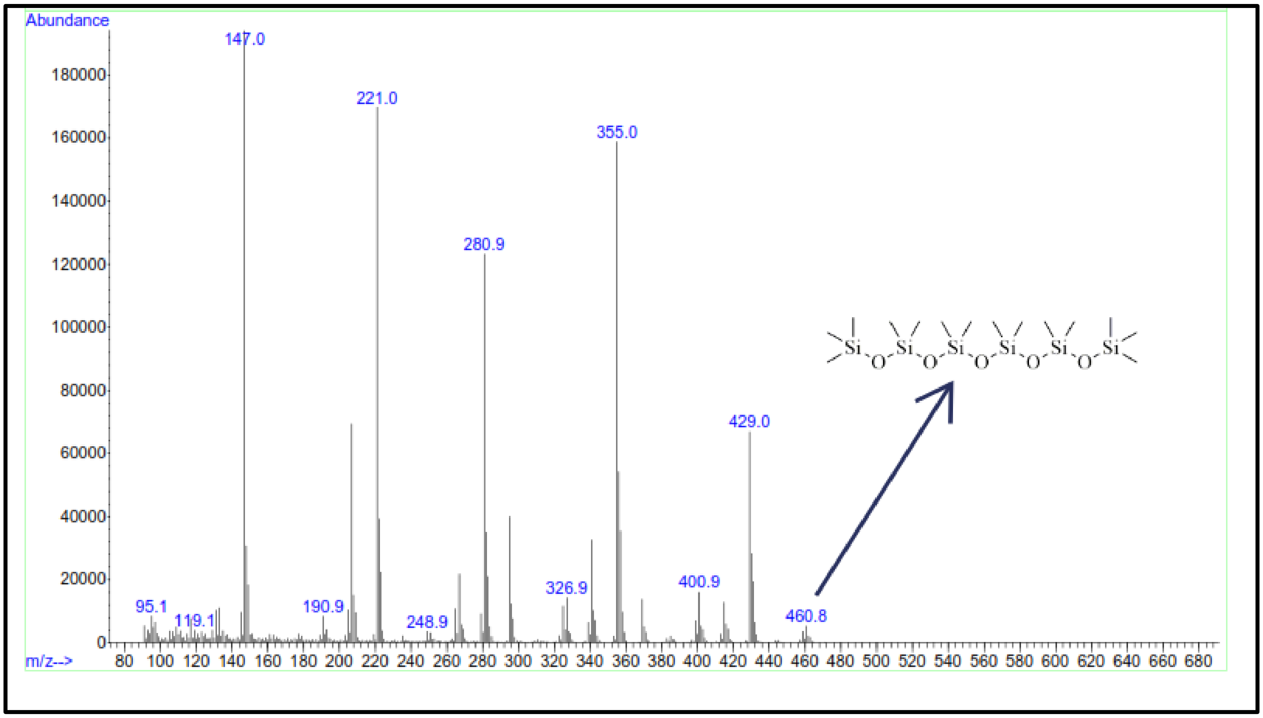

Figure S66: MS of aqueous extract of mix flower, stem, leaf, and root at 11.2 retention time.

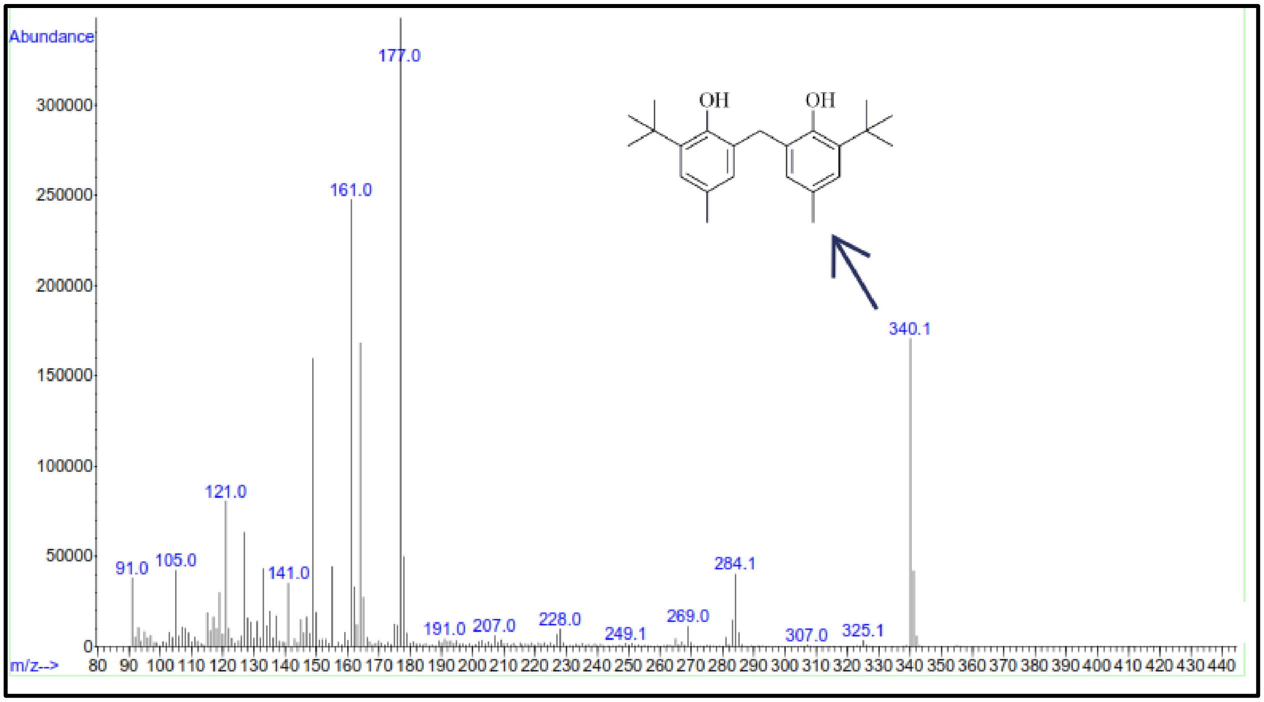

Figure S67: MS of aqueous extract of mix flower, stem, leaf, and root at 10.9. retention time.

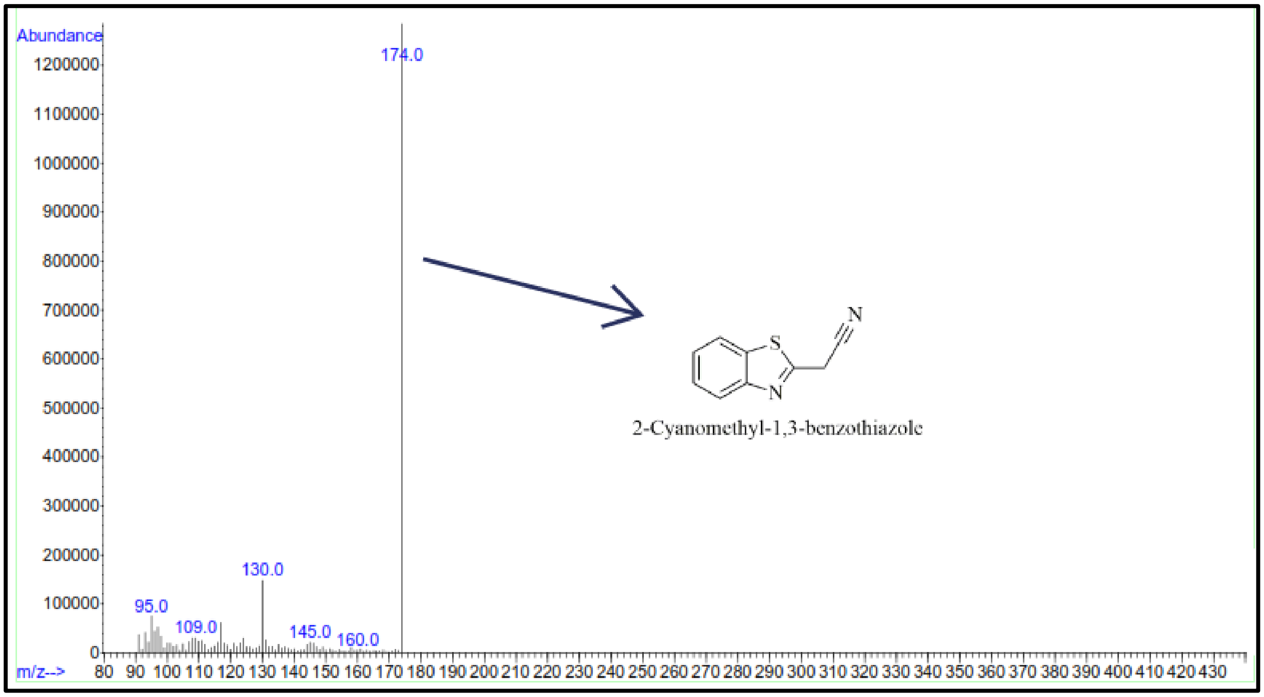

Figure S68: MS of aqueous extract of mix flower, stem, leaf, and root at 9.12 retention time.

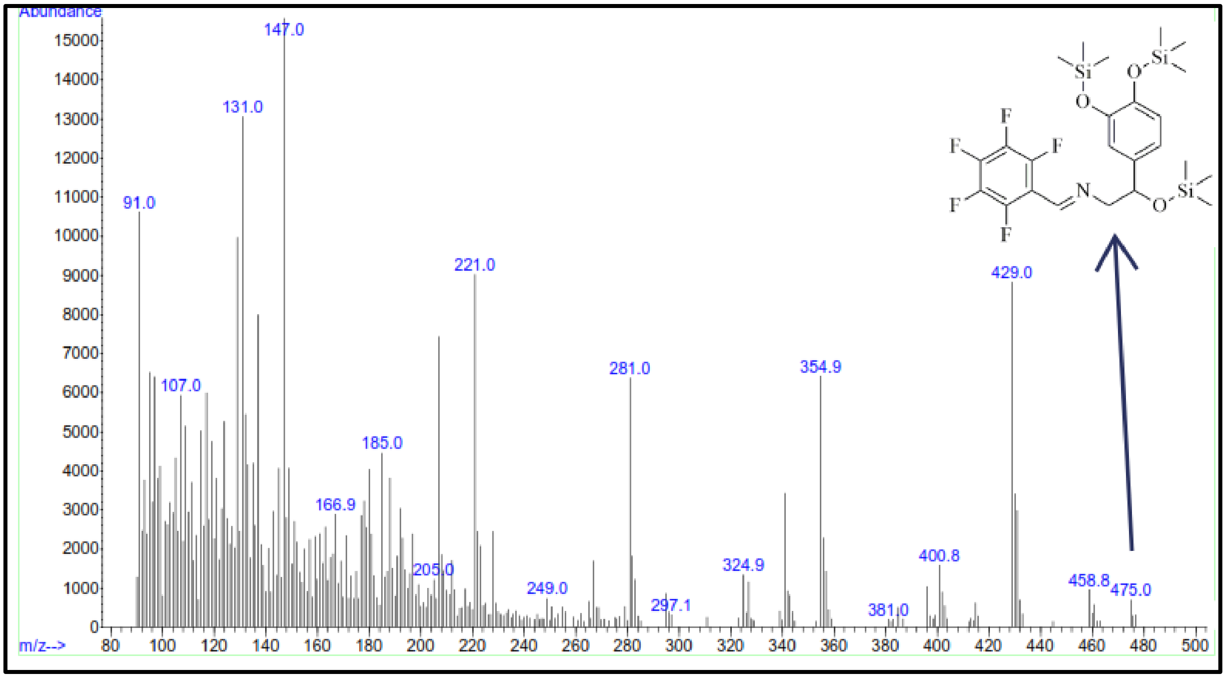

Figure S69: MS of aqueous extract of mix flower, stem, leaf, and root at 7.8 retention time.

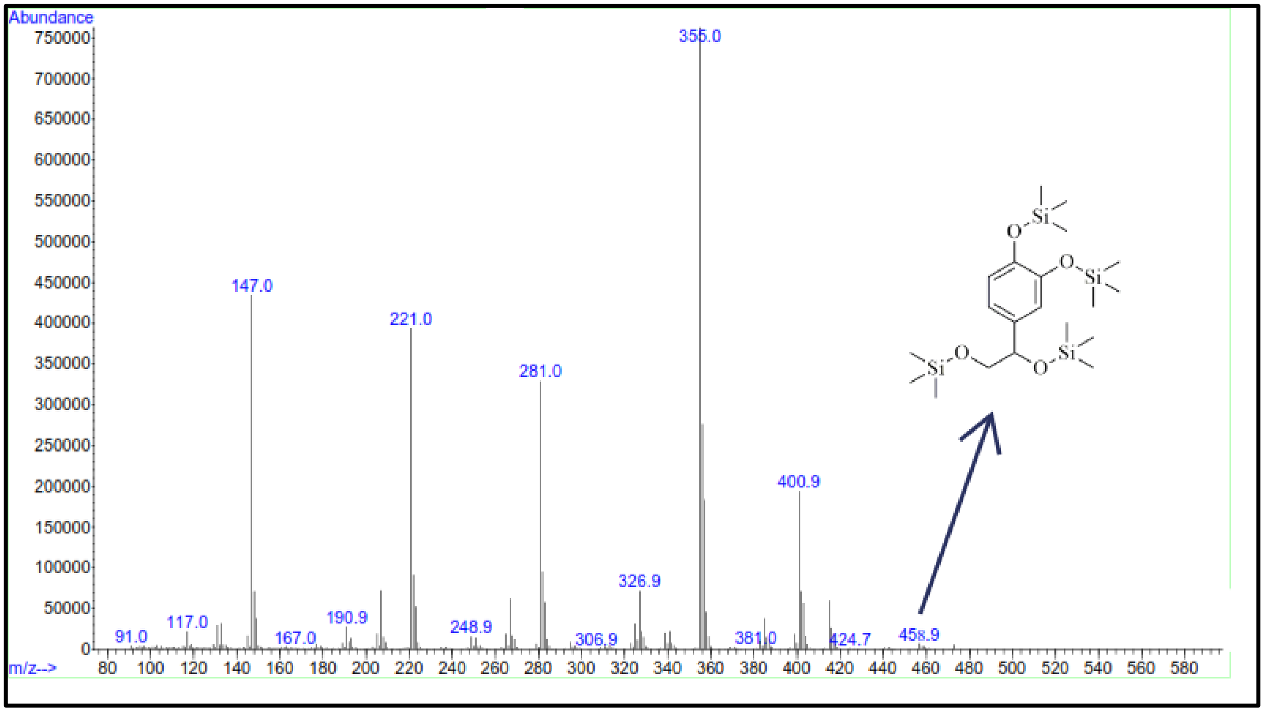

Figure S70: MS of aqueous extract of mix flower, stem, leaf, and root at 7.3 retention ti

Table S1. The identification of phytochemical in ethyl acetate, aqueous and ethanolic extract of root.

**GC-MS study of *ethyl acetate* extract of root**						**GC-MS study of *aqueous* extract of root**					
**Sr. No.**	**Ret. time**	**Name of compound**	**Molecular formula**	**% Area**	**MW g/mol**	**Sr. No.**	**Ret. time**	**Name of compound**	**Molecular formula**	**% Area**	**MW g/mol**
1	14.509	5-Cholestene-3-ol, 24-methyl	$C_{28}H_{48}O$	2.90	386.7	1	14.961	γ-Sitosterol	$C_{29}H_{50}O$	6.66	414.7067
2	14.509	Ergost-7-en-3-ol, (3.beta.)	$C_{28}H_{48}O$	2.90	400.7	5	13.346	Anthracene, 9,10-diethyl-9,10-dihyro	$C_{16}H_{16}$	7.16	208.3
3	11.599	Bis(2-ethylhexyl) phthalate	$C_{24}H_{38}O_4$	4.20	390.6	6	12.329	Linolenic acid, 2-hydroxy-1-(hydroxymethyl)ethyl ester	$C_{21}H_{36}O_4$	3.88	352.5081
4	9.330	Cyclononasiloxane, octadecamethyl	$C_{18}H_{54}O_9Si_9$	2.62	667.4	7	12.329	Ethyl 9,12,15-octadecatrienoate	$C_{20}H_{34}O_2$	3.88	306.4828
5	9.330	*Hexasiloxane, tetradecamethyl*	$C_{14}H_{42}O_5Si_6$	2.62	458.99	8	12.329	alpha-Linolenic acid, trimethylsilyl ester	$C_{21}H_{38}O_2Si$	3.88	306.4828
6	9.330	*3,6-Dioxa-2,4,5,7-tetrasilaoctane2,2,4,4,5,5,7,7-octamethyl*	$C_{10}H_{30}O_2Si_4$	2.62	294.686	9	11.599	Bis(2-ethylhexyl) phthalate	$C_{24}H_{38}O_4$	4.20	390.6
7	8.736	*1,3,5,7-Tetraethyl-1-*	$C_{14}H_{34}O_6Si_5$	1.73	438.84	10	9.133	*9,12,15-Octadecatrienoic acid*	$C_{18}H_{30}O_2$	9.38	278.43

		ethylbutoxysiloxycyclotetrasiloxane									
8	8.736	Cyclodecasiloxane, eicosamethyl-	$C_{20}H_{60}O_{10}Si_{10}$	1.73	741.5	11	9.133	9,12,15-Octadecatrien-1-ol	$C_{18}H_{32}O$	9.38	264.4
9	8.437	Lumiflavine	$C_{13}H_{12}N_4O_2$	4.29	256.26	14	8.969	9,12,15-octadecatrienoic acid, methyl ester	$C_{19}H_{32}O_2$	8.71	292.5
10	8.437	Adipic acid, 3-hexyl isobutyl ester	$C_{16}H_{30}O_4$	4.29	286.4070	15	8.969	9 12 15-octadecatrienoic acid ethyl ester	$C_{20}H_{34}O_2$	8.71	306.4828
11	8.437	n-Hexadecanoic acid	$C_{16}H_{32}O_2$	4.29	256.43	16	8.421	Adipic acid, 3-hexyl isobutyl ester	$C_{16}H_{30}O_4$	2.64	286.4070
12	8.250	Tetradecamethylhexasiloxane	$C_{14}H_{42}O_5Si_6$	2.33	458.99	18	8.311	1-Phenylcyclopentanenitrile	$C_{12}H_{13}N$	1.64	171.2383
13	8.250	Cyclodecasiloxane, eicosamethyl-	$C_{20}H_{60}O_{10}Si_{10}$	2.33	741.5	19	7.401	2-[2-[2-[2-[2-[2-[2-[2-[2-[2(Trimethylsilyloxy)ethoxy]ethoxy]ethoxy]ethoxy]ethoxy]ethoxy]ethoxy]ethoxy]ethoxy]ethanol	$C_8H_{18}O$	0.87	210.29
14	8.250	Cyclononasiloxane, octadecamethyl	$C_{18}H_{54}O_9Si_9$	2.33	667.4	20	2.474	1,6-Heptadiyne	C_7H_8	1.42	92.14

15	7.824	Benzoic acid, 4-hydroxy-3,5-dimethoxy-, hydrazide	$C_9H_{12}N_2O_4$	0.42	212.2026						
16	7.824	5-(Methylthio)-Salicylic acid, O,O'-dimethyl-	$C_{14}H_{24}O_3SSi_2$	0.42	328.58						
17	7.824	Pyrido[1,2-a][1,3]benzimidazole-7-carboxylic acid	$C_{12}H_7N_2O_2^-$	0.42	211.2						

GC-MS study of *ethanolic* extract of root

Sr. No.	Ret. time	Name of compound	Molecular formula	% Area	MW g/mol
1	14.972	γ-Sitosterol	$C_{29}H_{50}O$	2.79	414.7067
5	11.599	Bis(2-ethylhexyl) phthalate	$C_{24}H_{38}O_4$	4.20	390.6
6	11.599	*phthalic acid di*(*2-propylpentyl*)*ester*	$C_{24}H_{38}O_4$	4.20	390.6
7	10.894	phenol 2 2'-methylenebis 6-(1 1-dimethylethyl)-4-methyl-	$C_{23}H_{32}O_2$	2.93	340.4990
8	10.894	4-Methylthiophene-2-sulfonamide	$C_5H_7NO_2S_2$	2.93	177.2
10	9.129	9,12,15-octadecatrienoic acid, methyl ester	$C_{19}H_{32}O_2$	8.67	292.5
11	8.423	Pentadecanoic acid, methyl ester	$C_{16}H_{32}O_2$	5.34	256.4241

13	8.423	*n-Hexadecanoic acid*	$C_{16}H_{32}O$	5.34	256.4241

Table S2. The identification of phytochemical in ethyl acetate, aqueous and ethanolic extract of stem

**GC-MS study of *ethyl acetate* extract of stem**						**GC-MS study of *aqueous* extract of stem**					
**Sr. No.**	**Ret. time**	**Name of compound**	**Molecular formula**	**% Area**	**MW g/mol**	**Sr. No.**	**Ret. time**	**Name of compound**	**Molecular formula**	**% Area**	**MW g/mol**
2	14.984	beta-Sitosterol	$C_{29}H_{50}O$	11.75	414.71	1	14.96	γ-Sitosterol	$C_{29}H_{50}O$	6.68	414.70
4	14.510	5-Cholestene-3-ol, 24-methyl-	$C_{28}H_{48}O$	3.58	386.7	3	12.398	Octadecanoic acid, 2,3-dihydroxypropyl ester	$C_{25}H_{46}O_6$	3.21	442.6
5	14.510	Ergost-7-en-3-ol, (3.beta.)-	$C_{28}H_{48}O$	3.58	400.7	4	12.398	Octadecanoic acid, 2-hydroxy-1-(hydroxymethyl)ethyl ester	$C_{21}H_{42}O_4$	3.21	358.5558
6	11.652	Hexadecanoic acid, 2-hydroxy-1-(hydroxymethyl)ethyl ester	$C_{19}H_{38}O_4$	0.86	330.5026	5	11.599	Bis(2-ethylhexyl) phthalate	$C_{24}H_{38}O_4$	2.84	390.6

7	11.652	Glycerol 1-palmitate	$C_{19}H_{38}O_4$	0.86	330.5026	6	11.599	*phthalic acid di(2-propylpentyl)ester*	$C_{24}H_{38}O_4$	2.84	390.6
9	10.899	phenol 2 2'-methylenebis 6-(1 1-dimethylethyl)-4-methyl-	$C_{23}H_{32}O_2$	1.54	340.4990	8	9.136	9,12,15-octadecatrienoic acid, methyl ester	$C_{19}H_{32}O_2$	5.37	292.5
10	9.143	*9,12,15-Octadecatrienoic acid*	$C_{18}H_{30}O_2$	5.18	278.43	9	9.136	9,12,15-Octadecatrienoic acid	$C_{18}H_{30}O_2$	5.37	278.43
11	9.143	9,12,15-Octadecatrien-1-ol	$C_{18}H_{32}O$	5.18	264.4 g/mol	10	8.584	Phenazine, 2-methoxy-	$C_{13}H_{10}N_2O$	2.60	210.23
12	8.435	n-Hexadecanoic acid	$C_{16}H_{32}O_2$	3.11	256.43	11	8.584	3-Phenylbicyclo(3.2.2) nona-3,6-dien-2-one	$C_{15}H_{14}O$	2.60	210.27
13	8.435	Adipic acid, 3-hexyl isobutyl ester	$C_{16}H_{30}O_4$	3.11	286.4070	12	8.584	Benzene, 1,1'-butylidenebis	$C_{16}H_{18}$	2.60	210.3141
14	8.435	Lumiflavin	$C_{13}H_{12}N_4O_2$	3.11	256.26	14	8.422	2-Mercaptobenzothiazole	$C_7H_5NS_2$	0.70	167.25

**GC-MS study of *ethanolic* extract of stem**						15	8.249	Octasiloxane,1,1,3,3,5,5,7,7,9,9,11,11,13,13,15,15-hexadecamethyl-	$C_{16}H_{50}O_7Si_8$	1.53	577.2
1	14.985	γ-Sitosterol	$C_{29}H_{50}O$	17.51	414.70	16	8.249	Trisiloxane, 1,1,1,5,5,5-hexamethyl-3,3-bis[(trimethylsilyl)oxy]-	$C_{12}H_{36}O_4Si_5$	1.53	384.8393
4	14.507	5-Cholestene-3-ol, 24-methyl-	$C_{28}H_{48}O$	5.20	386.7	17	8.249	Pentasiloxane, dodecamethyl	$C_{10}H_{30}O_5Si_5$	1.53	370.77
6	11.601	3-Chlorocinnamic Acid	$C_9H_7ClO_2$	0.25	182.6	18	7.807	Cyclononasiloxane, octadecamethyl	$C_{18}H_{54}O_9Si_9$	4.76	667.4
7	11.601	Cinnamic acid, 3,4-dimethoxy-,trimethylsilyl ester	$C_{14}H_{20}O_4Si$	0.25	280.39	19	7.807	tert-Butyl-[2-(tertbutyldimethylsilyl)oxy-1-phenylethoxy]dimethylsilane	C20H38O2Si2	4.76	366.69
8	9.143	Octadecanoic acid	$C_{18}H_{36}O_2$	1.61	284.48	20	7.807	Mercaptoacetic acid, bis(trimethylsilyl)-	$C_8H_{20}O_2SSi_2$	4.76	236.479
10	8.435	2-Oxa-6-azatricyclo [3.3.1.1(3,7)] decane-6-carboxaldehyde	$C_9H_{13}NO_2$	0.37	167.2						

11	8.435	2-Mercaptobenzothiazole	$C_7H_5NS_2$	0.37	167.25						

Table S3. The identification of phytochemical in ethyl acetate, ethanolic and aqueous extract of leaf

**GC-MS study of *ethyl acetate* extract of leaf**						**GC-MS study of *ethanolic* extract of leaf**					
**Sr. No.**	**Ret. time**	**Name of compound**	**Molecular formula**	**% Area**	**MW g/mol**	**Sr. No.**	**Ret. time**	**Name of compound**	**Molecular formula**	**% Area**	**MW g/mol**
1	14.970	γ-Sitosterol	$C_{29}H_{50}O$	8.70	414.70	1	14.973	γ-Sitosterol	$C_{29}H_{50}O$	10.97	414.70
3	13.612	gamma-Tocopherol	$C_{28}H_{48}O_2$	1.85	416.68	3	13.937	Vitamin E	$C_{29}H_{50}O_2$	2.47	430.7
4	13.612	Beta-tocopherol	$C_{28}H_{48}O_2$	1.85	416.68	4	13.937	α-Tocopherol	$C_{29}H_{50}O_2$	2.47	430.71
5	13.612	delta-Tocopherol	$C_{27}H_{46}O_2$	1.85	402.7	5	13.612	gamma-Tocopherol	$C_{28}H_{48}O_2$	4.29	416.68
7	11.625	Bis(2-ethylhexyl) phthalate	$C_{24}H_{38}O_4$	18.01	390.6	6	13.612	Beta-tocopherol	$C_{28}H_{48}O_2$	4.29	416.68

8	9.146	9,12,15-octadecatrienoic acid	$C_{18}H_{30}O_2$	6.81	278.43	7	12.332	9,12,15-Octadecatrienoic acid, ethyl ester, (Z,Z,Z)-	$C_{20}H_{34}O_2$	3.92	306.4828
9	9.144	9,12,15-Octadecatrien-1-ol	$C_{18}H_{32}O$	6.81	264.4	9	11.603	Bis(2-ethylhexyl) phthalate	$C_{24}H_{38}O_4$	4.73	390.6
10	8.429	n-Hexadecanoic acid	$C_{16}H_{32}O_2$	3.38	256.43	10	11.603	Diisooctyl phthalate	$C_{24}H_{38}O_4$	4.73	390.55
11	8.429	Pentadecanoic acid, methyl ester	$C_{16}H_{32}O_2$	3.38		11	10.897	phenol 2 2'-methylenebis 6-(1 1-dimethylethyl)-4-methyl-	$C_{23}H_{32}O_2$	3.72	340.4990
12	8.250	Octasiloxane, 1,1,3,3,5,5,7,7,9,9,11,11,13,13,15,15-hexadecamethyl-	$C_{16}H_{48}O_7Si_8$	1.44		12	9.153	*9,12,15-Octadecatrienoic acid*	$C_{18}H_{30}O_2$	9.81	278.43
13	8.250	Trisiloxane, 1,1,1,5,5,5-hexamethyl-3,3-bis[(trimethylsilyl)oxy]-	$C_{12}H_{36}O_4Si_5$	1.44		13	8.431	n-Hexadecanoic acid	$C_{16}H_{32}O_2$	4.76	256.43256
14	7.82	Cyclononasiloxane, octadecamethyl-	$C_{18}H_{54}O_9Si_9$	1.51		14	8.431	Pentadecanoic acid, methyl ester	$C_{16}H_{32}O_2$	4.76	256.4241
15	7.82	Benzeneethanamine, N-	$C_{21}H_{26}F_5NO_2Si_2$	0.70							

		[(pentafluorophenyl) methylene]- .beta.,3,4- tris[(trimethylsilyl)oxy]-									
16	7.82	3,6-Dibutyl-2,5-dimethylpyrazine	$C_{14}H_{24}N_2$	0.70							
17	7.82	2-Pentene-1,4-dione, 1-(1,2,2-trimethylcyclopentyl	$C_{13}H_{20}O_2$	0.70							
18	7.381	Cyclooctasiloxane, hexadecamethyl-	$C_{16}H_{48}O_8Si_8$	2.39							
19	7.381	Silane, [[4-[1,2-bis[(trimethylsilyl)oxy]ethyl]-1,2-phenylene]bis(oxy)] bis[trimethyl-	$C_{20}H_{42}O_4Si_4$	2.39							

**GC-MS study of *ethanolic* extract of leaf**					
1	14.958	γ-Sitosterol	$C_{29}H_{50}O$	7.18	414.7067
4	11.598	Bis(2-ethylhexyl) phthalate	$C_{24}H_{38}O_4$	10.15	390.6

7	9.122	4-(p-Methoxyphenyl)-3-buten-2-onethiosemicarbazone	$C_{12}H_{15}N_3OS$	16.48	249.33
8	8.967	9,12,15-octadecatrienoic acid, methyl ester	$C_{19}H_{32}O_2$	5.37	292.5
9	8.967	Ethyl 9,12,15-octadecatrienoate	$C_{20}H_{34}O_2$	5.37	306.4828
10	8.967	Butyl 9,12,15-octadecatrienoate	$C_{22}H_{38}O_2$	5.37	334.5
11	8.418	n-Hexadecanoic acid	$C_{16}H_{32}O_2$	3.97	256.43 256
12	8.418	Adipic acid, 3-hexyl isobutyl ester	$C_{16}H_{30}O_4$	3.97	286.4070

Table S4. Phytoconstituents identified in ethyl acetate, ethanolic and aqueous extract of flower by GC-MS analysis

**GC-MS study of *ethyl acetate* extract of flower**						**GC-MS study of *ethanolic* extract of flower**					
**Sr. No.**	**Ret. time**	**Name of compound**	**Molecular formula**	**% Area**	**MW g/mol**	**Sr. No.**	**Ret. time**	**Name of compound**	**Molecular formula**	**% Area**	**MW g/mol**
1	14.9	Gamma-Sitosterol	$C_{29}H_{50}O$	0.95	414.7	1	14.958	γ-Sitosterol	$C_{29}H_{50}O$	8.70	414.70
3	14.6	Stigmasterol	$C_{29}H_{48}O$	0.95	412.69	3	14.629	Stigmasterol	$C_{29}H_{48}O$	1.35	412.69
4	14.6	Stigmasta-5,22-dien-3-ol, acetate,	$C_{31}H_{50}O_2$	0.95	454.7	4	14.629	Stigmasta-5,22-dien-3-ol, acetate, (3beta)-	$C_{31}H_{50}O_2$	1.35	454.7
5	14.5	Campesterol	$C_{28}H_{48}O$	1.46	400.68	5	14.506	Campesterol	$C_{28}H_{48}O$	1.82	400.0

7	13.037	Olean-12-en-28-oic acid, 3-hydroxy-, (3beta)-	$C_{30}H_{48}O_3$	0.69	456.7	6	12.866	1-Decanamine, N,N-didecyl-	$C_{30}H_{63}N$	2.29	437.8
8	12.4	2,3-dihydroxypropyl ester	$C_{14}H_{28}O_4$	0.95	260.37	7	12.866	Xanthine	$C_5H_4N_4O_2$	2.29	152.11
9	12.4	Octadecanoic acid	$C_{18}H_{36}O_2$	0.95	284.48	8	12.336	Methyl (Z)-5,11,14,17-eicosatetraenoate	$C_{21}H_{34}O_2$	4.03	318.5
10	12.4	2-hydroxy-1-(hydroxymethyl)ethyl ester	$C_{23}H_{46}O_4$	0.95	386.6	10	11.600	phthalic acid di(2-propylpentyl) ester	$C_{24}H_{38}O_4$	2.31	390.6
11	12.3	*9,12,15-Octadecatrienoic acid*	$C_{18}H_{30}O_2$	4.78	278.4	11	11.600	Bis(2-ethylhexyl) phthalate	$C_{24}H_{38}O_4$	2.31	390.6
12	12.3	Methyl (Z)-5,11,14,17-eicosatetraenoate	$C_{21}H_{34}O_2$	4.78	318.5	12	10.895	Phenol, 2,2'-methylenebis[6-(1,1-dimethylethyl)-4-methyl-	$C_{23}H_{32}O_2$	1.55	340.4990
13	11.653	Diisooctyl phthalate	$C_{24}H_{38}O_4$	14.96	390.55	13	10.109	Cyclononasiloxane, octadecamethyl	$C_{18}H_{54}O_9Si_9$	1.30	667.4
16	9.237	Octadecanoic acid	$C_{18}H_{36}O_2$	0.84	284.47	14	10.109	Tetradecamethylhexasiloxane	$C_{14}H_{42}O_5Si_6$	1.30	458.99

17	9.237	Adipic acid, isobutyl 2-methylbutyl ester	$C_{15}H_{28}O_4$	0.84	272.38	17	8.970	Butyl 9,12,15-octadecatrienoate	$C_{22}H_{38}O_2$	2.50	334.5
18	9.169	9,12,15-octadecatrienoic acid	$C_{18}H_{30}O_2$	6.34	278.43	18	8.970	9,12,15-octadecatrienoic acid, methyl ester	$C_{19}H_{32}O_2$	2.50	292.5
19	9.169	9,12,15-Octadecatrien-1-ol	$C_{18}H_{32}O$	6.34	264.4	19	8.735	1,3,5,7-Tetraethyl-1-ethylbutoxysiloxycyclotetrasiloxane	$C_{14}H_{34}O_6Si_5$	1.74	438.84
20	8.972	9,12,15-Octadecatrienoic acid, methyl ester,	$C_{19}H_{32}O_2$	0.60	292.5	20	8.735	Benzeneacetic acid, alpha,4-bis[(trimethylsilyl)oxy]-, trimethylsilyl ester	8.735	1.74	384.7
21	8.972	Ethyl 9,12,15-octadecatrienoate	$C_{20}H_{34}O_2$	0.60	306.4828	21	8.435	Pentadecanoic acid, methyl ester	$C_{16}H_{32}O_2$	2.92	256.4241
22	8.972	9,12,15-Octadecatrienoic acid, ethyl ester	$C_{20}H_{34}O_2$	0.60	306.4828	22	8.435	Adipic acid, 3-hexyl isobutyl ester	$C_{16}H_{30}O_4$	2.92	286.4070
23	8.437	Methyl pentadecanoate	$C_{16}H_{32}O_2$	2.86	256.4	23	8.435	n-Hexadecanoic acid	$C_{16}H_{32}O$	2.92	256.4241
24	8.437	N-Hexadecanoic acid	$C_{16}H_{32}O_2$	2.86	256.4241	24	8.249	Hexasiloxane, tetradeca methyl-	$C_{14}H_{42}O_5Si_6$	2.27	458.99

25	8.437	Adipic acid, isobutyl 2-methylpent-3-yl ester	$C_{16}H_{30}O_4$	2.86	286.41	25	7.824	Cyclononasiloxane, octadecamethyl-	$C_{18}H_{54}O_9Si_9$	2.59	667.4
26	8.311	7,9-Di-tert-butyl-1-oxaspiro [4.5] deca-6,9-diene-2,8-dione	$C_{17}H_{24}O_3$	0.47	276.4	26	7.381	Hexadecamethylcyclooctasiloxane	$C_{16}H_{48}O_8Si_8$	2.14	593.2
27	8.311	1-Ethyl-3, trans-(1,1-dimethylethyl)-4, cis-methoxycyclohexan-1-ol	$C_{13}H_{26}O_2$	0.47	214.34						
28	8.311	Z-10-Methyl-11-tetradecen-1-ol propionate	$C_{18}H_{34}O_2$	0.47	282.5						
30	6.865	Allopurinol	$C_5H_4N_4O$	1.85	136.11						
31	6.865	2,4-Dimethylanisole	$C_9H_{12}O$	1.85	136.19						
**GC-MS study of *aqueous* extract of flower**											
**Sr. No.**	**Ret. time**	**Name of compound**	**Molecular formula**	**% Area**	**MW g/mol**	**Sr. No.**	**Ret. time**	**Name of compound**	**Molecular formula**	**% Area**	**MW g/mol**
2	12.371	1,1,1,5,7,7,7-Heptamethyl-3,3-	$C_{13}H_{39}O_5Si_6$	3.10	443.96	12	8.735	1,1,1,5,7,7,7-Heptamethyl-3,3-	$C_{13}H_{39}O_5Si_6$	5.88	443.96

		bis(trimethylsiloxy)tetrasiloxane						bis(trimethylsiloxy)tetrasiloxane			
3	12.371	Octadecamethylcyclononasiloxane	$C_{18}H_{54}O_9Si_9$	3.10	667.4	13	8.735	1,3,5,7,9-Pentaethylbicyclo[5.3.1]pentasiloxane-3,5,9-triyl	$C_{10}H_{25}O_6Si_5$	5.88	381.0497
4	12.371	Tetradecamethylhexasiloxane	$C_{14}H_{42}O_5Si_6$	3.10	458.99	15	8.250	Dodecamethylpentasiloxane	$C_{12}H_{36}O_4Si_5$	7.24	384.84
5	11.848	1,1,1,5,7,7,7-Heptamethyl-3,3-bis(trimethylsiloxy)tetrasiloxane	$C_{13}H_{39}O_5Si_6$	3.86	443.96	16	7.824	Octadecamethylcyclononasiloxane	$C_{18}H_{54}O_9Si_9$	8.10	667.4
6	11.848	Octadecamethylcyclononasiloxane	$C_{18}H_{54}O_9Si_9$	3.86	667.4	17	7.381	Hexadecamethylcyclooctasiloxane	$C_{16}H_{48}O_8Si_8$	8.35	593.2
7	11.848	Tetradecamethylhexasiloxane	$C_{14}H_{42}O_5Si_6$	3.86	458.99	19	7.068	Phenol, 2,4-bis-(1,1-dimethylethyl)	$C_{17}H_{30}OSi$	2.10	278.5
8	11.126	2,2,4,4,5,5,7,7-Octamethyl-3,6-dioxa-2,4,5,7-tetrasilaoctane	$C_{10}H_{30}O_2Si_4$	3.71	294.686	20	7.068	Phenol, 2,6-bis(1,1-dimethylethyl)methyl-	$C_{15}H_{24}O$	2.10	220.35
10	10.109	Octadecamethylcyclononasiloxane	$C_{18}H_{54}O_9Si_9$	4.30	667.4	21	6.867	Tetradecamethylcycloheptasiloxane	$C_{14}H_{42}O_7Si_7$	3.50	519.07

11	10.109	1,1,1,5,7,7,7-Heptamethyl-3,3-bis(trimethylsiloxy)tetrasiloxane	$C_{13}H_{39}O_5Si_6$	4.30	443.96	22	6.867	Trisiloxane, 1,1,1,5,5,5-hexamethyl-3,3-bis[(trimethylsilyl)oxy]-	$C_{12}H_{36}O_4Si_5$	3.50	384.8393

Table S5. Phytoconstituents identified in ethyl acetate, ethanolic and aqueous extract of mix flower, stem, leaf, and root by GC-MS analysis

**GC-MS study of *ethyl acetate* extract of mix flower, stem, leaf, and root**						**GC-MS study of *ethanolic* extract of mix flower, stem, leaf, and root**					
**Sr. No.**	**Ret. time**	**Name of compound**	**Molecular formula**	**% Area**	**MW g/mol**	**Sr. No.**	**Ret. time**	**Name of compound**	**Molecular formula**	**% Area**	**MW g/mol**
1	14.967	γ-Sitosterol	$C_{29}H_{50}O$	12.48	414.70	1	14.970	γ-Sitosterol	$C_{29}H_{50}O$	11.61	414.70
2	14.505	Campesterol	$C_{28}H_{48}O$	2.46	400.68	2	14.505	Campesterol	$C_{28}H_{48}O$	2.96	400.68
3	14.505	5-Cholestene-3-ol, 24-methyl-	$C_{28}H_{48}O$	2.46	386.7	3	14.505	5-Cholestene-3-ol, 24-methyl-	$C_{28}H_{48}O$	2.96	386.7

4	12.329	Linolenic acid, 2-hydroxy-1-(hydroxymethyl)ethyl ester	$C_{21}H_{36}O_4$	3.55	352.5081	4	12.333	.alpha.-Linolenic acid, trimethylsilyl ester	$C_{21}H_{38}O_2Si$	2.67	350.6107
5	12.329	Ethyl 9,12,15-octadecatrienoate	$C_{20}H_{34}O_2$	3.55	306.4828	5	12.333	Linolenic acid, 2-hydroxy-1-(hydroxymethyl)ethyl ester	$C_{21}H_{36}O_4$	2.67	352.5081
6	11.619	Bis(2-ethylhexyl) phthalate	$C_{24}H_{38}O_4$	29.27	390.6	6	12.333	Ethyl 9,12,15-octadecatrienoate	$C_{20}H_{34}O_2$	2.67	306.4828
7	11.619	phthalic acid di(6-methylhept-2-yl) ester	$C_{24}H_{38}O_4$	29.27	390.6	7	11.604	phthalic acid di(2-propylpentyl) ester	$C_{24}H_{38}O_4$	8.33	390.6
8	10.896	phenol 2 2'-methylenebis 6-(1 1-dimethylethyl)-4-methyl-	$C_{23}H_{32}O_2$	3.20	340.4990	8	11.604	Bis(2-ethylhexyl) phthalate	$C_{24}H_{38}O_4$	8.33	390.6
9	9.136	*9,12,15-Octadecatrienoic acid*	$C_{18}H_{30}O_2$	8.11	278.43	9	10.896	phenol 2 2'-methylenebis 6-(1 1-dimethylethyl)-4-methyl-	$C_{23}H_{32}O_2$	3.20	340.4990
10	9.136	9,12,15-Octadecatrien-1-ol	$C_{18}H_{32}O$	8.11	264.4	10	9.138	*9,12,15-Octadecatrienoic acid*	$C_{18}H_{30}O_2$	6.01	278.43

11	8.968	Tetracosyl acetat	$C_{26}H_{52}O_2$	0.49	396.7	11	9.138	methyl 2-hydroxy-octadeca-9 12 15-trienoate	$C_{19}H_{32}O_3$	6.01	308.5
12	8.968	Acetic acid n-octadecyl ester	$C_{20}H_{40}O_2$	0.49	312.5304	12	8.735	18-Methyl-nonadecanol, trimethylsilyl ether	$C_{23}H_{50}OSi$	1.28	370.7
13	8.968	Nonadecyl penta-fluoropropionate	$C_{22}H_{39}F_5O_2$	0.49	430.5	13	8.735	Benzoic acid, 2,4-bis[(trimethylsilyl)oxy]-, trimethylsilyl ester	$C_{16}H_{30}O_4Si_3$	1.28	370.66
14	8.423	Adipic acid, 3-hexyl isobutyl ester	$C_{16}H_{30}O_4$	4.31	286.4070	14	8.429	Pentadecanoic acid, methyl ester	$C_{16}H_{32}O_2$	4.54	256.4241
15	8.423	n-Hexadecanoic acid	$C_{16}H_{32}O_2$	4.31	256.43	15	8.249	Hexasiloxane, tetradecamethyl-	$C_{14}H_{42}O_5Si_6$	1.55	458.9933
16	8.423	Pentadecanoic acid, methyl ester	$C_{16}H_{32}O_2$	4.31	256.4241	16	8.249	Octasiloxane, 1,1,3,3,5,5,7,7,9,911,11,13,13,15,15-hexadecamethyl-	$C_{16}H_{48}O_7Si_8$	1.55	577.2
						17	8.249	cycloheptasiloxane tetradecamethyl-	$C_{14}H_{42}O_7Si_7$	1.55	519.07
**GC-MS study of *aqueous* extract of mix flower, stem, leaf, and root**											
**Sr. No.**	**Ret. time**	**Name of compound**	**Molecular formula**	**%**	**MW g/mol**	**Sr. No.**	**Ret. time**	**Name of compound**	**Molecular formula**	**% Area**	**MW**

				Area							g/mol
1	14.962	γ-Sitosterol	$C_{29}H_{50}O$	5.45	414.7067	11	9.328	*3,6-Dioxa-2,4,5,7-tetrasilaoctane2,2,4,4,5,5,7,7-octamethyl*	$C_{10}H_{30}O_2Si_4$	3.59	294.686
2	12.799	Hexasiloxane, tetradecamethyl-	$C_{14}H_{42}O_5Si_6$	5.53	458.99	12	9.122	2-Cyanomethyl-1,3-benzothiazole	$C_9H_6N_2S$	6.98	174.222
3	12.799	3,6-Dioxa-2,4,5,7-tetrasilaoctane,2,2,4,4,5,5,7,7-octamethyl-	$C_{10}H_{30}O_2Si_4$	5.53	294.686	13	9.122	Thiocyanic acid, 1H-indol-3-yl ester	$C_9H_6N_2S$	6.98	174.22
4	12.799	Heptasiloxane, hexadecamethyl-	$C_{16}H_{48}O_6Si_7$	5.53	533.1	14	8.735	Benzoic acid, 2,4-bis[(trimethylsilyl)oxy]-, trimethylsilyl ester	$C_{16}H_{30}O_4Si_3$	1.28	370.66
5	12.371	Cyclononasiloxane, octadecamethyl	$C_{18}H_{54}O_9Si_9$	4.20	667.4	15	8.735	Cyclooctasiloxane, hexadecamethyl-	$C_{16}H_{48}O_8Si_8$	3.57	593.2
6	11.659	11H-Cyclopenta[a]phenanthren-17-ol, 12,13,16,17-tetrahydro-3-	$C_{19}H_{20}O_2$	0.25	280.4	16	8.250	cycloheptasiloxane tetradecamethyl-	$C_{14}H_{42}O_7Si_7$	3.91	519.07

		methoxy-13-methyl-, (13S-cis)-									
7	11.600	Bis(2-ethylhexyl) phthalate	$C_{24}H_{38}O_4$	7.52	390.6	17	8.250	Octasiloxane, 1,1,3,3,5,5,7,7,9,9,11,11,13,13,15,15-hexadecamethyl-	$C_{16}H_{48}O_7Si_8$	3.91	577.2
8	11.600	phthalic acid di(2-propylpentyl)ester	$C_{24}H_{38}O_4$	7.52	390.6	18	8.250	Pentasiloxane, dodecamethyl-	$C_{12}H_{36}O_4Si_5$	3.91	384.84
9	11.12	Hexasiloxane, tetradecamethyl-	$C_{14}H_{42}O_5Si_6$	3.19	458.99	19	7.824	Cyclononasiloxane, octadecamethyl-	$C_{18}H_{54}O_9Si_9$	5.54	667.4
10	9.328	*Hexasiloxane*, *tetradecamethyl*-	$C_{14}H_{42}O_5Si_6$	3.59	458.99	20	7.381	Cyclooctasiloxane, hexadecamethyl	$C_{16}H_{48}O_8Si_8$	5.10	593.2
